# Additional effects of herbal medicine combined with bisphosphonates for primary osteoporosis: a systematic review and meta-analysis

**DOI:** 10.3389/fphar.2024.1413515

**Published:** 2024-09-13

**Authors:** Young-Seo Yoo, Min-Gyeong Kim, Hee-Joo Park, Min-Young Chae, Yu-Jin Choi, Chae-Kun Oh, Chang-Gue Son, Eun-Jung Lee

**Affiliations:** ^1^ College of Korean Medicine, Daejeon University, Daejeon, Republic of Korea; ^2^ Department of Korean Rehabilitation Medicine, College of Korean Medicine, Daejeon University, Daejeon, Republic of Korea; ^3^ Institute of Bioscience and Integrative Medicine, Department of Korean Medicine, Daejeon University, Daejeon, Republic of Korea; ^4^ Department of Herbal Formula, College of Korean Medicine, Daejeon University, Daejeon, Republic of Korea

**Keywords:** primary osteoporosis, herbal medicines, bisphosphonates, combination therapy, bone mineral density

## Abstract

**Background:**

Osteoporosis (OP) is a significant medical issue associated with population aging. Recent research on herbal medicines (HMs) for OP has been increasing, with these therapies sometimes used in conjunction with bisphosphonates (BPs), the standard treatment for OP. We conducted a systematic review and meta-analysis to evaluate the effects of combining HMs with BPs on improving bone mineral density (BMD) in patients with primary OP.

**Methods:**

We searched nine databases—PubMed, Embase, Cochrane Library, China National Knowledge Infrastructure Wanfang, KISS, Kmbase, Science On, and Oasis—up to 31 August 2023. We selected randomized controlled trials (RCTs) comparing BMD between HMs *plus* BPs and BPs alone in primary OP. A meta-analysis with BMD as the primary outcome was performed using RevMan version 5.4. Study quality and evidence certainty were assessed through Cochrane’s risk of bias2 and GRADE.

**Results:**

Out of 43 RCTs involving 4,470 participants (mean age 65.8 ± 6.6 years), 35 RCTs with 3,693 participants were included in the meta-analysis. The combination of HMs and BPs was found to be more effective in improving BMD compared to BPs alone, with improvements of 0.10 g/cm^2^ at the lumbar spine (33 RCTs, 95% CI: 0.07–0.12, *p* < 0.001, I^2^ = 93%) and 0.08 g/cm^2^ at the femoral neck (20 RCTs, 95% CI: 0.05–0.12, *p* < 0.001, I^2^ = 94%), though this result was associated with high heterogeneity, high risk of bias, and very low certainty of evidence.

**Conclusion:**

Our data suggest the possibility that combining HMs with BPs may improve BMD in primary OP more effectively than using BPs alone. However, the results should be interpreted with caution due to the high heterogeneity and low quality of the studies included in the review. Therefore, further well-designed RCTs are needed to confirm these findings.

**Systematic Review Registration:**

https://www.crd.york.ac.uk/prospero/display_record.php?ID=CRD42023392139.

## 1 Introduction

Osteoporosis (OP) is a chronic progressive disorder characterized by a decline in bone mineral density (BMD) and an increase in bone fragility (Raisz, 2005; Seeman and Delmas, 2006). The prevalence rate of OP worldwide is 18.3%, with 23.1% and 11.7% prevalence rates among women and men, respectively (Salari et al., 2021). The 1-year fatality rate of osteoporotic fractures is as high as 36.7% (van Staa et al., 2001; Clynes et al., 2020). Moreover, OP incurs substantial socioeconomic costs; in the United States, the cost of OP is projected to exceed $95 billion by 2040 (Lewiecki et al., 2019). Therefore, there is an urgent need to manage patients with OP to reduce patient suffering and economic burden.

OP can be classified into primary and secondary types, with primary OP further divided into postmenopausal OP (PMOP) and senile OP (Jolly et al., 2018). PMOP occurs in women shortly after menopause due to estrogen deficiency, which activates osteoclast differentiation and increases osteoblast apoptosis. This leads to a rate of bone resorption that exceeds the rate of bone formation (Iseme et al., 2017). On the other hand, senile OP arises from aging-related increases in reactive oxygen species (ROS), which activate osteoclasts. Additionally, decreased renal function leads to reduced vitamin D synthesis, and impaired osteoblast function results in bone formation that cannot keep pace with bone resorption (McClung et al., 2013).

The International Osteoporosis Foundation (IOF) has recommended pharmacologic treatments for OP, including bisphosphonates, selective estrogen receptor modulators (SERMs), hormone replacement therapy (HRT), anabolic agents, and receptor activator of nuclear factors κB ligand (RANKL) inhibitors (Tu et al., 2018). These treatments address OP by either slowing bone resorption or promoting bone formation (Khosla and Hofbauer, 2017). However, SERMs can increase the risk of deep vein thrombosis (Artero et al., 2012), HRT is associated with a heightened risk of breast and uterine cancers (Sjögren et al., 2016; Lupo et al., 2015), anabolic agents like teriparatide can cause hypercalcemia (Minisola et al., 2019), and RANKL inhibitors like denosumab may result in hypocalcemia or an increased risk of infections (Tsvetov et al., 2020).

Among them, the most widely recognized standard treatment for OP is BPs. While BPs effectively inhibit excessive bone resorption, this inhibition can lead to side effects and a decline in bone quality through coupling events (Ensrud and Crandall, 2017; Park and Ko, 2022). This reduction in bone quality is associated with an increased risk of atypical fractures (Tella and Gallagher, 2014; Ensrud and Crandall, 2017). As a result, a “drug holiday” has been considered for patients undergoing BP therapy to reduce these risks (McClung et al., 2013). Nevertheless, it is worth noting that in patients at high risk within the osteoporosis group, drug holiday was associated with an elevated risk of clinical fractures (Camacho et al., 2020). Therefore, there is a need for studies to identify alternatives that can safely and effectively compensate for BPs by increasing BMD in a shorter period.

Herbal medicines (HMs), a rich source of diverse bioactive compounds awaiting discovery (Słupski et al., 2021), have already been reported in various studies for their potential effects on OP (Liu et al., 2014; Lu et al., 2022). In clinical settings across Asia, where HMs are frequently prescribed, case series indicate that the concurrent use of HMs and BPs has sometimes potential benefits over using BPs alone in improving BMD in patients with OP (Yu et al., 2012; Li et al., 2020). Nevertheless, no previous studies have analyzed the overall effect of combined HMs and BPs in patients with primary OP.

Therefore, this study aims to evaluate the effects of concurrent use of HMs and BPs in patients with primary OP, identify which specific HMs and botanical drugs are predominantly utilized, assess the BMD improvement effects of frequently used herbal remedies, and provide relevant research insights.

## 2 Methods

This systematic review and meta-analysis was performed according to the Preferred Reporting Items for Systematic Reviews and Meta-Analyses (PRISMA) and A Measurement Tool to Assess Systematic Reviews (AMSTAR2) guidelines. This review was registered in the Prospective Register of Systematic Reviews (PROSPERO) [registration number CRD42023392139].

### 2.1 Search strategy

We conducted a comprehensive search for randomized controlled trials (RCTs) across 9 databases, including PubMed, Embase, Cochrane Library, China National Knowledge Infrastructure (CNKI), Wanfang, Koreanstudies Information Service System (KISS), Kmbase, Science On, and Oasis, up to 31 August 2023. We identified additional RCTs through a review of the bibliographies of relevant articles and a manual search on Google Scholar. No language restrictions were imposed. The search strategy included the following keywords: osteoporosis, herbs, decoctions, plant extracts, traditional medicine, and Chinese medicine. The combination of keywords was tailored to each database, and the corresponding details are presented in Supplementary Table S1A–S1C.

### 2.2 Study selection and eligibility criteria

Two authors (M-G Kim and Y-S Yoo) separately checked the titles and abstracts of relevant articles and subsequently assessed the full text against the eligibility criteria for final inclusion. Any disagreements regarding the selection of studies were resolved through discussion with a third researcher (E-J Lee). The eligibility criteria applied for study selection are discussed in the following sections.

#### 2.2.1 Participants

We included studies involving patients diagnosed with primary OP, including PMOP and senile OP, regardless of sex. According to the World Health Organization (WHO) diagnostic criteria, only patients with OP with a T-score of −2.5 or lower were considered for inclusion.

#### 2.2.2 Intervention types

In our study, we included research in which the intervention group involved the concurrent treatment of HMs and BPs. There were no restrictions on the types of HMs and BPs. Additionally, there were no constraints on supplements other than HMs and BPs.

#### 2.2.3 Control types

We included studies that used BP administration as the control group without imposing restrictions on the types of BPs or the use of supplements. Additionally, to facilitate the comparison between the combined treatment of HMs *plus* BPs and BPs monotherapy, we only included studies where treatments other than HM were identically used in the control group.

#### 2.2.4 Outcome measures

We included studies that specifically involved BMD values as outcome measures using dual-energy X-ray absorptiometry (DXA) for at least one of the following sites: lumbar spine, femoral neck, or total hip. Additionally, studies that presented only T-scores without BMD values or did not provide post-treatment BMD results were excluded.

#### 2.2.5 Study design

We included only RCTs in this review. Studies using the term 'randomization' without providing details were included. However, quasi-RCTs with inappropriate random sequence generation were excluded. Additionally, RCTs with Jadad scores (Jadad et al., 1996) below two were excluded to ensure quality control in the review.

### 2.3 Data extraction

Two authors (M-G Kim and Y-S Yoo) extracted data using a standardized data collection form (Excel 2007; Microsoft, Redmond, WA, United States), followed by cross-checking. The extracted information included: title, first author, published year, Digital Object Identifier (DOI), setting of study, type of OP, inclusion of fracture, number of arms, number of participants, age of participants, duration of OP, duration of menopause, intervention/control (types of BPs, types of HMs, the botanical drugs constituting HM, type of formula, pattern identification of HMs, dose, and treatment period), site of BMD measurement (e.g., lumbar spine, femoral neck, and total hip), BMD values (mean and standard deviation of baseline and endpoint and evaluation period), bone marker values (mean and standard deviation of baseline and endpoint and evaluation period), statistical significance of the outcome, adverse effects (frequency and type), withdrawal (number and reason), study institution and country of the corresponding research. The type of OP was classified based on the criteria outlined in the referenced RCTs.

In particular, following the Consensus statement on the Phytochemical Characterisation of Medicinal Plant extracts (ConPhyMP) guidelines (Heinrich et al., 2022), data were extracted on the reporting of the pharmaceutical producer, extraction process, quality control reports, and chemical analysis reports for the HMs used in RCTs included in the review. As per the clinical practice guidelines of Korean medicine for OP (Xie et al., 2011), HMs used in this study were classified into three types: kidney yang deficiency pattern, liver-kidney yin deficiency pattern, and syndrome of qi stagnation and blood stasis. In addition, we summarized the botanical drugs used in more than 10 RCTs. However, the courier botanical drugs that harmonize the drug, such as *Ziziphus jujuba Mill* [Rhamnaceae; Jujubae Fructus] and *Glycyrrhiza uralensis Fisch. ex DC* [Fabaceae; Glycyrrhizae Radix et Rhizoma], were not counted as frequently used botanical drugs. Furthermore, all botanical drugs have been presented with their scientific names, including authorities, and their respective families.

Any disagreements were resolved via a discussion until a consensus was reached or by consulting a third author (E-J Lee). If additional information was required, we contacted the corresponding author of the relevant study via email.

### 2.4 Quality assessment

Two authors (M-G Kim and Y-S Yoo) individually assessed the risk of bias in the included studies and evaluated the quality of evidence for each key finding. Any discrepancies between the two authors were resolved through discussion with a third author (E-J Lee) until a consensus was reached.

The risk of bias in the included studies was assessed using Cochrane’s Risk of Bias 2 (RoB2) tool, evaluating five different domains: randomization process, deviations from intended interventions, missing outcome data, measurement of the outcome, and selection of the reported result. Each domain was categorized into one of three groups: “low risk,” “some concern,” or “high risk.” The overall risk of bias assessment for each study was determined as follows: if all domains were classified as low risk, the overall risk was considered low; if there were some concerns in one or more domains, the overall risk was categorized as some concerns; and if any domain was classified as high risk, the overall risk was assessed as high risk. To evaluate publication bias, both graphical presentations (Funnel plot) and statistical tests (Egger’s regression test) were carried out by the “meta” package (by Guido Schwarzer, R Foundation for Statistical Computing, Vienna, Austria) in R version 4.3.2. When potential publication bias was suspected by Egger’s test, we corrected the effect size using the trim-and-fill method.

To assess the quality of evidence for the meta-analysis results, the Grading of Recommendations Assessment, Development, and Evaluation (GRADE) approach was employed. Using the online GRADEpro Guideline Development Tool (https://gdt.gradepro.org/), we evaluated the certainty of evidence for meta-analytic results including three or more RCTs as high, moderate, low, or very low. This assessment took into consideration various factors, including study design, risk of bias, inconsistency, indirectness, imprecision, publication bias, effect size, and potential confounding.

### 2.5 Statistical and meta-analysis

To present the mean and standard deviation (SD) of age, treatment duration, OP and menopausal periods, and BMD, we utilized the weighted mean and SD functions, considering the sample size. In cases where data such as age, OP duration, menopausal period, and BMD values were not available, we contacted the corresponding authors of the relevant studies via email to obtain the necessary information. In instances where this was not possible, we synthesized the remaining data excluding missing values. We analyzed the *p*-values of mean age, initial BMD, treatment duration, and duration of OP and confidence intervals (CI) of the BMD change rate. Statistical significance was set at *p* < 0.05, and the CI was set at 95%.

We conducted a meta-analysis using Review Manager software version 5.4.1 (Cochrane, London, United Kingdom) to determine if combining HM with BP is more effective compared to BP alone in improving BMD and bone markers in patients with primary OP. Meta-analyses were performed for studies using the same interventions, comparisons, and outcome measurements. Studies incorporating a combination of calcitonin and estrogen, which can considerably affect BMD values, as well as those with disparate treatment and assessment durations, were excluded from the meta-analysis.

When the necessary data were accessible, subgroup analyses were conducted based on OP types (PMOP, Senile OP), treatment duration (3, 6, and 12 months), and frequently used HM to inspect heterogeneity or evaluate treatment effects among subgroups. BMD with the same unit of measurement was pooled using mean difference (MD) with a 95% CI, while bone markers with different units were pooled using standardized mean difference (SMD). Heterogeneity among studies was assessed using the chi-squared (χ^2^) test and the I-squared (I^2^) statistic. We had planned to use a random-effects model for high heterogeneity (I^2^ ≥ 50%) and apply a fixed-effects model for cases of low heterogeneity. However, due to the diverse HMs used in the studies included in our review, leading to potential heterogeneity, we applied a random-effects model throughout the analysis. Furthermore, to confirm the robustness of the meta-analysis results, sensitivity analyses were conducted by iteratively excluding one study at a time or by excluding studies by the group from each meta-analysis.

## 3 Results

### 3.1 Basic characteristics of included RCTs

**FIGURE 1:**
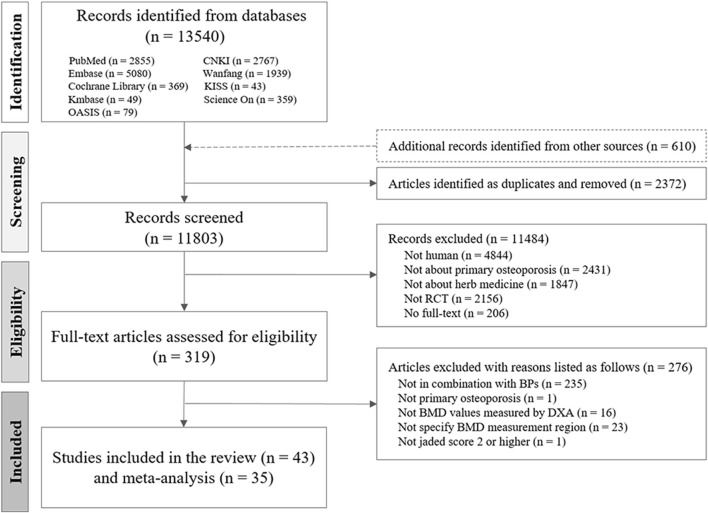
Flow chart of the study selection process. CNKI, China National Knowledge Infrastructure; RCT, randomized controlled trial; HM, herbal medicine; BMD, bone mineral density; OP, osteoporosis; BP, bisphosphonate.

Of the 13,540 studies identified during the initial screening, 43 met the eligibility criteria (Figure 1). In total, 4,470 individuals (1,261 males and 3,209 females) participated in the study, including 1,442 participants with PMOP from 15 RCTs and 3,028 participants with senile OP from 28 RCTs. Among the included participants, 188 in two RCTs on PMOP and 409 in five RCTs on senile OP experienced fractures.

The mean age of the participants was 65.8 ± 6.6 years, the average duration of OP was 4.5 ± 2.7 years, the average duration of menopause was 7.9 ± 2.3 years, and the average treatment period was 5.3 ± 2.6 months. The mean initial BMD of participants was 0.721 ± 0.066 g/cm^2^ at the lumbar spine, 0.646 ± 0.068 g/cm^2^ at the femoral neck, and 0.625 ± 0.098 g/cm^2^ at the total hip. In each RCT, there were no significant differences in the basic characteristics between the intervention and control groups (Table 1).

**TABLE 1:** Characteristics of randomized controlled trials included in the review.

Items	PMOP	Senile OP	Total
N. of RCTs	15	28	43
N. of participants (mean) Male/Female	1,442 (96.1 ± 27.2) 0/1,442	3,028 (108.1 ± 47.6) 1,261/1,767	4,470 (104.0 ± 41.7) 1,261/3,209
N. of participants with fracture (N. of RCTs)	188 (2)	409 (5)	597 (7)
Intervention type			
HM *plus* ALE versus ALE	13	25	38
HM *plus* ZOL versus ZOL	2	3	5
Mean age of participants (year)	59.9 ± 4.6	68.7 ± 5.3	65.8 ± 6.6
Mean of initial BMD (N. of RCTs^a^)			
Lumbar spine	0.722 ± 0.060 (14)	0.720 ± 0.068 (26)	0.721 ± 0.066 (40)
Femoral neck^*^	0.609 ± 0.075 (10)	0.673 ± 0.047 (16)	0.646 ± 0.068 (26)
Total hip^*^	0.505 ± 0.005 (1)	0.705 ± 0.005 (1)	0.625 ± 0.098 (2)
Treatment period (month)	5.1 ± 2.1	5.4 ± 2.8	5.3 ± 2.6
Duration of osteoporosis (year)	3.5 ± 3.6	5.0 ± 2.1	4.5 ± 2.7
Duration of menopause (year)	7.9 ± 2.3	none	7.9 ± 2.3
N. of HM types	13	18	28
Bone markers^b^			
P1NP	4	3	7
Osteocalcin	9	10	19
Alkaline Phosphate	2	8	11
Bone specific alkaline phosphate	6	4	10
PGC-1α	2	0	2
CTX	6	6	12
TRACP	3	2	5
Calcium (Urine Calcium)	0 (1)	7 (2)	7 (3)
Phosphorus	0	7	7
25(OH)_2_D_3_	0	2	2
Estradiol	4	0	4
Parathyroid hormone	1	2	3

PMOP, postmenopausal osteoporosis; OP, osteoporosis; N., number; HM, herbal medicine; ALE, alendronate; ZOL, zoledronate; BMD, bone mineral density; RCT, randomized controlled trial; P1NP, procollagen-1 N-terminal peptide; PGC-1α, Peroxisome proliferator-activated receptor gamma coactivator 1-alpha; CTX, C-telopeptide of type 1 collagen; TRACP, Tartrate-resistant acid phosphatase; 25 ${\left(OH\right)}_{2}D_{3}$ , 25-hydroxivitaminD_3_.

^a^ The count of each BMD measurement site.

^b^ Only bone markers measured in two or more RCTs, were counted.

*p < 0.05 compared between PMOP group and senile OP group.

### 3.2 Characteristics of intervention and control

The control groups received BPs without restrictions on supplements (vitamin D or calcium), and the intervention groups received HMs in addition to the BPs received by the control groups. In 38 RCTs (4,052 participants), the control groups received alendronate (ALE), and in five RCTs (418 participants), the control groups received zoledronate (ZOL). Ten RCTs used ALE or ZOL alone, and the remaining 33 RCTs used ALE or ZOL in combination with supplements (calcium or vitamin D in 27 RCTs, calcitonin in 5 RCTs, and estrogen in one RCT) (Tables 1 and 2).

**TABLE 2:** Summary of included RCTs.

Study (year)	Fx	Age (year)	Sample size (I/C)	Intervention	Control^a^	Period (month)	BMD	Bone markers	Jadad Score
1. HM + ALE versus ALE									
*Postmenopausal osteoporosis (12 RCTs)*									
Chen (2019)	O	69.1 ± 11.7	108 (57/51)	Gushukang capsule + C	ALE Vit D3 (70/w) Calcium. D (1,500)	6	L^b,c^	ALP^b,c^, BGP^b,c^, P1NP^b,c,^ CTX^b,c^	2
Chen et al. (2021)	uk	57.1 ± 0.5	87 (44/43)	Modified Erxian decoction + C	ALE (10/w) Estrogen (1/m) Calci D (600)	6	L^b,c^ F^b,c^ H^b,c^	BALP^b,c^, BGP^b,c^, CTX^b,c^, TRACP^b,c^, PTH^b,c^	2
Ju et al. (2020)	uk	60.8 ± 0.4	96 (48/48)	Erxian decoction + C	ALE (10) Calcium. D (1,000)	6	L^b,c^ F^b,c^	BALP^b,c^, BGP^b,c^, P1NP^b,c,^ CTX^b,c^, TRACP^b,c^	2
Liu and Wang (2016)	uk	56.0 ± 6.8	124 (62/62)	Erxian Bushen decoction + C	ALE (70/w) Calcium. (600) Vit D (125IU)	6	L^b,c^ F^b,c^	OPN^b,c^, PGC-1α^b,c^, ERRα^b,c^, SRC-3^b,c^	2
Liu et al. (2019)	uk	57.3 ± 4.5	116 (58/58)	Bushen Juanbi decoction + C	ALE (10) Calcium. D (600)	3	L^b,c^ F^b,c^	BALP^b,c^, BGP^b,c^, P1NP^b,c^, SOD^b,c^ T-AOC^b,c^, AOPP^b,c^, CTX^b,c^, MAOA^b,c^	2
Lu et al. (2016)	uk	64.1 ± 6.0	120 (56/55)	Kanggu zengsheng jiaonang capsule + C	ALE (70/w) Calcium. (3,000)	6	L^b,c^ F^b,c^	ALP^b,c^, BGP^b,c^, P1NP^b,c^, CTX^b,c^	2
Xu et al. (2009)	uk	58.2 ± 2.8	104 (52/52)	Xianlinggubao capsule + C	ALE (70/w)	6	L^b,c^		2
Xu et al. (2010)	uk	60.2 ± 9.7	80 (40/40)	Qianggu capsule + C	ALE (70/w)	6	L^b,c^ F^b^		2
Xu et al. (2019)	uk	54.6 ± 3.1	86 (43/43)	Wenshen Gushu decoction + C	ALE (70/w) Calcium. D (1,000)	2	F^b,c^	PGC-1α^b,c^, Bcl-2^b,c^, SRC-3^b,c^	2
Xue et al. (2018)	uk	60.7 ± 6.1	108 (54/54)	Bushen Zhuanggu decoction + C	ALE (70) Calcium. D (1,200)	3	L^b,c^ F^b,c^	BALP^b,c^, BGP^b,c^, TRACP^b,c^, E2^b,c^	2
Yang et al. (2017)	O	53.3 ± 7.8	80 (40/40)	Duhuo Jisheng decoction + C	ALE (10) Calcium (1,200) Calcitriol (0.25 μg)	3	L^b,c^ F^b,c^	BGP^b,c^, CTX^b,c^	2
Yang (2021)	uk	62.7 ± 4.4	95 (48/47)	Kuntai capsule + C	ALE (70/w) Calcium. (1,000) Vit D (uk)	3	L^b,c^ F^b,c^	E2^b,c^, FSH^b,c^, LH^b,c^	2
Zhu et al. (2020)	uk	54.9 ± 3.1	46 (23/23)	Yangxue Gushen decoction + C	ALE (10)	3	L^b,c^	ALP^b,c^, BALP^b,c^, BGP^b,c^, U-C^b,c^	2
*Senile osteoporosis (24 RCTs)*									
Bao and Ling (2015)	uk	58.8 ± 1.0	140 (70/70)	Xianlinggubao capsule + C	ALE (70) Calcium. D (600) Vit D (125IU)	6	L^b,c^	uk^b,c^	2
Chen et al. (2015)	uk	71.9 ± 10.8	118 (59/59)	Xianlinggubao capsule + C	ALE (60/w) Calcitonin (50U/3d)	3	L^b,c^ F	ALP^b,c^, BGP^b,c^ Ca, P, U-Ca^b,c^	2
Chu et al. (2021)	uk	63.0 ± 5.5	108 (54/54)	Astragalus L&K Tonifying decoction + C	ALE (10)	1	L^b,c^	IL-6^b,c^, TNF-α^b,c^	2
Cui et al. (2020)	uk	74.4 ± 5.2	60 (30/30)	Gukangfang granule + C	ALE (70) Alfacalcidol (0.0005) Calci D (600)	3	L^b,c^		2
Du and Zhao (2018)	O	63.0 ± 5.6	60 (30/30)	Xianlinggubao capsule + C	ALE (70/w) Calcitriol (50 μg) Calcium. D (600)	6	L^b,c^ F^b,c^		2
Fu et al. (2016)	uk	74.1 ± 15.9	300 (150/150)	Xianlinggubao capsule + C	ALE (70) Calcium. D3 (600)	6	L^b,c^	ALP^b,c^, Ca^b,c^, P^c^	2
Gui et al. (2017)	uk	66.2 ± 8.3	60 (30/30)	Bushen Yiqi Huayu decoction + C	ALE (70/w) Calcium. D (600)	6	L^b,c^		2
Guo and Yuan (2020)	uk	70.4 ± 5.1	84 (42/42)	Xianlinggubao capsule + C	ALE (10) Calcitonin (0.0083)	3	L^c^ F^c^	ALP^c^, Ca^c^, P^c^	2
Huang et al. (2018)	X	uk	176 (88/88)	Bushentang + C	ALE (70/w)	6	L^b,c^ F^b,c^	ALT, BUN, Ca, CREA, P, UA	2
Jiang et al. (2020)	uk	71.2 ± 8.5	126 (62/64)	Lujiao Zhuanggu capsule + C	ALE (uk) Calcitriol (uk) Calcium. D (uk)	6	L^c^ F^c^	BALP^c^, BGP^c^, TRACP^c^, Ca^c^, P^c^, Calcitonin^c^, PTH^c^	2
Kang et al. (2020)	uk	uk	64 (32/32)	Xianlinggubao capsule + C	ALE (10) Calcitonin (0.0083)	3	L^b,c^ F^b,c^	ALP^b,c^, BGP^b,c^	2
Li et al. (2019)	uk	75.5 ± 3.5	124 (62/62)	Xianlinggubao capsule + C	ALE (70/w) Calcium. D (600) Vit D (125IU)	6	L^b,c^	ALP^b,c^, P^b,c^	2
Li et al. (2021)	O	76.1 ± 6.1	64 (31/32)	Biqi capsule + C	ALE (70/w) Calcium. D (300)	4	F^b,c^	hs-CRP^c^	2
Shen et al. (2017)	uk	72.8 ± 8.2	145 (73/72)	Bushen Huoxue decoction + C	ALE (70) Calcium. D (600)	3	L^b,c^	BGP^b,c^, P1NP^b,c^, CTX^b,c^	3
Tu et al. (2017)	uk	62.0 ± 5.2	116 (58/58)	Compound Epimedium decoction + C	ALE (70/w) Calcium. D (1,500)	12	L^b,c^ F^b,c^	ALP^b,c^, BALP^b,c^, BGP^b,c^, P1NP^b,c^, CTX^b,c^	2
Wang et al. (2018)	uk	71.8 ± 9.2	80 (40/40)	Guli Jiaonang capsule + C	ALE Vit. D3 (70/w)	6	L^b,c^	BGP^b,c^, CTX^b,c^, 25 ${\left(OH\right)}_{2}D_{3}$ ^b,c^	2
Wang (2020)	uk	73.2 ± 2.4	130 (65/65)	Tonifying Q&K decoction + C	ALE (10) Calci D (1,200)	6	L^b,c^ H^b,c^		2
Xie et al. (2015)	O	60.7 ± 10.1	94 (48/48)	Duhuo Jisheng decoction + C	ALE (10) Calcitonin (50U/2d) Calcium. (500) Vit D (200IU)	4	F^b,c^		2
Yan et al. (2017)	uk	73.2 ± 5.5	104 (52/52)	Increased densities decoction + C	ALE (70/w)	3	L^b,c^ F^b,c^		2
Zeng and He (2015)	uk	74.4 ± 6.0	79 (40/39)	Xianlinggubao capsule + C	ALE (70/w) Calcitonin (50U/t)	12	L^b,c^	ALP^b,c^, BGP^b,c^ Ca^b,c^, P^b,c^	2
Zhang and Shang (2014)	uk	68.1 ± 9.2	90 (45/45)	Xianlinggubao capsule + C	ALE (70/w)	6	L^b,c^	ALP^b,c^ Ca, U-Ca^b,c^	2
Zhang (2019)	uk	62.4 ± 1.3	70 (35/35)	Yishen Zhuanggu decoction + C	ALE (70/w) Calcium. (1,000) Vit D (400IU)	6	L^b,c^ F^b,c^		2
Zhang et al. (2021)	uk	62.7 ± 6.1	102 (51/51)	uk decoction + C	ALE (10/d)	3	L^b,c^	BGP^b,c^, P1NP^b,c^, CTX^b,c^, 25 ${\left(OH\right)}_{2}D_{3}$	2
Zhao et al. (2020)	uk	70.7 ± 2.5	112 (56/56)	Xianlinggubao capsule + C	ALE (70/w)	2	L^c^ F^c^		2
Zhen et al. (2014)	uk	66.7 ± 8.5	100 (50/50)	Gubi decoction + C	ALE (70/w) Calcium. D (600)	6	L^b,c^ F^b,c^		2
2. HM + ZOL+α versus ZOL+α									
*Postmenopausal osteoporosis (2 RCTs)*									
Xiang et al. (2013)	uk	57.6	60 (30/30)	Duhuo Jisheng decoction + C	ZOL (4/y) Calcium. (1,000)	12	L^b^	BALP, BGP^b,c^ E2^b,c^	2
Zhang et al. (2015)	uk	64.1 ± 5.3	77 (37/40)	Liuwei Dihuang pill + C	ZOL (5/6m) Calcium. D (uk)	6	L^b,c^	E2^b,c^	2
*Senile osteoporosis (3 RCTs)*									
Bu et al. (2021)	O	70.3 ± 2.2	80 (40/40)	Bushen Huoxue decoction + C	ZOL (5) Calcitriol (0.0004) Calcium. (1,000) Vit D (400IU)	6	L^b,c^ F^b,c^	BALP^b,c^, BGP^b,c^, CTX^b,c^, NTX-1^b,c^	2
Feng et al. (2018)	uk	62.1 ± 7.1	120 (60/60)	Tenghuang Jiangu capsule + C	ZOL (5/y)	12	L^b,c^ F^b,c^	MMP-13^b,c^, M/T^b,c^, TIMP-1^b,c^	2
Hu et al. (2020)	O	63.9 ± 3.0	78 (39/39)	Modified Bushen Huoxue decoction + C	ZOL (5/m) Calcitriol (0.0004) Calcium. (600) Vit D (120IU)	1	L^b,c^ F^b,c^	BALP^b,c^, BGP^b,c^, PCT^b,c^, TGF-β1^b,c^, VEGF^b,c^, CTX^b,c^, SICAM-1^b,c^, TRACP^b,c^, PTH^b,c^	2

^a^ Only drug dosage units other than ‘mg/d’ are shown in the table.

^b^ *p* < 0.05 compared within the intervention group before and after treatment.

^c^ *p* < 0.05 compared between intervention and control group after treatment.

Fx, fracture; I, intervention group; C, control group; ALE, alendronate; ZOL, zoledronate; uk, unknown; L&K, liver and kidney; Q&K, qi and kidney; w, week; m, month; y, year; t, time; Vit, vitamin; Calcium., calcium carbonate; L, lumbar spine; F, femoral neck; H, total hip; ALP, alkaline phosphatase; AOPP, advanced oxidized protein products; BALP, bone alkaline phosphatase; Bcl-2, B-cell leukemia/lymphoma 2; BGP, bone gla-protein; BUN, blood urea nitrogen; Ca, Calcium; CREA, creatinine; CTX, cross-linked C-telopeptide of type I collagen; E2, estradiol; ERR, Estrogen-related receptor alpha; FSH, follicle stimulation estrogen; hs-CRP, high sensitive C-reactive protein; IL-1, interleukin-1; IL-6, interleukin-6; LH, luteinizing hormone; MAOA, monamine oxidase A; MMP-13, Matrix metalloproteinases 13; M, MMP-13; NTX-1, N-terminal telopeptide of type 1 collagen; OPN, osteopontin; P, phosphorus; P1NP, procollagen Ⅰ N-terminal peptide; PCT, procalcitonin; PGC-1α, Peroxisome proliferator-activated receptor gamma coactivator 1-alpha; PTH, para-thyroid hormone; SICAM-1, soluble intracellular adhesion molecule 1; SOD, super oxide dismutase; SRC-3, Steroid Receptor Coabtivator-3; T-AOC, total antioxidant capacity; TGF-β1, Transforming growth factor beta 1; TIMP-1, tissue inhibitor of metalloproteinase 1; T, TIMP-1; TNF-α, tumor necrosis factor alpha; TRACP, tartrate resistant acid phosphatase; UA, uric acid; U-Ca, urine Calcium; VEGF, vascular endothelial growth factor; 25 ${\left(OH\right)}_{2}D_{3},$ 25-hydroxyvitamin D3.

The most frequently used HM was Xianlinggubao capsule (XLGB) in 11 RCTs, followed by Bushen Huoxue (three RCTs), Duhuo Jisheng (three RCTs), and Erxian (two RCTs) decoctions. HMs were prescribed mainly by applying three pattern identifications: kidney yang deficiency (26 RCTs), syndrome of qi stagnation and blood stasis (six RCTs), and liver-kidney yin deficiency (two RCTs) (Table 1; Supplementary Table S2). Seventy botanical drugs were used in this review, 11 of which were used at least 10 times. The botanical drugs used frequently included *Epimedium koreanum Nakai* [Berberidaceae; Epimedii Herba] (27 RCTs), *Rehmannia glutinosa (Gaertn.) DC* [Orobanchaceae; Rehmanniae Radix Recens] (21 RCTs), *Cullen corylifolium (L.) Medik* [Fabaceae; Psoraleae Fructus] (18 RCTs), and *Angelica sinensis (Oliv.) Diels* [Apiaceae; Angelicae Sinensis Radix] (17 RCTs) (Table 3). Detailed information on the HMs, including composition, dosage, pharmaceutical producer, extraction process, quality control reports, and chemical analysis reports, is provided in Supplementary Table S2.

**TABLE 3:** Botanical drugs used frequently in HMs.

Frequency	Botanical drugs	Bioactive compounds
27	*Epimedium koreanum Nakai* [Berberidaceae; Epimedii H.]	Icariin, Epimedin B, Epimedin C
21	*Rehmannia glutinosa (Gaertn.) DC.* [Orobanchaceae; Rehmanniae R. Recens]	Catalpol
18	*Cullen corylifolium (L.) Medik.* [Fabaceae; Psoraleae F.]	Corylin, Bavachin, Bavachalcone, Bakuchiol
17	*Angelica sinensis (Oliv.) Diels* [Apiaceae; Angelicae Sinensis R.]	Ligustilide
15	*Drynaria fortunei (Kunze) J. Sm.* [Polypodiaceae; Drynariae Rh.]	Naringin
15	*Salvia miltiorrhiza Bunge* [Lamiaceae; Salviae Miltiorrhizae R. et Rh.]	Tanshinones, Phenolics
15	*Eucommia ulmoides Oliv.* [Eucommiaceae; Eucommiae C.]	Quercetin, Kaempferol, β -carotene
11	*Achyranthes bidentata Blume* [Amaranthaceae; Achyranthis Bidentatae R.]	Berberine, Baicalein, Quercetin, Rutin
11	*Astragalus membranaceus (Fisch.) Bunge* [Fabaceae; Astragali R.]	Astragalus polysaccharide, Astragaloside Ⅳ
11	*Lycium chinense Mill.* [Solanaceae; Lycii F.]	Lycium barbarum polysaccharides, Carotenoids, Kaempferol, Quercetin, Myriceti
10	*Dipsacus asper Wall. ex Henry* [Dipsacaceae; Dipsaci R.]	β -sitosterol, Gentisin, Sitogluside, Ursolic acid

HM, herbal medicine; H., Herba; R., Radix; F., Fructus; Rh., Rhizoma; C., Cortex.

In total, 28 types of HMs each consisting of an average of seven botanical drugs prepared using traditional methods, were prescribed in the RCTs included in our review. Among these, 17 were traditional decoctions, while 11 were modernized formulations, including 9 capsules, 1 granule, and 1 pill. Of the modernized formulations, 7 types of HMs with active compounds reported in prior studies are specified, and the bone metabolism-related functions of each compound are detailed in Supplementary Table S3.

### 3.3 Characteristics of outcome measurement

All 43 RCTs established BMD as the primary outcome measured using DXA, and BMD was measured at the lumbar spine (40 RCTs), femoral neck (26 RCTs), and total hip (two RCTs). A total of 34 types of bone markers were used as secondary outcomes in 32 RCTs. Among these RCTs, the following five bone formation markers were employed in two or more studies: procollagen-1 N-terminal peptide (P1NP) in seven RCTs, osteocalcin (OC) in 19 RCTs, alkaline phosphate (ALP) in 11 RCTs, bone-specific alkaline phosphate (BALP) in 10 RCTs, and peroxisome proliferator-activated receptor gamma coactivator 1-alpha (PGC-1α) in two RCTs. Additionally, two bone resorption markers, C-telopeptide of type 1 collagen (CTX) in 12 RCTs and tartrate-resistant acid phosphatase (TRACP) in five RCTs, were used, along with six mineral/hormone indicators: calcium (Ca) in seven RCTs, urine calcium (U-Ca) in three RCTs, phosphorus (P) in seven RCTs, 25-hydroxyvitamin D_3_ (25(OH)_2_D_3_) in two RCTs, estradiol in four RCTs, and parathyroid hormone (PTH) in three RCTs (Table 1).

### 3.4 Combined effect of HMs *plus* BPs

A meta-analysis was conducted on 35 out of the total 43 RCTs, excluding eight RCTs. Exclusions encompassed six RCTs (Chen et al., 2015; Xie et al., 2015; Zeng and He, 2015; Guo and Yuan, 2020; Kang et al., 2020; Chen et al., 2021) that included the use of calcitonin and estrogen and two RCTs (Shen et al., 2017; Yang, 2021) that included disparate treatment and evaluation periods.

#### 3.4.1 HMs *plus* BPs versus BPs

In total, 35 RCTs (HM *plus* ALE versus ALE in 30 RCTs and HM *plus* ZOL versus ZOL in 5 RCTs) were included in the meta-analysis to investigate the BMD improvement effect of HM *plus* BPs compared with that of BPs alone.

**FIGURE 2:**
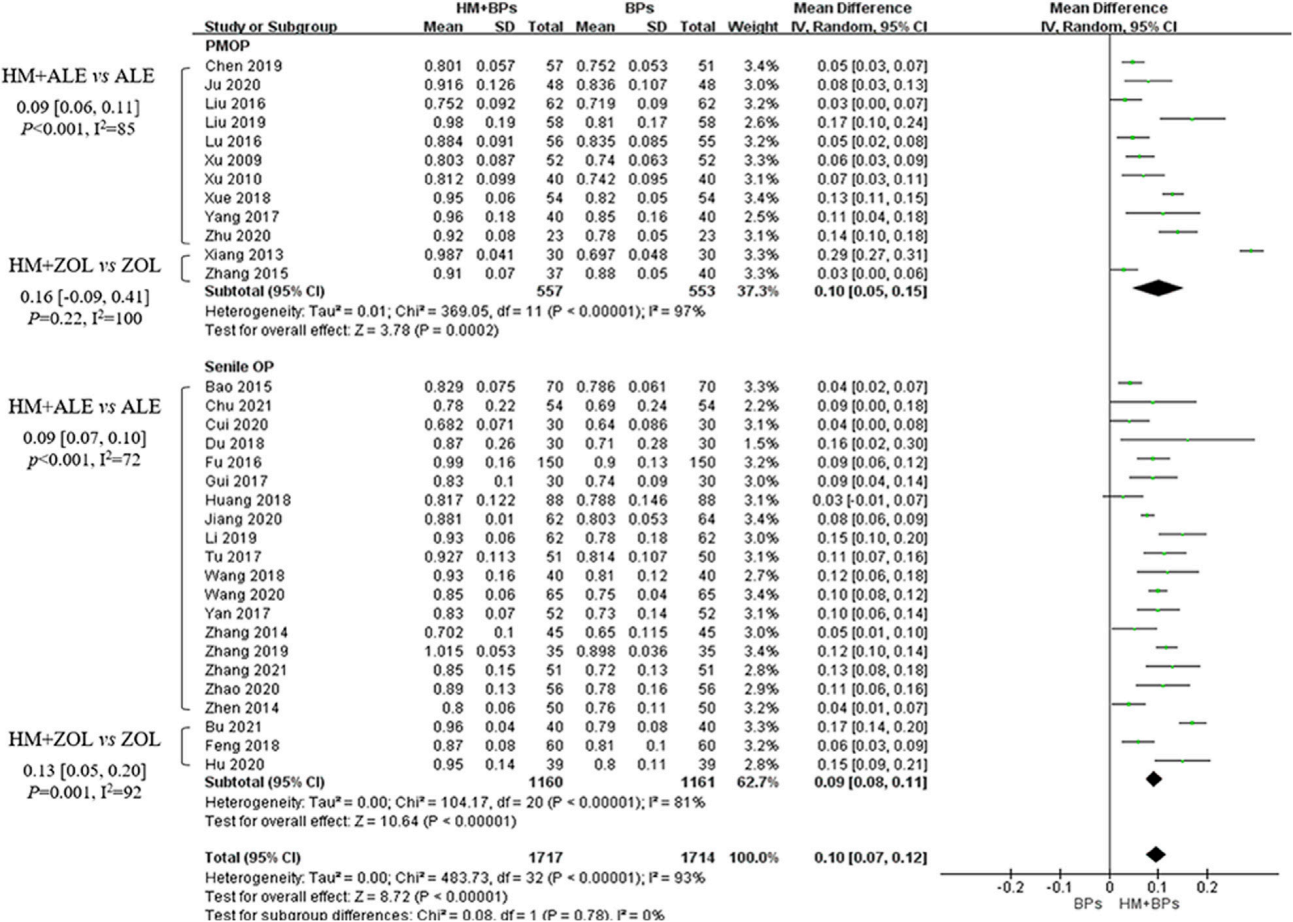
BMD improvement effects of the combined therapy of HM and BPs at the lumbar spine. BMD, bone mineral density; BP, bisphosphonate; HM, herbal medicine; ALE, alendronate; ZOL, zoledronate; PMOP, postmenopausal osteoporosis; SD, standard deviation; CI, confidence interval.

**FIGURE 3:**
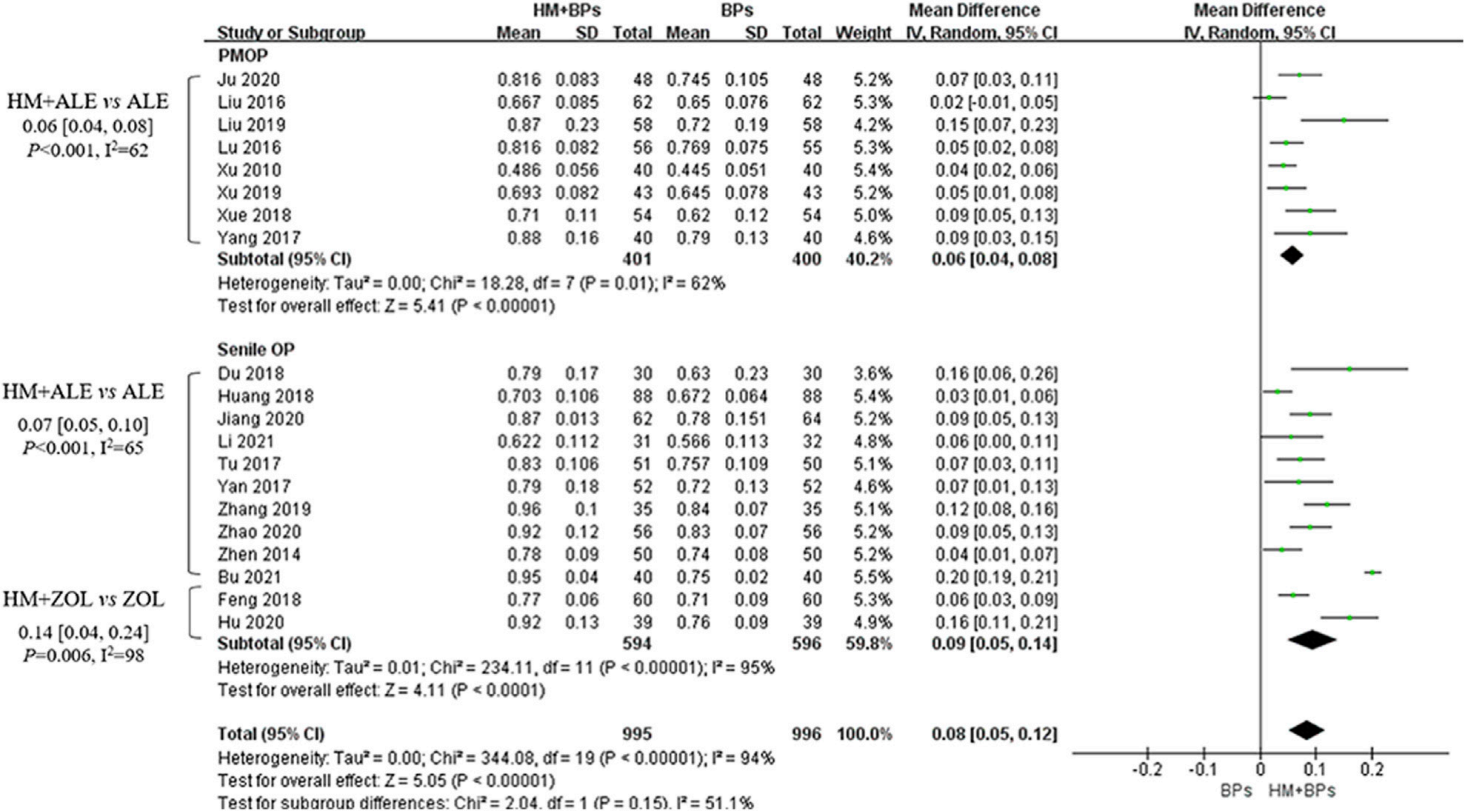
BMD improvement effects of the combined therapy of HM and BPs at the femoral neck. BMD, bone mineral density; BP, bisphosphonate; HM, herbal medicine; ALE, alendronate; ZOL, zoledronate; PMOP, postmenopausal osteoporosis; SD, standard deviation; CI, confidence interval.

Compared to the BPs alone group, the HM *plus* BPs group showed improved BMD score by 0.10 g/cm^2^ (33 RCTs, 95% CI: 0.07–0.12, *p* < 0.001, I^2^ = 93%) at the lumbar spine over 5.6 months and by 0.08 g/cm^2^ (20 RCTs, 95% CI: 0.05–0.12, *p* < 0.001, I^2^ = 94%) at the femoral neck over 5.4 months (Figures 2, 3). In the subgroup analysis according to OP type, there was no statistically significant difference in the improvement of BMD between the PMOP and Senile OP types (Figures 2, 3).

**FIGURE 4:**
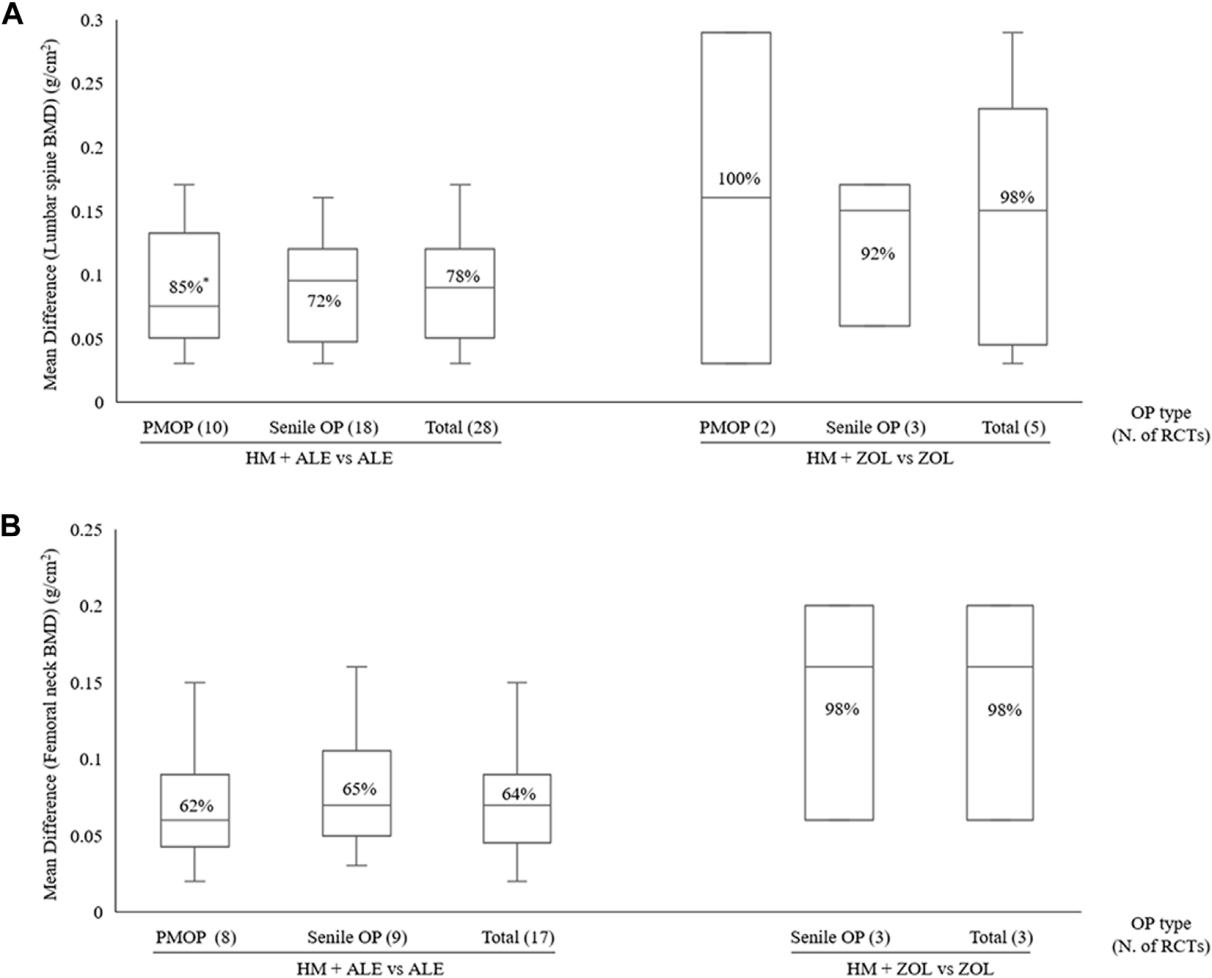
BMD improvement effects according to OP type with combined HM and BPs therapy **(A)** Lumbar spine **(B)** Femoral neck. BMD, bone mineral density; BP, bisphosphonate; HM, herbal medicine; M, month; PMOP, postmenopausal osteoporosis; OP, osteoporosis; N, number; RCT; randomized controlled trial; ^*^, I^2^.

In the subgroup analysis based on the types of concomitant BPs, BMD improvement in the HMs *plus* ZOL group was approximately 1.5–2 times higher than that in the HMs *plus* ALE group (Figures 2–4; Supplementary Figures S1–S3). However, the number of studies investigating the combination of HMs *plus* ZOL was relatively small compared to those examining combinations with ALE, and heterogeneity was higher. In sensitivity analysis, heterogeneity decreased upon excluding RCTs involving HMs *plus* ZOL. Specifically, in the analysis of lumbar and femoral neck BMD in senile OP, excluding RCTs on HMs *plus* ZOL resulted in an upward shift in the certainty of evidence by one grade to ‘Low’ due to decreased heterogeneity (Table 4; Supplementary Table S4).

**TABLE 4:** Characteristics of 35 randomized controlled trials included in the meta-analysis and GRADE evidence profiles.

Subgroup (OP type)		Treat period (month)	N. Of subjects (N. of RCTs)	I/C	Initial BMD (g/cm^2^)	Certainty assessment						Absolute effect (95% CI)	Certainty
						Study design	Risk of bias^a^	Inconsistency^b^	Indirectness	Imprecision	Publication bias		
HMs *plus* BPs at lumbar (Total)		5.6	3,510 (33)	I	0.713 ± 0.064	High	Serious	Very serious	Not serious	Not serious	Undetected	higher MD 0.10 (0.07–0.12)	⊕○○○ Very low
				C	0.715 ± 0.067								
HMs *plus* BPs at lumbar (PMOP)		5.4	1,174 (12)	I	0.706 ± 0.057	High	Serious	Very serious	Not serious	Not serious	Undetected	higher MD 0.10 (0.05–0.15)	⊕○○○ Very low
				C	0.713 ± 0.052								
HMs *plus* BPs at lumbar (Senile OP)		5.7	2,336 (21)	I	0.716 ± 0.067	High	Serious	Very serious	Not serious	Not serious	Undetected	higher MD 0.09 (0.08–0.11)	⊕○○○ Very low
				C	0.716 ± 0.073								
HMs *plus* BPs at femur (Total)		5.4	2,049 (20)	I	0.634 ± 0.073	High	Serious	Very Serious	Not serious	Not serious	Undetected	higher MD 0.08 (0.05–0.12)	⊕○○○ Very low
				C	0.635 ± 0.069								
HMs *plus* BPs at femur (PMOP)		4.4	810 (8)	I	0.594 ± 0.081	High	Serious	Serious	Not serious	Not serious	Strongly suspected^c^	higher MD 0.06 (0.04–0.08)	⊕○○○ Very low
				C	0.597 ± 0.072								
HMs *plus* BPs at femur (Senile OP)		6.1	1,239 (12)	I	0.666 ± 0.045	High	Serious	Very serious	Not serious	Not serious	Undetected	higher MD 0.09 (0.05–0.14)	⊕○○○ Very low
				C	0.666 ± 0.048								
HMs *plus* ALE at lumbar (Total)		5.3	3,092 (28)	I	0.716 ± 0.063	High	Serious	Very serious	Not serious	Not serious	Undetected	higher MD 0.09 (0.07–0.10)	⊕○○○ Very low
				C	0.715 ± 0.069								
HMs *plus* ALE at lumbar (PMOP)		3	350 (4)	I	0.697 ± 0.061	High	Serious	Not serious	Not serious	Not serious	Undetected	higher MD 0.13 (0.12–0.15)	⊕⊕⊕○ Moderate
				C	0.694 ± 0.065								
		6	684 (6)	I	0.722 ± 0.021	High	Serious	Not serious	Not serious	Not serious	Undetected	higher MD 0.05 (0.04–0.07)	⊕⊕⊕○ Moderate
				C	0.725 ± 0.020								
HMs *plus* ALE at lumbar (Senile OP)		<3	486 (5)	I	0.657 ± 0.058	High	Serious	Serious	Not serious	Not serious	Undetected	higher MD 0.09 (0.06–0.13)	⊕⊕○○ Low
				C	0.644 ± 0.056								
		6	1,456 (12)	I	0.734 ± 0.066	High	Serious	Very serious	Not serious	Not serious	Undetected	higher MD 0.08 (0.06–0.10)	⊕○○○ Very low
				C	0.737 ± 0.071								
HMs *plus* ALE at femur (Total)		5.1	1,771 (17)	I	0.634 ± 0.080	High	Serious	Serious	Not serious	Not serious	Strongly suspected^c^	higher MD 0.07 (0.05–0.08)	⊕○○○ Very low
				C	0.634 ± 0.075								
HMs *plus* ALE at femur (PMOP)		<3	390 (4)	I	0.586 ± 0.063	High	Serious	Serious	Not serious	Not serious	Undetected	higher MD 0.09 (0.05–0.12)	⊕⊕○○ Low
				C	0.590 ± 0.050								
		6	420 (4)	I	0.602 ± 0.095	High	Serious	Not serious	Not serious	Not serious	Undetected	higher MD 0.04 (0.02–0.06)	⊕⊕⊕○ Moderate
				C	0.603 ± 0.088								
HMs *plus* ALE at femur (Senile OP)		<3	216 (2)	I	0.705 ± 0.005	High	Serious	Not serious	Not serious	Not serious	Undetected	higher MD 0.08 (0.05–0.12)	⊕⊕⊕○ Moderate
				C	0.715 ± 0.005								
		<6	629 (6)	I	0.671 ± 0.059	High	Serious	Very serious	Not serious	Not serious	Undetected	higher MD 0.07 (0.04–0.11)	⊕○○○ Very low
				C	0.658 ± 0.063								
XLGB *plus* ALE at lumbar		5.5	982 (7)	I	0.742 ± 0.076	High	Serious	Serious	Not serious	Not serious	Undetected	higher MD 0.08 (0.05–0.11)	⊕⊕○○ Low
				C	0.742 ± 0.085								
XLGB *plus* ALE at femur		3.4	172 (2)	I	0.658 ± 0.072	High	Serious	Not serious	Not serious	Not serious	Undetected	higher MD 0.11 (0.05–0.17)	⊕⊕⊕○ Moderate
				C	0.650 ± 0.096								

N., number; I, intervention group; C, control group; HM, herbal medicine; ALE, alendronate; OP, osteoporosis; PMOP, postmenopausal osteoporosis; XLGB, xianlinggubao; MD, mean difference.

^a^ All studies were evaluated as ‘serious’ because randomization process and selection of the reported result were judged to have some concerns.

^b^ Subgroups showing high heterogeneity (I^2^ ≥ 50%) were evaluated as ‘Serious’, whereas those showing very high heterogeneity (I^2^ ≥ 75%) were evaluated as ‘Very serious’.

^c^ Funnel plot is asymmetric.

#### 3.4.2 HMs *plus* ALE versus ALE

HMs *plus* ALE group showed improved BMD score by 0.09 g/cm^2^ (28 RCTs, 95% CI: 0.07–0.10, *p* < 0.001, I^2^ = 78%) at the lumbar spine with very low certainty of evidence (Supplementary Figure S1; Table 4) and by 0.07 g/cm^2^ (17 RCTs, 95% CI: 0.05–0.08, *p* < 0.001, I^2^ = 64%) at the femoral neck with very low certainty of evidence (Supplementary Figure S2; Table 4). There was no significant difference in BMD improvement between PMOP and senile OP.

**FIGURE 5:**
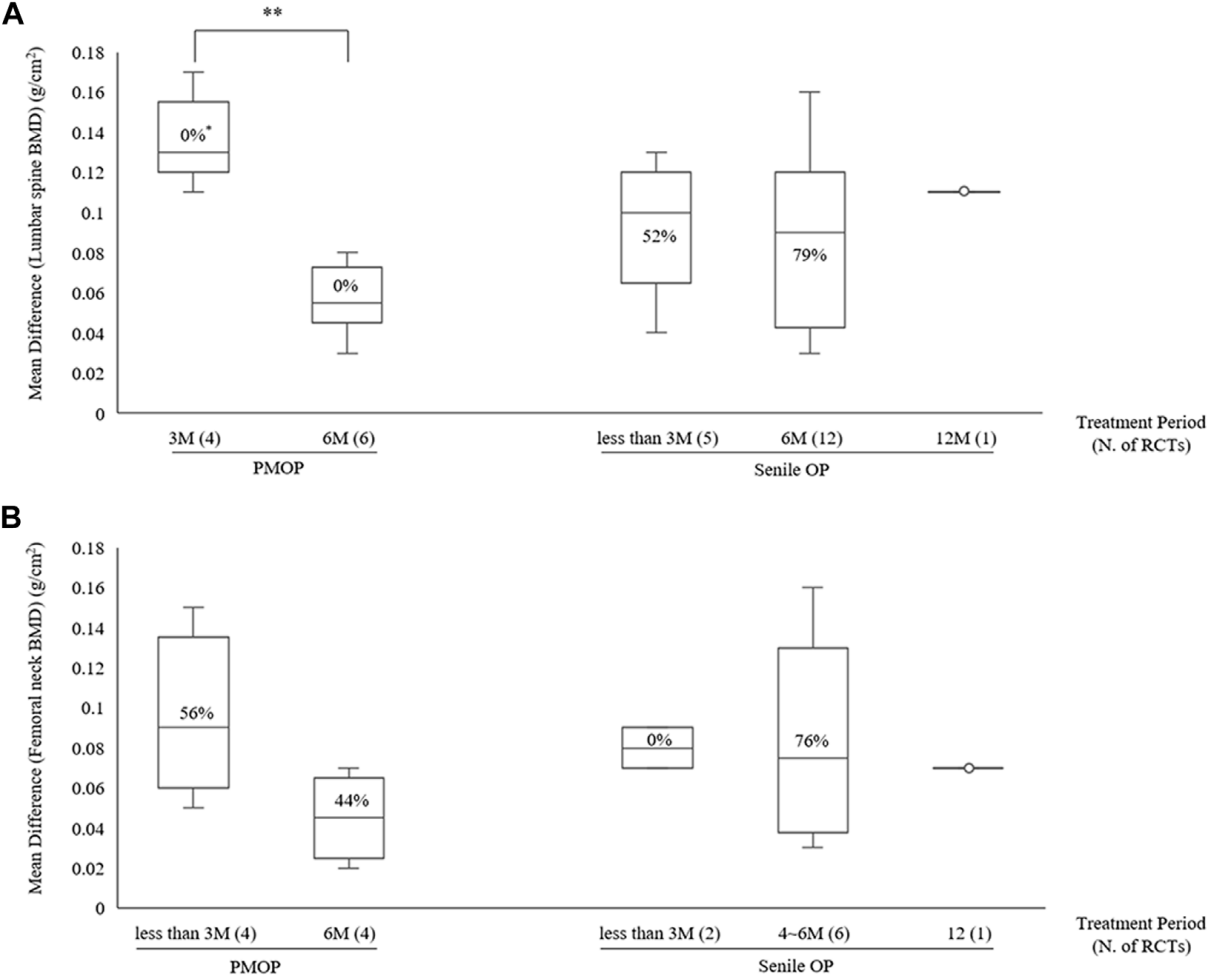
BMD improvement effects according to treatment period with combined HM and ALE therapy **(A)** Lumbar spine **(B)** Femoral neck. BMD, bone mineral density; HM, herbal medicine; ALE, Alendronate; M, month; PMOP, postmenopausal osteoporosis; OP, osteoporosis; N, number; RCT; randomized controlled trial; ^*^, I^2^.

Subgroup analysis based on treatment duration within OP type revealed that in PMOP, taking HMs with ALE for ≤3 months resulted in greater improvements in BMD compared to ALE alone, with a moderate certainty at the lumbar spine of 0.13 g/cm^2^ (4 RCTs, 95% CI: 0.12–0.15, *p* < 0.001, I^2^ = 0%) and low certainty at the femoral neck of 0.09 g/cm^2^ (4 RCTs, 95% CI: 0.05–0.12, *p* < 0.001, I^2^ = 56%) (Table 4; Figure 5; Supplementary Figures S4, S5). This was of greater magnitude compared to administering HMs *plus* ALE for 6 months. In senile OP, taking HMs with ALE for less than 3 months resulted in greater improvements in BMD compared to ALE alone, with a low certainty at the lumbar spine of 0.09 g/cm^2^ (5 RCTs, 95% CI: 0.06–0.13, *p* < 0.001, I^2^ = 52%) and moderate certainty at the femoral neck of 0.08 g/cm^2^ (2 RCTs, 95% CI: 0.05–0.12, *p* < 0.001, I^2^ = 0%), with no difference observed in the improvement when taken for 6 months (Table 4; Figure 5; Supplementary Figures S4, S5). Furthermore, sensitivity analysis revealed that excluding studies reporting outlier results (Xu et al., 2019; Liu and Wang, 2016) in the meta-analysis of HM plus ALE treated for less than 3 months and 6 months in the femoral neck of PMOP, respectively, and Cui et al. (2020) in the meta-analysis of HM plus ALE treated for less than 3 months in the lumbar spine of senile OP) led to a reduction in heterogeneity approaching '0′and an upward adjustment in the level of evidence (Supplementary Figures S6, S7).

#### 3.4.3 XLGB *plus* ALE versus ALE

**FIGURE 6:**
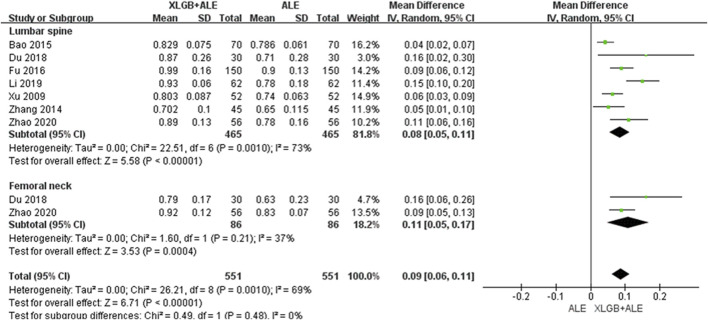
BMD improvement effect of the combined use of XLGB and ALE in primary OP. BMD, bone mineral density; XLGB, Xianlinggubao; ALE, Alendronate; OP, osteoporosis; SD, standard deviation; CI, confidence interval.

Compared to the ALE alone group, the combination group of the most commonly used herbal medicine XLGB with ALE demonstrated a significant improvement in BMD at the lumbar spine (7 RCT, 0.08 g/cm^2^, 95% CI: 0.05–0.11, *p* < 0.001, I^2^ = 73%) with low certainty and the femoral neck (2 RCT, 0.11 g/cm^2^, 95% CI: 0.05–0.17, *p* < 0.001, I^2^ = 37%) with moderate certainty of evidence (Figure 6; Table 4). Interestingly, upon excluding two studies, one with a duration of 3 months (Zhao et al., 2020) and another that presented an outlier result (Li et al., 2019), heterogeneity decreased from 73% to 47%, adjusting the level of evidence for the combined effect of XLGB and ALE at the lumbar spine (5 RCT, 0.06 g/cm^2^, 95% CI: 0.04–0.09, *p* < 0.001, I^2^ = 47%) to moderate (Supplementary Figure S8).

#### 3.4.4 Bone marker analysis of HMs *plus* ALE versus ALE

**FIGURE 7:**
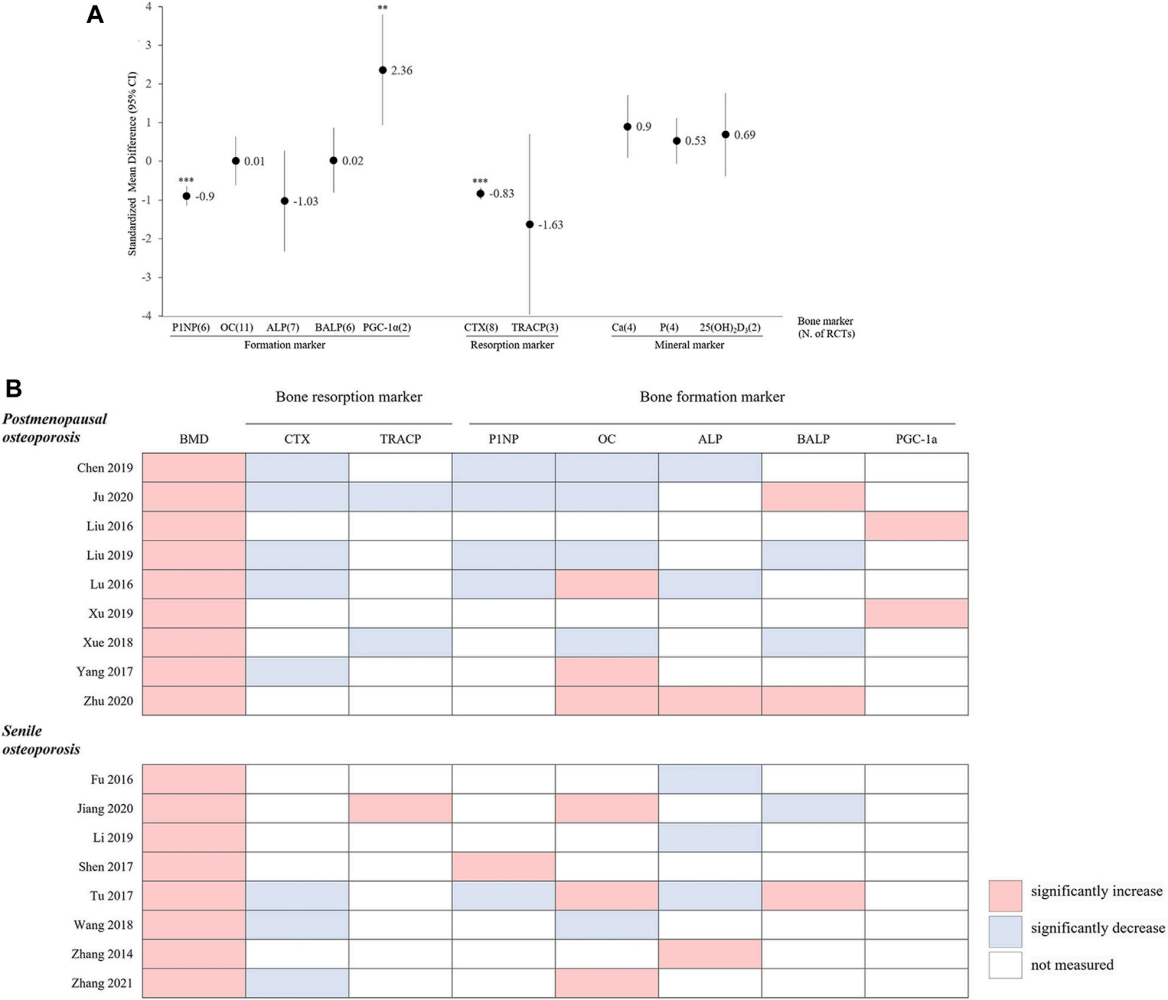
**(A)** Standardized mean difference in bone markers between HM *plus* ALE and ALE. P1NP, procollagen-1 N-terminal peptide; HM, herbal medicine; ALE, alendronate; OC, osteocalcin; ALP, alkaline phosphate; BALP, bone specific alkaline phosphate; SRC-3, Steroid Receptor Coabtivator-3; PGC-1α, Peroxi-some proliferator-activated receptor gamma coactivator 1-alpha; CTX, C-telopeptide of type 1 collagen; TRACP, Tartrate-resistant acid phosphatase; Ca, calcium; P, phosphorus; 25(OH)_2_D_3_, 25-hydroxivitaminD_3_; *: *p* < 0.05 in overall effect, **: *p* < 0.01 in overall effect, ***: *p* < 0.001 in overall effect. **(B)** The pattern of changes in BMD and bone markers in the HM *plus* ALE group. BMD, bone mineral density; CTX, C-telopeptide of type 1 collagen; TRACP, Tartrate-resistant acid phosphatase; P1NP, procollagen-1 N-terminal peptide; OC, osteocalcin; ALP, alkaline phosphate; BALP, bone specific alkaline phosphate; PGC-1α, Peroxi-some proliferator-activated receptor gamma coactivator 1-alpha.

Seventeen RCTs (PMOP in 9 RCTs; Senile OP in 8 RCTs) were included in the meta-analysis of bone markers (16 RCTs on bone formation markers, 10 RCTs on bone resorption markers, and seven RCTs on bone mineral markers), and 10 types of bone markers were analyzed. In these RCTs, the HMs *plus* ALE group, compared to the ALE alone, demonstrated a significant increase in PGC-1α (SMD: 2.36, 95% CI: 0.94–3.79, *p* < 0.01, I^2^ = 93%) as a bone formation marker and a significant decrease in P1NP (SMD: −0.9, 95% CI: −1.15 to −0.65, *p* < 0.001, I^2^ = 56%) as a bone formation marker and CTX (SMD: −0.83, 95% CI: −0.95 to −0.65, *p* < 0.001, I^2^ = 0%) as a bone resorption marker (Figure 7A). There were 10 RCTs that measured both bone formation and bone resorption markers, and a decrease in bone resorption markers was observed in the HMs *plus* ALE group compared to those in the ALE group, except for those observed in the study by Jiang et al., 2020 (Figure 7B). Notably, in PMOP participants taking HMs plus ALE, a concurrent reduction in both bone resorption and formation markers was observed, which was more pronounced compared to the trend seen in senile OP participants.

### 3.5 Adverse events

Out of the 43 RCTs, only 21 reported adverse events. Among these, 15 RCTs documented a total of 106 adverse events (48 in the intervention group and 58 in the control group), while 6 RCTs reported no adverse events. The most frequently occurring adverse events were gastrointestinal issues, with nausea and vomiting occurring in 36 patients, gastrointestinal reactions in 16, and diarrhea in 12 (Table 5). In each study, there was no significant difference between the intervention and control groups (*p* > 0.05).

**TABLE 5:** Adverse events reported in randomized controlled trials included in the review.

Adverse events	Intervention group	Control group	Total
Gastrointestinal reaction^a^	8	8	16
Diarrhea	7	5	12
Constipation	0	6	6
Nausea and vomiting	16	20	36
Hiccup	2	0	2
Dizziness	2	4	6
Dry mouth	3	0	3
Fever	1	3	4
Palpitation	2	1	3
Hypocalcemia	2	1	3
Rash	3	2	5
Fracture	2	8	10
Total	48	58	106

^a^ encompassing abdominal pain, dyspepsia, and tympanites.

### 3.6 Quality assessment

#### 3.6.1 Risk of bias

The overall risk of bias of every RCT included in our review was assessed to have “some concerns.”

All RCTs were judged to be of some concern as there was no information on whether there was allocation concealment during the randomization process. Not all studies blinded participants and assessors; however, as outcome measures such as BMD measured by DXA and bone markers measured by blood tests were not influenced by intended interventions, these studies were evaluated to have a “low risk” due to intended interventions. Additionally, none of the studies included in our review had previously published a study protocol, leading to some concerns regarding the selection of the reported results (Supplementary Figure S6).

#### 3.6.2 Publication bias

The presence of publication bias was confirmed in studies comparing the effectiveness of HMs combined with BPs to BPs alone in the femoral neck of patients with PMOP (*p*-value for bias: 0.008), as well as in studies comparing the effectiveness of HMs combined with ALE to ALE monotherapy in the femoral neck (*p*-value for bias: 0.001), as indicated by the funnel plot and Egger’s test. However, after adjusting for effect size using the trim-and-fill method, the estimated effects were reduced from 0.06 to 0.043 for studies on the HM + BP group in the femoral neck of patients with PMOP and from 0.07 to 0.049 for studies on the HM + ALE group in the femoral neck, while maintaining statistical significance. No evidence of publication bias was observed in other effect analyses (Supplementary Figure S7; Supplementary Tables S9A, S9B).

#### 3.6.3 Quality of evidence

In the comparison of HMs plus BPs with BPs, the quality of evidence ranged from “Very low” to “Low”. Meanwhile, the quality of evidence ranged from “Very low” to “Moderate” in the comparison of HMs plus ALE with ALE (Table 4). The quality of the evidence was not high primarily due to the high risk of bias and heterogeneity observed in the RCTs included in each meta-analysis, leading to a downgrade. Therefore, in the subgroup analysis divided by OP type and treatment duration, there was a tendency for heterogeneity to decrease while the grade of evidence increased.

## 4 Discussion

OP occurs when bone resorption exceeds bone formation in the bone remodeling process due to estrogen deficiency and aging (Nuti et al., 2019; Föger-Samwald et al., 2022). Because OP consequently increases the risk of fractures (Cosman et al., 2014), which in turn reduce quality of life and increase mortality in older people (Johnston and Dagar, 2020; Lee and Nam, 2021), prompt OP treatment to prevent fractures is crucial (Kanis et al., 2019).

BPs are powerful bone resorption inhibitors recommended as first-line agents for the treatment of OP (Rogers, 2003; Fuggle et al., 2019). In contrast, HMs have the advantage of promoting bone formation while simultaneously inhibiting bone resorption through multi-targeting (Leung and Siu, 2013; Jin et al., 2017). Given the characteristics of these two medications, a systematic review and meta-analysis were conducted to evaluate the combined effects of HMs and BPs on improving BMD in patients with primary OP.

As a result, the addition of HMs showed a potential for improving BMD compared to the use of BPs alone, regardless of the type of OP or the BMD measurement site. During the same treatment period, the addition of HMs led to approximately a 2.3 times increase (MD: 0.10 g/cm^2^) in BMD in the lumbar spine and approximately a 2.1 times increase (MD: 0.08 g/cm^2^) in BMD in the femoral neck compared to the use of BPs alone (Table 4; Figures 2, 3). However, the risk of bias and heterogeneity among the RCTs included in this review was very high, resulting in an overall certainty of evidence rated as “very low” to “low” (Table 4). Accurate assessment of intervention effects requires appropriate blinding and allocation concealment, and these processes should be reported in detail in the studies. Although the RCTs included in our review employed random sampling, specific details on blinding and allocation concealment were not provided. Despite the likely minimal impact of blinding deficiencies on outcome measures such as BMD assessments using DXA and blood tests, this represents a significant limitation in the interpretation of our review results.

In total, 4,470 participants (1,261 males and 3,209 females) were included in the present study, with a male-to-female ratio of 1:2.5. Of these, 597 had fractures. The mean age of patients with senile OP was approximately 8 years higher than that of patients with PMOP (68.7 ± 5.3 years for senile OP vs 59.9 ± 4.6 years for PMOP), and the duration of OP was approximately 1.5 years longer. Since we classified the OP types according to the definitions provided in each RCT, there was no overlap among the participants in the 43 RCTs. When referencing prior literature that distinguishes between PMOP and senile OP around the age of 70 (Aibar-Almazán et al., 2022; Sözen et al., 2017), the average ages for the OP types provided in Table 1 appear logical. However, due to the lack of detailed age-related distribution information in each RCT, it is not possible to exclude the possibility that some female participants of the same age might be classified under both PMOP and senile OP.

The initial BMD was 0.721 ± 0.066 g/cm^2^ in the lumbar spine, 0.646 ± 0.068 g/cm^2^ in the femoral neck, and 0.625 ± 0.098 g/cm^2^ in the total hip (Table 1). These BMD values were lower than those of the general population of the same age but similar to the BMD values of patients with OP reported in previous studies (Zhang and Shang, 2014; Zhao et al., 2021). As is well known, risk factors for OP include age, sex, diet, physical activity, weight, smoking, alcohol consumption, and genetic factors (Pouresmaeili et al., 2018). All participants in this review were Chinese, with no identifiable information available regarding OP risk factors other than age and sex.

Observing the BMD change pattern at the lumbar spine of the general Chinese population (Zeng and He, 2015), peak bone mass (1.077 g/cm^2^) is reached in the 20s and 30s age groups; BMD decreases by approximately 5% (0.045 g/cm^2^) on average every 10 years. After their 50s, men and women show different bone loss patterns. Due to these characteristic differences, primary OP can be classified into two types: PMOP and senile OP. In their 50s–60s, patients undergo a rapid decline in BMD, particularly in women who show a rapid decrease of 8%–13% due to menopause. Furthermore, the BMD of both men and women reaches approximately 0.902 g/cm^2^ when they reach their 60s. Considering the pattern of BMD changes among these Chinese individuals, our data suggests that the combination of HMs and BPs may improve the BMD of patients with primary OP, making their BMD levels similar to those of individuals approximately 10–15 years younger. However, the certainty of the evidence for the combined effects of HMs and BPs in our study was generally rated as “very low” to “low” due to high heterogeneity and risk of bias (Table 4).

The high heterogeneity is presumed to be due to the diversity in OP types, duration of treatment, and types of HMs used. Subgroup analysis based on OP type and treatment duration substantially reduced heterogeneity, indicating a moderate certainty of BMD improvement. Specifically, when HMs were combined with ALE, compared to ALE alone, for 6 months in patients with PMOP, a greater increase of 0.05 g/cm^2^ (6 RCTs, 95% CI: 0.04–0.07, *p* < 0.001, I^2^ = 0%) in the lumbar spine and 0.04 g/cm^2^ (4 RCTs, 95% CI: 0.02–0.06, *p* < 0.001, I^2^ = 44%) in the femoral neck was observed. Additionally, in patients with Senile OP, a greater increase of 0.08 g/cm^2^ (2 RCTs, 95% CI: 0.05–0.12, *p* < 0.001, I^2^ = 0%) in the femoral neck was observed after approximately 3 months of treatment compared to ALE alone (Supplementary Figure S4, S5; Table 4).

The notable finding from the subgroup analysis of HMs plus ALE is that, for senile OP, treatment duration did not significantly affect the Mean Difference (MD). However, in PMOP, adding HMs led to a greater MD improvement at 3 months compared to ALE alone, although this difference decreased at 6 months (Figure 5). This pattern in PMOP, which includes only women, may suggest a gender-based difference in response to BPs and that HMs might initially improve BMD. Nonetheless, due to the lack of individual data for men and women, the exact cause of this effect remains unclear.

In the RCTs included in our review, a total of 28 types of HMs were administered. Among these, 17 were traditional decoctions, while 11 were modernized formulations in the form of capsules, granules, or pills. Of the modernized formulations, 7 types with reported active compounds from previous studies included Xianlinggubao capsule (XLGB), Gushukang capsule, Qianggu capsule, Kuntai capsule, Tenghuang Jiangu capsules, Biqi capsule, and Liuwei Dihuang pill (Bao et al., 2020; Chai et al., 2019; Jiannong et al., 2015; Liu and Sun, 2023; Li et al., 2023; Jia et al., 2024; Wang et al., 2013). These HMs contained active compounds that promote osteoblast activity and inhibit osteoclast activity, including quercetin, icariin, naringin, and others (Supplementary Table S3). Classifying HMs based on these chemical compounds and evaluating their effects on BMD proved challenging due to the wide variety of compounds present in traditional HMs.

Nevertheless, we attempted to classify the HMs according to three pattern identifications (kidney yang deficiency, syndrome of qi stagnation and blood stasis, and liver-kidney yin deficiency) and conducted a subgroup analysis to assess BMD improvement effects. However, no significant differences were found between the groups (Supplementary Figure S8, S9).

Meanwhile, a meta-analysis of the BMD improvement effects of XLGB, which was frequently prescribed in 11 RCTs in our review, revealed a reduction in heterogeneity and demonstrated a similar degree of overall BMD improvement as the concurrent use of HMs and BPs. The combination of XLGB and ALE showed a tendency for more BMD improvement compared to ALE alone, with results observed in the lumbar spine (7 RCTs, 0.08 g/cm^2^, 95% CI: 0.05–0.11, *p* < 0.001, I^2^ = 73%) and femoral neck (2 RCTs, 0.11 g/cm^2^, 95% CI: 0.05–0.17, *p* < 0.001, I^2^ = 37%). Consequently, the level of evidence for this combination was upgraded to ‘moderate’ (Figures 2, 3, 6; Table 4).

XLGB is one of the most recommended traditional Chinese medicines for OP treatment (Xing et al., 2013). It comprises *E. koreanum Nakai* [Berberidaceae; Epimedii Herba], *Dipsacus asper Wall. ex Henry* [Dipsacaceae; Dipsaci Radix], *C. corylifolium (L.) Medik* [Fabaceae; Psoraleae Fructus], *R. glutinosa (Gaertn.) DC* [Orobanchaceae; Rehmanniae Radix], *Salvia miltiorrhiza Bunge* [Lamiaceae; Salviae Miltiorrhizae Radix et Rhizoma], and *Anemarrhena asphodeloides Bunge* [Liliaceae; Anemarrhenae Rhizoma], which are also among the most frequently used botanical drugs in the RCTs included in this study (Table 3; Supplementary Table S2). Furthermore, XLGB contains 146 major compounds, including key active compounds related to bone metabolism such as quercetin (Satué et al., 2013), luteolin (Kim et al., 2011; Nash et al., 2015), kaempferol (Wong et al., 2019), anhydroicaritin (Zheng et al., 2017), and diosgenin (Alcantara et al., 2011; Zhang et al., 2017). These compounds have been shown to promote osteoblast activity while inhibiting osteoclast activity.

The efficacy of XLGB is not attributable to a single mechanism of action. It involves various anti-osteoporotic mechanisms, including the PI3K-Akt-mTOR signaling pathway (Bao et al., 2020), inhibition of glycogen synthase kinase-3β and cathepsin K (Qiu et al., 2021), the cAMP signaling pathway, and the calcium signaling pathway (Zeng et al., 2024). Similar characteristics are also found in other HMs with reported bioactive compounds.

In contrast, BPs are derivatives of inorganic pyrophosphates and form a P-C-P bond, which gives them a strong affinity for calcium phosphate, thereby inhibiting both normal and ectopic mineralization (Fleisch et al., 1969). The mechanism of action of BPs in inhibiting bone resorption involves the suppression of farnesyl pyrophosphate (FPP) synthase and/or isopentenyl pyrophosphate isomerase. This inhibition disrupts the mevalonate pathway, which is crucial for the prenylation of small GTPase signaling proteins, ultimately leading to the inhibition of osteoclast activity and, consequently, bone resorption (Dunford et al., 2001; Van Beek et al., 2002).

We found that naringin, the active compound of *Drynaria fortunei (Kunze) J. Sm* [Polypodiaceae; Drynariae Rhizoma] often used in the included RCTs, inhibits the mevalonate pathway. This mechanism is similar to that of BPs (Hirata et al., 2009; Wu et al., 2021). However, it is unclear whether the improvement in bone mineral density by Gushukang and Qianggu capsules, both containing naringin, is due to this same pathway inhibition. Unlike BPs, which target specific pathways, HMs contain multiple active compounds. This makes it difficult to confirm if their primary mechanism for improving BMD is similar to that of BPs.

BTMs are a useful adjunct for the diagnosis and therapeutic monitoring of bone metabolic disorders (Greenblatt et al., 2017) The bone resorption marker CTX was significantly decreased in the HMs *plus* ALE group compared to the ALE group. However, the increase in bone formation markers was not consistent (Figure 7A).

BPs are known to strongly inhibit bone resorption. Due to their mechanism of maintaining the balance between resorption and formation during bone remodeling, they typically result in a reduction in bone formation rates as well (Jensen et al., 2021). In particular, PMOP patients often exhibit an abnormal increase in both bone resorption and formation markers approximately 5 years post-menopause. This increase then normalizes with BP treatment, leading to a concurrent decrease in both markers and a reduction in the rate of bone formation (Kuo and Chen, 2017). In our review, among the 10 RCTs that observed both resorption and formation markers, a trend was noted where both markers decreased and BMD improved, particularly in PMOP participants compared to those with senile OP (Figure 7B). Thus, when analyzing BTMs, it is crucial to assess not only their simple increases or decreases but also the balance between bone formation and resorption and the rate of normal bone remodeling (Hu et al., 2013). Additionally, bone markers are sensitive indicators affected by factors such as age, sex, and circulation. Therefore, it is important to consider the timing and conditions under which the tests are performed. Nevertheless, few studies have accurately specified the conditions and test procedures in the RCTs included in this review. Future studies need to establish accurate test protocols and report findings clearly for the scientific evaluation of bone markers.

In our study, the incidence of adverse events in the HM *plus* BPs group was 4.3%, with gastrointestinal reactions being the most commonly observed type. These reactions are consistent with those typically associated with both BPs and HMs. The common adverse effects of BPs include gastrointestinal reactions (28%–91%) (Tadrous et al., 2014) and musculoskeletal reactions (20.1%–25.0%) (Pazianas and Abrahamsen, 2011). Similarly, adverse events reported for HMs used in OP predominantly involve gastrointestinal issues such as diarrhea, constipation, nausea, and vomiting, with incidence rates ranging from 8.69% to 29.4% (Jia et al., 2022; Shi et al., 2017; Li et al., 2017). Similar to our study, the adverse events of HMs combined with conventional drugs such as BPs for OP also showed similar findings, with gastrointestinal reactions such as nausea, vomiting, constipation, and diarrhea occurring at rates of 5.72%–5.76% (Chen et al., 2021; Kwon et al., 2023). Consequently, the adverse reactions associated with the combination of HMs and BPs in our study were comparable to those reported in the existing literature, with no serious adverse effects observed.

However, among the 43 RCTs included in our review, it remains unclear whether 20 of these trials specified cardiovascular diseases or breast cancer - conditions potentially influenced by BPs - as exclusion criteria. Additionally, the interactions between BPs and HMs are not yet well understood. Therefore, the safety of combining HMs and BPs remains an unresolved issue, and further research is needed to elucidate the interactions and potential risks associated with these two treatments.

Our study has several limitations. Firstly, the included RCTs are characterized by generally low quality, high risk of bias, and significant heterogeneity, resulting in a low level of evidence. Secondly, the lack of separate information on participant sex and age, coupled with difficulties in verifying the diagnostic criteria for PMOP and senile OP in each RCT, hampers clear identification of characteristics based on OP types. Thirdly, the absence of data on factors influencing OP in each RCT prevents the exclusion of potential confounders that could affect BMD improvements. Fourthly, there is insufficient information regarding the quality control, extraction processes, and chemical analysis of HMs, as well as a lack of safety data on the combination of HMs and BPs. Additionally, our review did not include grey literature and conference abstracts, which may contribute to publication bias and limit the perspective on various approaches.

Despite these limitations, our study is significant as it represents the first systematic review to assess the BMD improvement effects of combination therapy with HMs and BPs in patients with primary OP and to evaluate the certainty of the evidence. Additionally, the number of included RCTs and the overall sample size were sufficiently large for analysis. Based on our findings, to investigate the effects of HMs and BPs in OP patients, future studies should employ RCTs with low risk of bias and utilize a double-blind design incorporating placebo controls for HMs. These studies should include standardized protocols for HMs, detailed information on quality control, and safety data. Additionally, it is crucial to control for confounding factors that may impact BMD as thoroughly as possible.

In the future, to provide scientific evidence for the effectiveness of HM combination therapy in patients with primary OP, a well-designed, large-scale clinical study that compensates for the limitations of previous research is needed.

## Figures and Tables

**Table:** 

**25(OH)**_**2**_ **D**_**3**_	25-hydroxyvitamin D3
**AMSTAR2**	A Measurement Tool to Assess Systematic Reviews
**ALE**	Alendronate
**ALP**	Alkaline phosphate
**BMD**	Bone mineral density
**BALP**	Bone-specific alkaline phosphate
**BP**	Bisphosphonate
**CTX**	C-telopeptide of type 1 collagen
**Ca**	Calcium
**CNKI**	China National Knowledge Infrastructure
**RoB2**	Risk of Bias 2
**CI**	Confidence intervals
**ConPhyMP**	Consensus statement on the Phytochemical Characterisation of Medicinal Plant extracts
**DOI**	Digital Object Identifier
**DXA**	Dual-energy X-ray absorptiometry
**EGFR**	Epidermal Growth Factor Receptor
**ERK**	Extracellular-signal-regulated kinase
**FPP**	Farnesyl pyrophosphate
**GPR30**	G protein-coupled estrogen receptor
**GRADE**	Grading of Recommendations Assessment, Development, and Evaluation
**HM**	Herbal medicine
**HRT**	Hormone Replacement Therapy
**IOF**	International Osteoporosis Foundation
**JNK**	c-Jun N-terminal kinase
**KISS**	Koreanstudies Information Service System
**MAPK**	Mitogen-activated protein kinase
**MD**	Mean difference
**OP**	Osteoporosis
**OC**	Osteocalcin
**PTH**	Parathyroid hormone
**PGC-1α**	Peroxisome proliferator-activated receptor gamma coactivator 1-alpha
**P**	Phosphorus
**PMOP**	Postmenopausal osteoporosis
**PRISMA**	Preferred Reporting Items for Systematic Reviews and Meta-Analyses
**P1NP**	Procollagen-1 N-terminal peptide
**PROSPERO**	Prospective Register of Systematic Reviews
**RANKL**	Receptor activator of nuclear factors κB ligand
**RCT**	Randomized controlled trial
**ROS**	Reactive oxygen species
**SD**	Standard deviation
**SERMs**	Selective estrogen receptor modulators
**SMD**	Standardized mean difference
**TRACP**	Tartrate-resistant acid phosphatase
**U-Ca**	Urine calcium
**WHO**	World Health Organization
**XLGB**	Xianlinggubao capsule
**ZOL**	Zoledronate

## Data Availability

The original contributions presented in the study are included in the article/Supplementary Material, further inquiries can be directed to the corresponding author.
