# Supplementary material for: Additional effects of herbal medicine combined with bisphosphonates for primary osteoporosis: a systematic review and meta-analysis
Source: Front Pharmacol. 2024 Sep 13;15:1413515. doi: 10.3389/fphar.2024.1413515 (PMC11427380; doi:10.3389/fphar.2024.1413515)
Supplement: Supplementary file 1 [file DataSheet1.docx]

Supplementary Material

Additional Effects of Herbal Medicine Combined with Bisphosphonates for Primary Osteoporosis: A Systematic Review and Meta-Analysis

Young-Seo Yoo^†^, Min-Gyeong Kim^†^, Hee-Joo Park, Min Young Chae, Yu-Jin Choi, Chae-Kun Oh, Chang-Gue Son, Eun-Jung Lee^*^

†These authors contributed equally to this work and share first authorship.

*** Correspondence:** Dr. Eun-Jung Lee: [jungkahn@dju.kr](mailto:jungkahn@dju.kr)

# Supplementary Figures and Tables

## Supplementary Figures


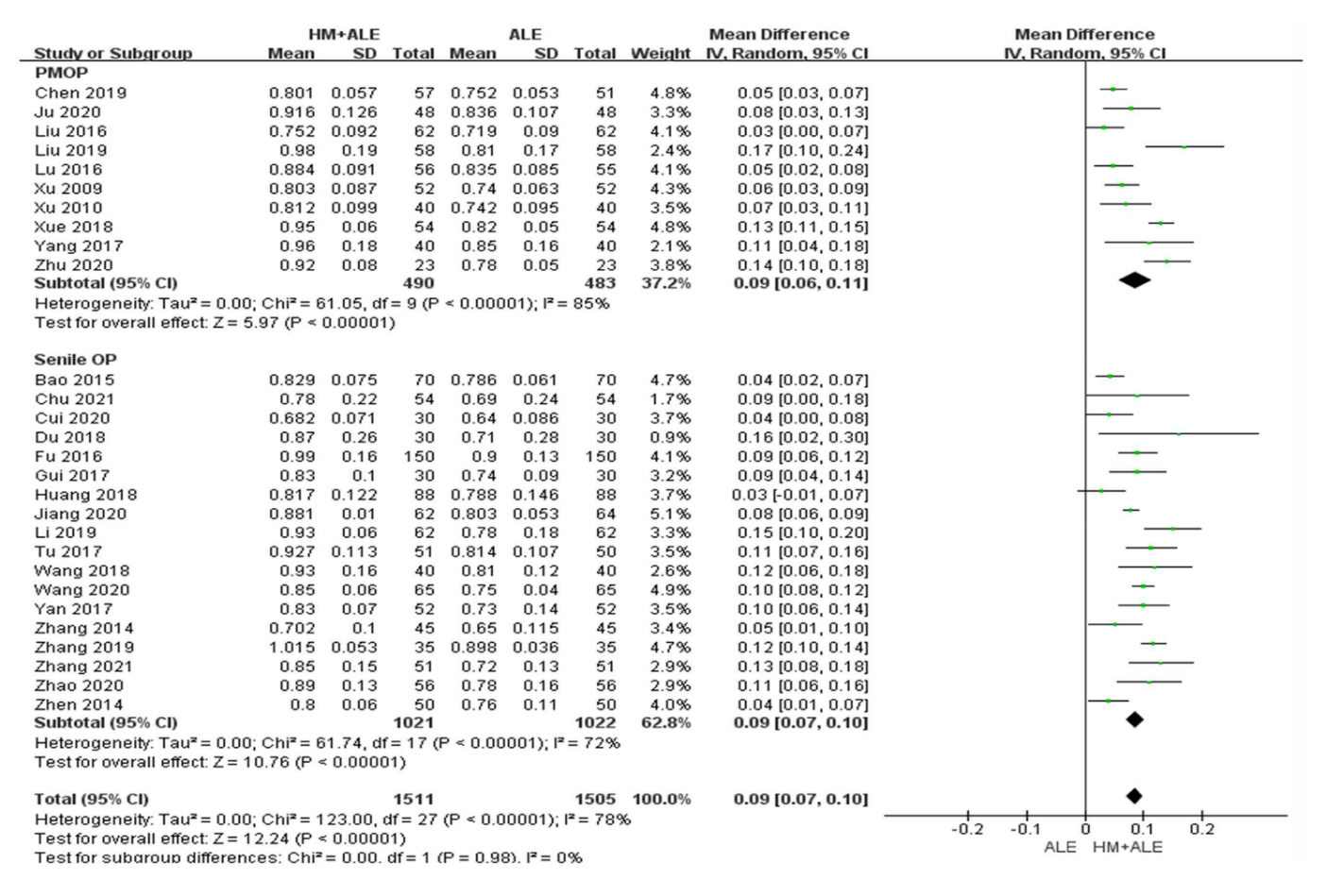


**Supplementary Figure S1.** The BMD improvement effects of combined therapy of HM and ALE at the lumbar spine

BMD, bone mineral density; HM, herbal medicine; ALE, alendronate; PMOP, postmenopausal osteoporosis; SD, standard deviation; CI, confidence interval


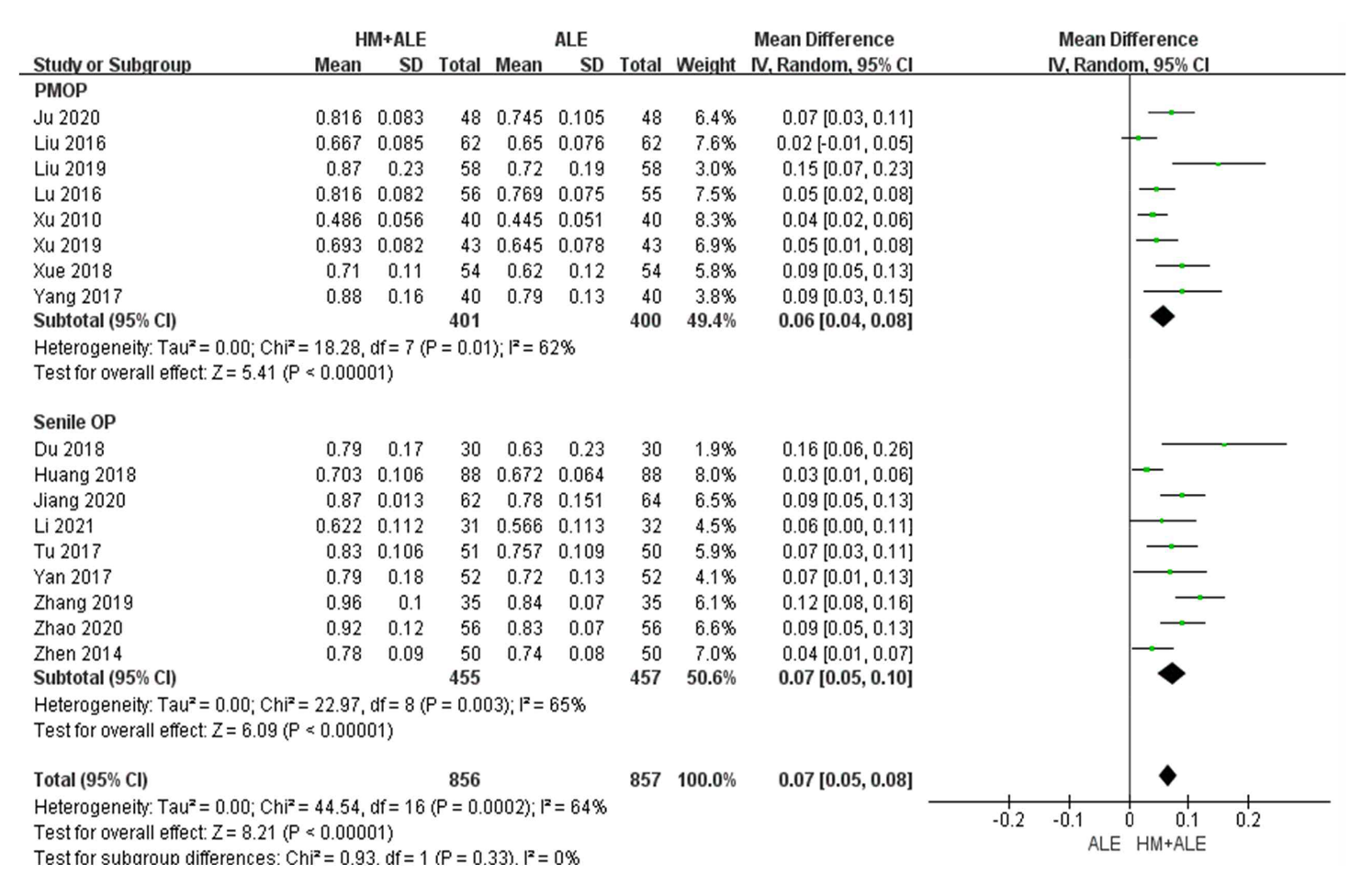


**Supplementary Figure S2.** The BMD improvement effects of combined therapy of HM and ALE at the femoral neck

BMD, bone mineral density; HM, herbal medicine; ALE, alendronate; PMOP, postmenopausal osteoporosis; SD, standard deviation; CI, confidence interval

(A)


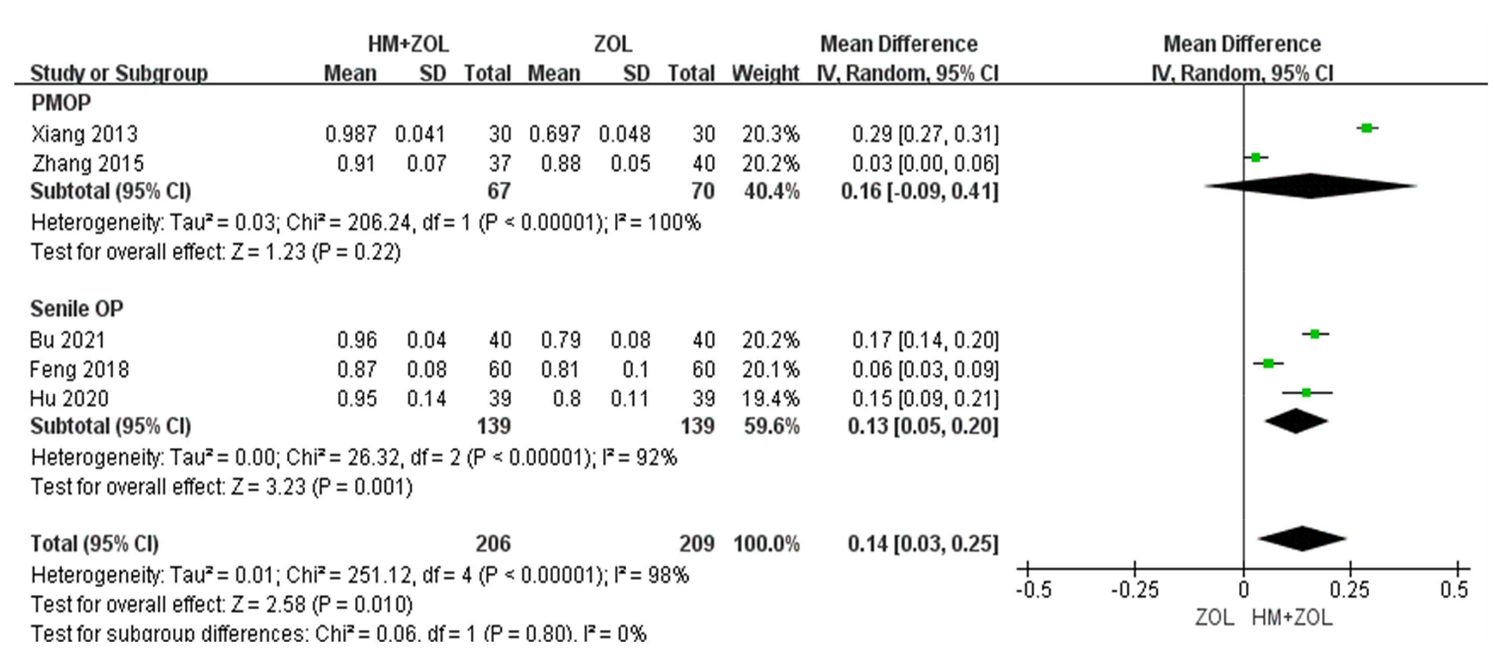


(B)


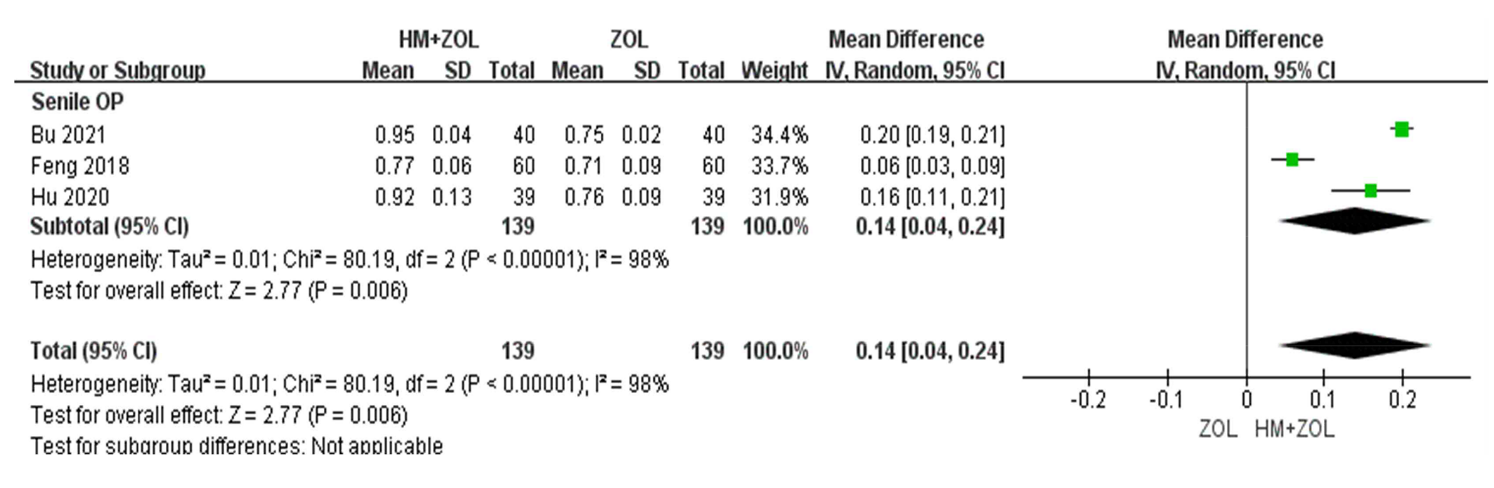


**Supplementary Figure S3.** The BMD improvement effects of combined therapy of HM and ZOL at the lumbar spine (A) and the femoral neck (B)

BMD, bone mineral density; HM, herbal medicine; ZOL, zoledronate; PMOP, postmenopausal osteoporosis; SD, standard deviation; CI, confidence interval

(A)


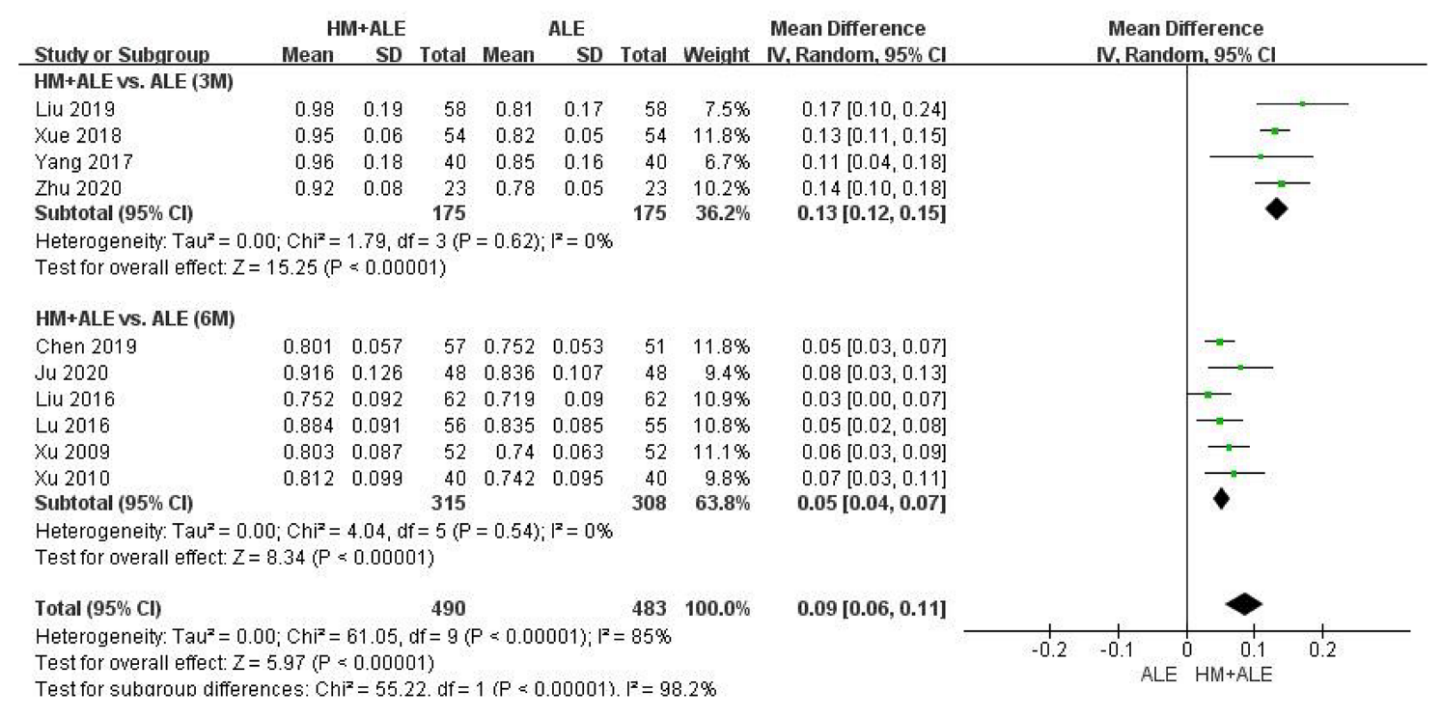


(B)


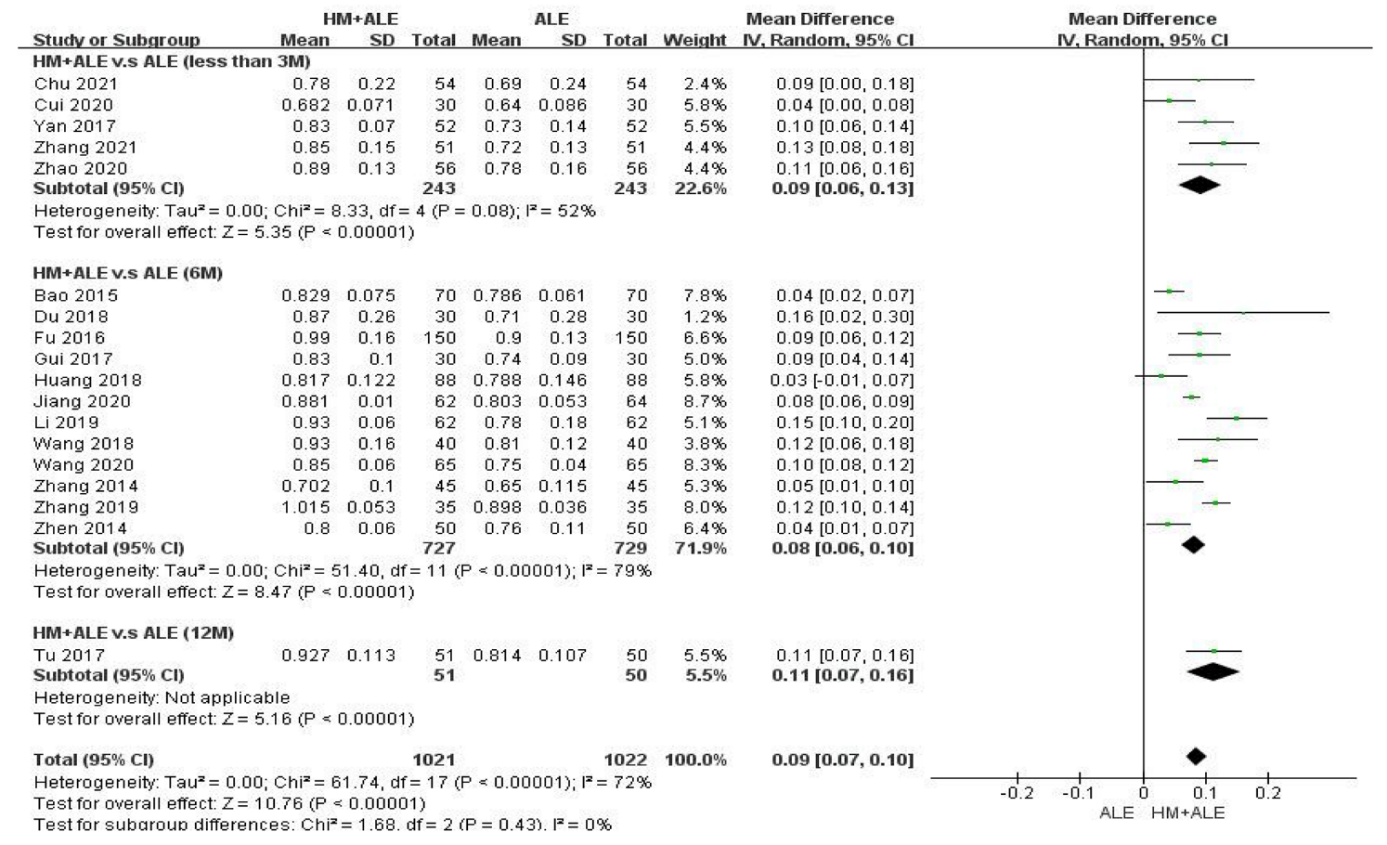


**Supplementary Figure S4.** The BMD improvement effects at the lumbar spine with combined HM and ALE therapy in PMOP (A) and Senile OP (B)

BMD, bone mineral density; HM, herbal medicine; ALE, alendronate; PMOP, postmenopausal osteoporosis; SD, standard deviation; CI, confidence interval

(A)


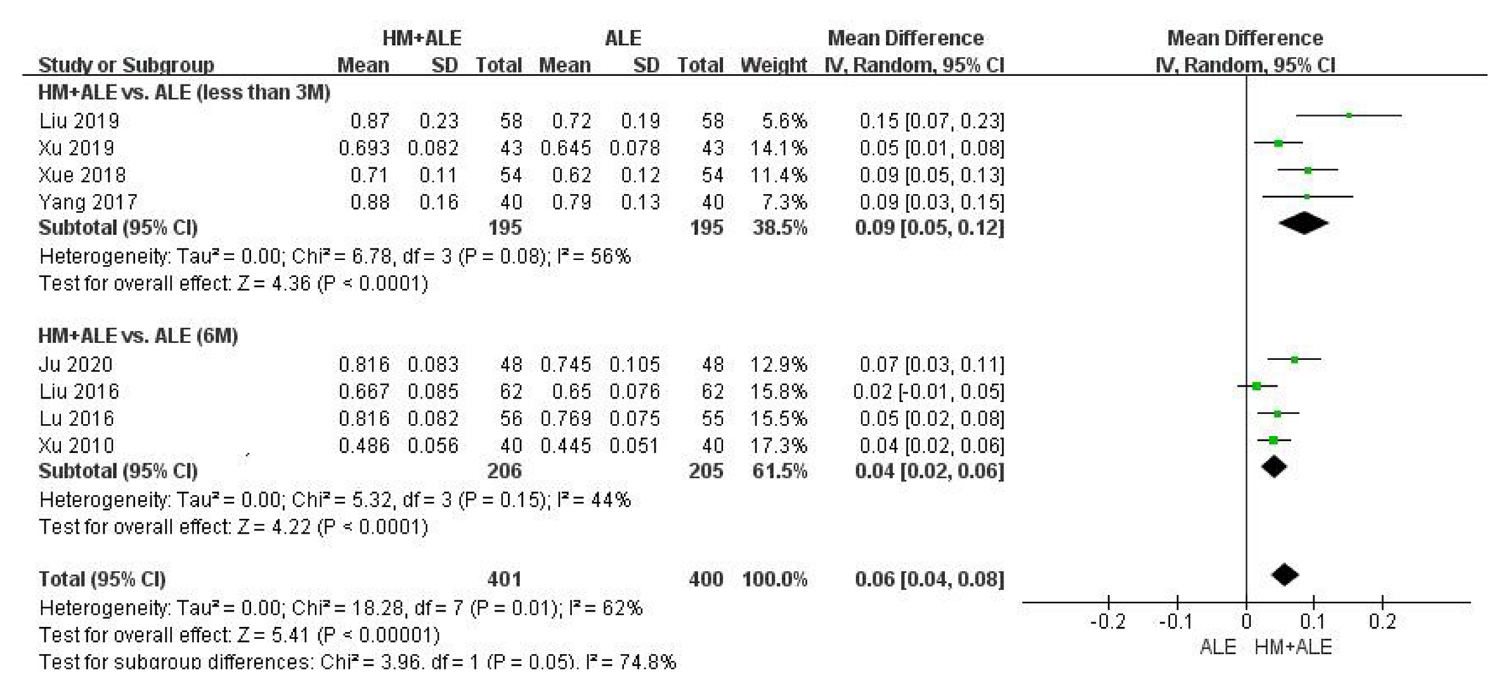


(B)
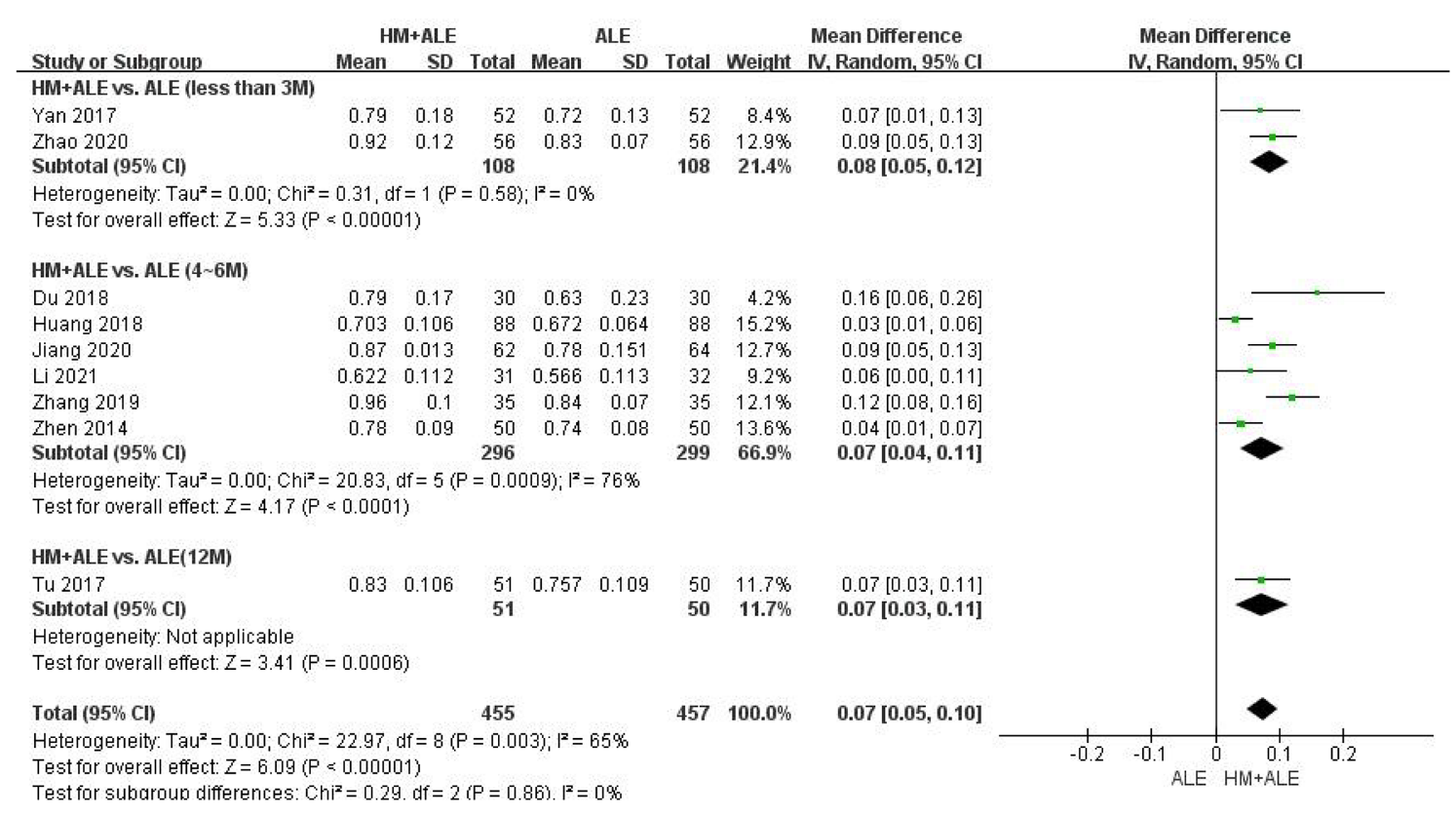


**Supplementary Figure S5.** The BMD improvement effects at the femoral neck with combined HM and ALE therapy in PMOP (A) and Senile OP (B)

BMD, bone mineral density; HM, herbal medicine; ALE, alendronate; PMOP, postmenopausal osteoporosis; SD, standard deviation; CI, confidence interval

(A)


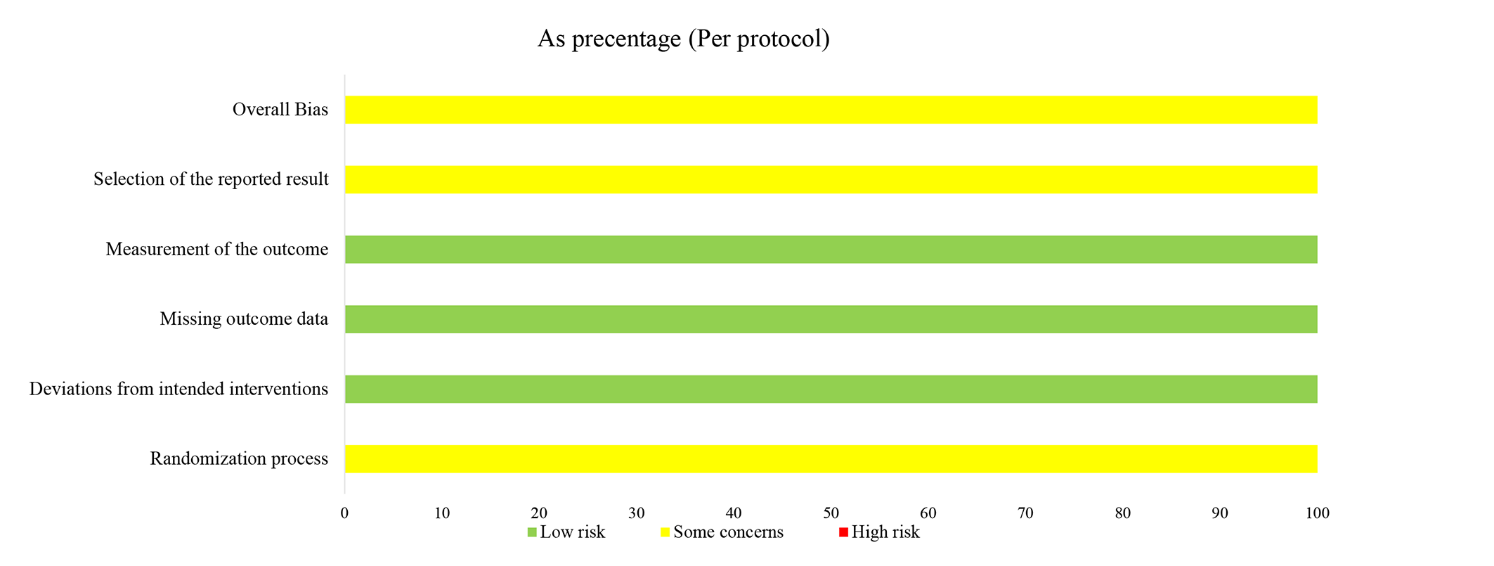


(B)
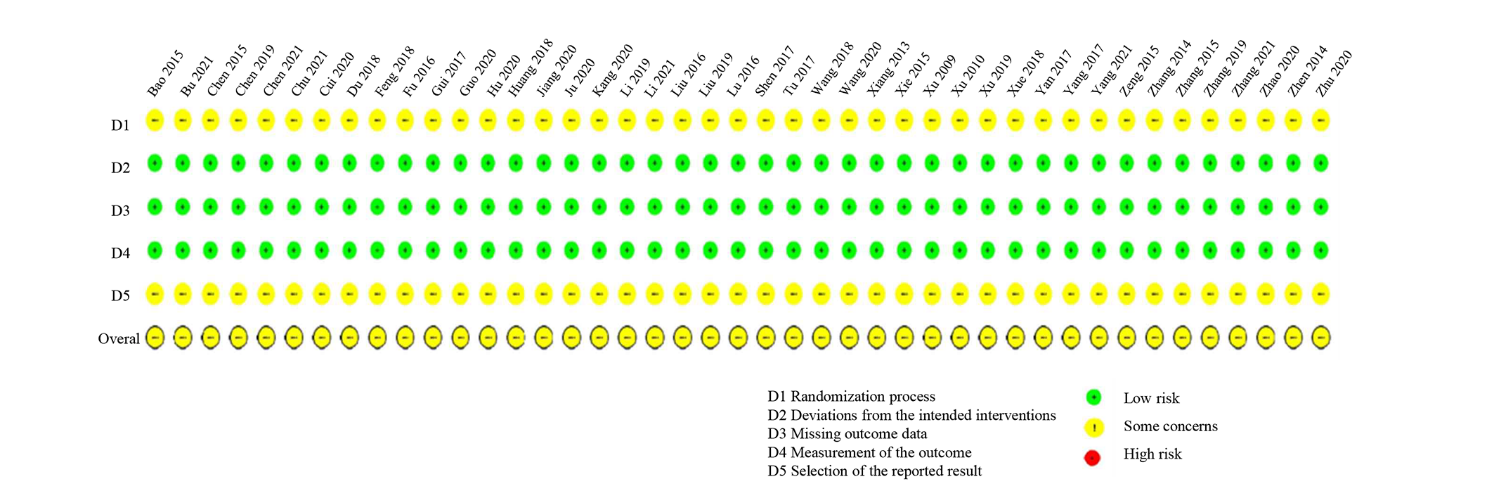


**Supplementary Figure S6.** The summary and the graph of risk of bias

(A) (B)


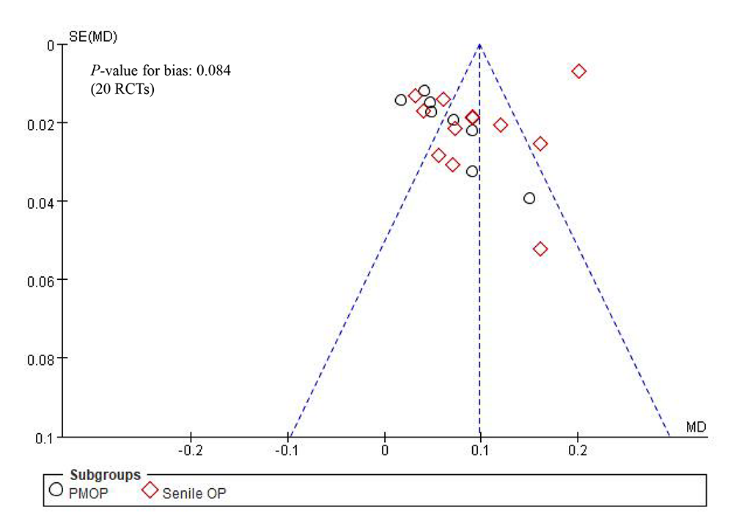

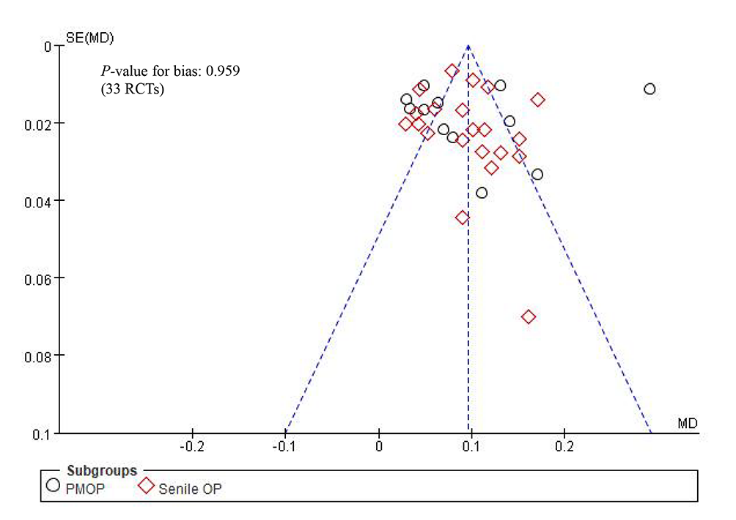


(C) (D)


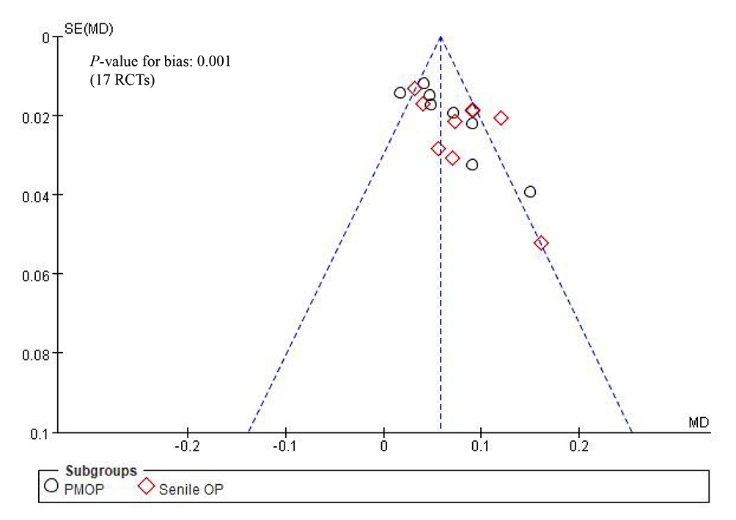

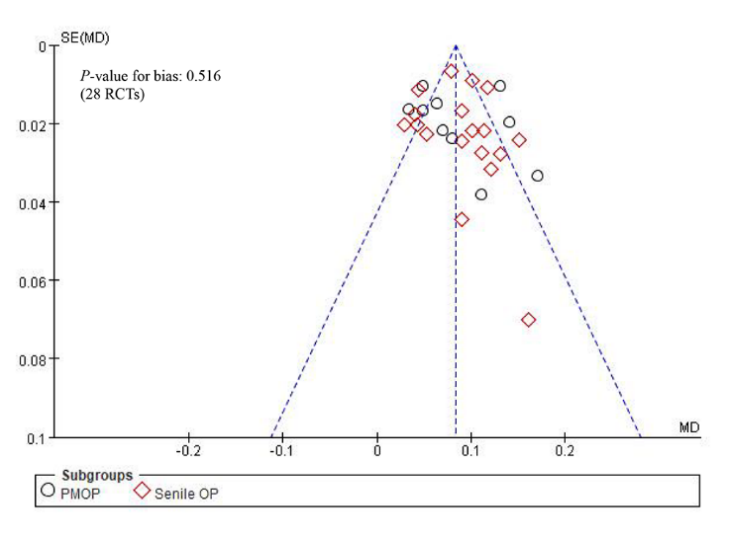


**Supplementary Figure S7.** Publication bias in the analysis of the effects of HM and BPs in the lumbar spine (A), HM plus BPs at the femoral neck (B), HM plus ALE at the lumbar spine (C), and HM plus ALE at the femoral neck (D)

HM, herbal medicine; BP, bisphosphonate; ALE, alendronate

(A)

**
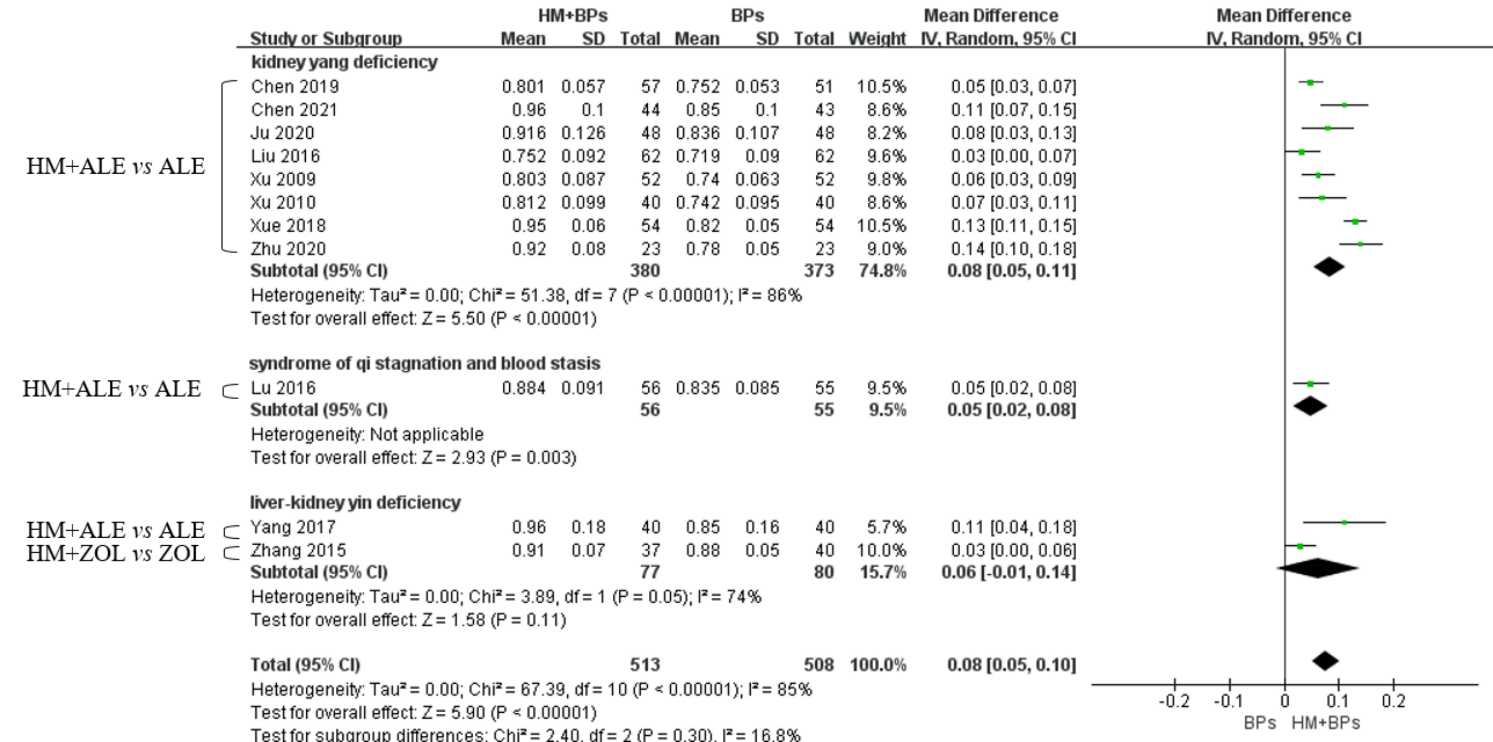
**

(B)

**
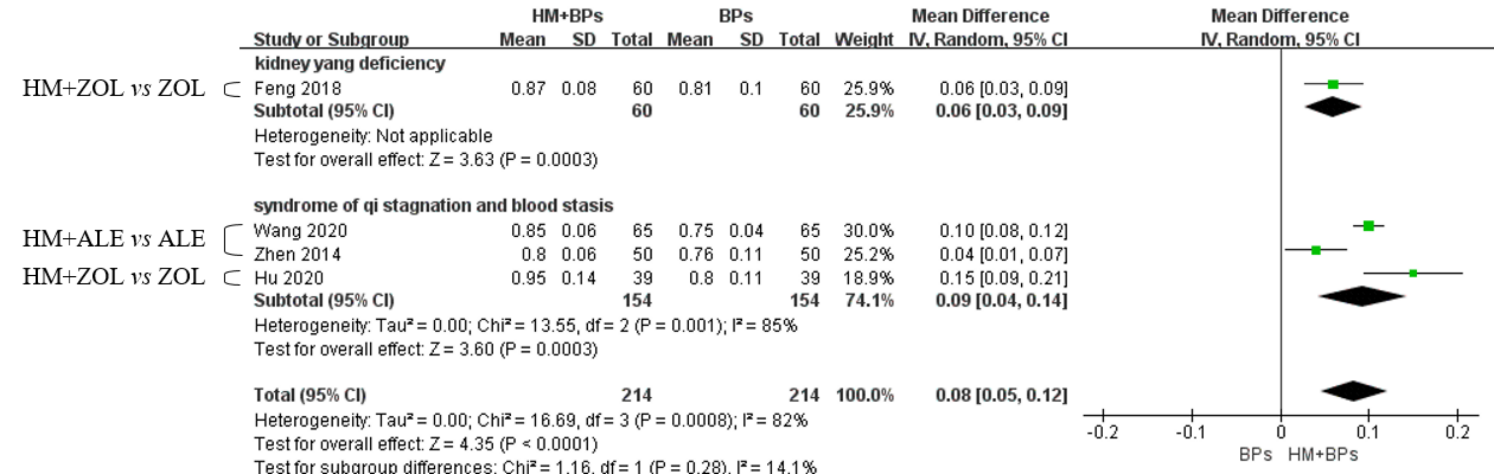
**

**Supplementary Figure S8.** The BMD improvement effects according to prescription pattern identification of HM and BPs at the lumbar spine in PMOP (A) and Senile OP (B)

BMD, bone mineral density; HM, herbal medicine; BPs, bisphosphonates; ALE, alendronate; ZOL, zoledronate; PMOP, postmenopausal osteoporosis; SD, standard deviation; CI, confidence interval

(A)

**
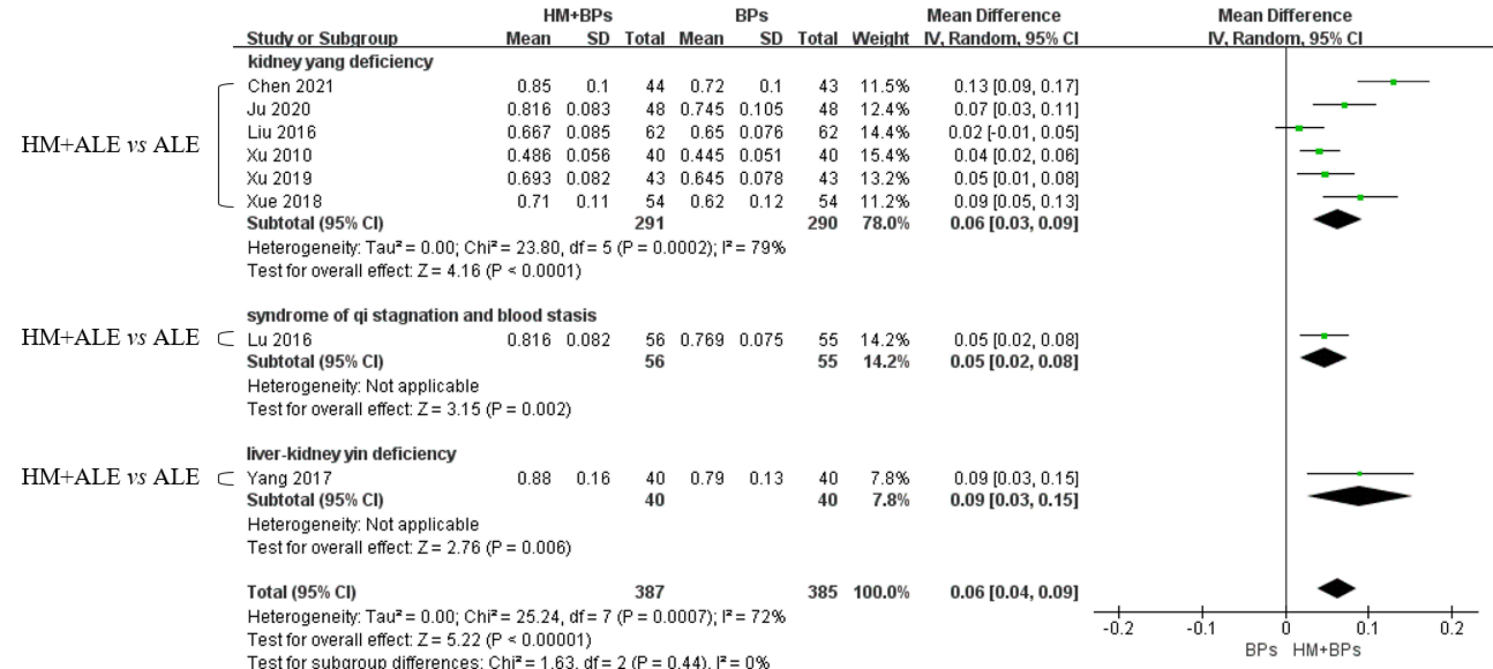
**

(B)

**
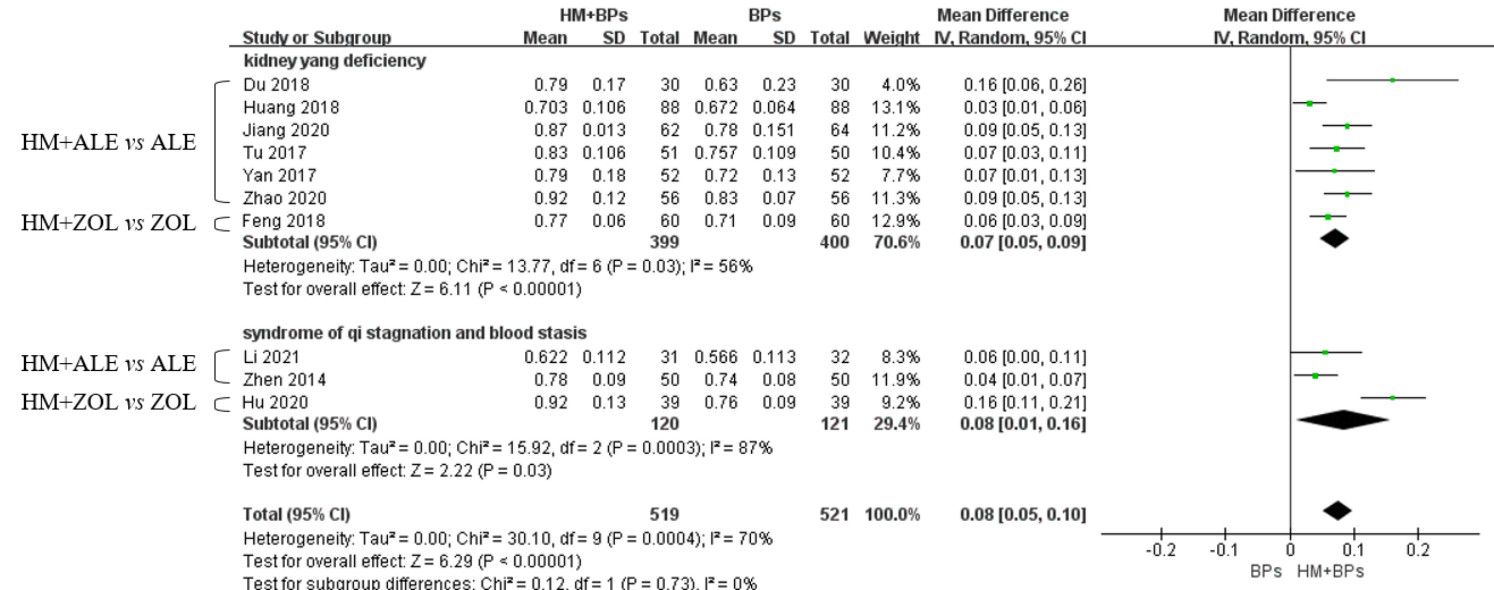
**

**Supplementary Figure S9.** The BMD improvement effects according to prescription pattern identification of HM and BPs at the femoral neck in PMOP (A) and Senile OP (B)

BMD, bone mineral density; HM, herbal medicine; BPs, bisphosphonates; ALE, alendronate; ZOL, zoledronate; PMOP, postmenopausal osteoporosis; SD, standard deviation; CI, confidence interval

## Supplementary Tables

**Supplementary Table S1a.** Search terms used in Core Databases

| Database | Search item |
| --- | --- |
| PubMed | (("plant extracts"[MeSH Terms]) OR ("herbal medicine"[MeSH Terms]) OR (herb*[Title/Abstract]) OR ("traditional chinese medicine"[Title/Abstract]) OR ("chinese medicine"[Title/Abstract]) OR ("korean medicine"[Title/Abstract]) OR ("traditional medicine"[Title/Abstract]) OR (TCM[Title/Abstract]) OR ("oriental medicine"[Title/Abstract]) OR ("plant extracts"[Title/Abstract]) OR (decoction[Title/Abstract]) OR (phytomedicine[Title/Abstract]) OR (botanical[Title/Abstract]) OR (plant[Title/Abstract]) OR (formula[Title/Abstract])) AND ((osteoporosis[MeSH Terms]) OR (osteoporo*[Title/Abstract]) OR ("bone loss"[Title/Abstract]) OR (osteopenia[Title/Abstract])) |
| Embase | ('herbal medicine'/exp OR herb*:ti,ab OR 'traditional chinese medicine':ti,ab OR 'chinese medicine':ti,ab OR 'korean medicine':ti,ab OR 'traditional medicine':ti,ab OR TCM:ti,ab OR 'oriental medicine':ti,ab OR 'plant extracts'/exp OR decoction:ti,ab OR phytomedicine:ti,ab OR botanical:ti,ab OR plant:ti,ab OR formula:ti,ab) AND ('osteoporosis'/exp OR osteoporo*:ti,ab OR 'bone loss':ti,ab OR osteopenia:ti,ab) |
| Cochrane Library | ((MeSH descriptor: [Herbal Medicine] explode all trees) OR (MeSH descriptor: [Plant Extracts] explode all trees) OR (herb*: ti,ab,kw) OR (traditional chinese medicine):ti,ab,kw) OR (chinese medicine):ti,ab,kw) OR (korean medicine):ti,ab,kw) OR (traditional medicine):ti,ab,kw) OR (TCM):ti,ab,kw) OR (oriental medicine):ti,ab,kw) OR (decoction):ti,ab,kw) OR (phytomedicine):ti,ab,kw) OR (botanical):ti,ab,kw) OR (plant):ti,ab,kw) OR (formula):ti,ab,kw)) AND ((MeSH descriptor: [Osteoporosis] explode all trees) OR (bone loss):ti,ab,kw) OR (osteopenia):ti,ab,kw)) |
| Google scholar | allintitle: (osteoporosis OR osteopenia) herb OR plant OR decoction OR formula OR "traditional*medicine" |

**Supplementary Table S1b.** Search terms used in China Databases

| Database | Search terms | |
| --- | --- | --- |
| China National Knowledge Infrastructure | 1 | (title + keyword + abstract = “草药” + “中医药” + “中药” + “中成药” + “方剂” + “方药” + “汤” + "胶囊") |
|  | 2 | (title + keyword + abstract = “骨质疏松” + "骨质疏松症" + “骨质减少” + “骨量减少”) |
|  | 3 | (title = “动物” + “实验” + “鼠” + “卵巢”) |
|  | 4 | (title = "体会" + "经验" + “理论” + “近况” + “总结” + “现状” + “展望” + “1例” + “2例” + “信息学” + “药效学“ + “共识” + “策略” + “1则” + “一则” + “2则”) |
|  | 5 | (title = “通知” + ”综述“ + "概述" + “述评” + “机制” + “机理” + “内涵” + “规律” + “回顾性” + “进展” + “发展” + “meta” + “指南” + “概况”) |
|  | 6 | (title = “诱发骨质疏松” + “病致骨质疏松” + “续发骨质疏松” + “合并骨质疏松” + “继发” + “糖尿病性” + “Ⅱ型” + “二型” + “强直性脊柱炎伴” + “类风湿关节炎伴”) |
|  | 7 | (full text = “骨密度” + “BMD”) |
|  | 8 | #1 AND #2 NOT #3 NOT #4 NOT #5 NOT #6 AND #7 |
| Wanfang Data | 1 | (title + keyword + abstract = “草药” or “中医药” or “中药” or “中成药” or “方剂” or “方药” or “汤” or “胶囊”) |
|  | 2 | (title + keyword + abstract = “骨质疏松” or “骨质疏松症” or “骨质减少” or “骨量减少”) |
|  | 3 | (title = “动物” or “实验” or “鼠” or “卵巢”) |
|  | 4 | (title = “体会” or “经验” or “理论” or “近况” or “总结” or “现状” or “展望” or “1例” or “2例” or “信息学” or “药效学“or “共识” or “策略” or “1则” or “一则” or “2则” or “通知” or “综述” or “概述” or “述评” or “机制” or “机理” or “内涵” or “规律” or “回顾性” or “进展” or “发展” or “meta” or “指南” or “概况”) |
|  | 5 | (title = “诱发骨质疏松” or “病致骨质疏松” or “续发骨质疏松” or “合并骨质疏松” or “继发” or “糖尿病性” or “Ⅱ型” or “二型” or “强直性脊柱炎伴” or “类风湿关节炎伴”) |
|  | 6 | (full text= “骨密度” or “BMD”) |
|  | 7 | #1 AND #2 NOT #3 NOT #4 NOT #5 AND #6 |

**Supplementary Table S1c.** Search terms used in Korea Databases

| Database | Search item |
| --- | --- |
| KISS | (골다공증\|골감소) AND (한약\|본초\|한의약\|탕약) |
| Kmbase | ([ALL=한약] OR [ALL=본초] OR [ALL=한의약] OR [ALL=탕약] OR [ALL=추출물]) AND ([ALL=골다공증] OR [ALL=골감소]) |
| ScienceOn | (골다공증 OR 골감소) AND (한약 OR 본초 OR 한의약 OR 탕약 OR 추출물) |
| Oasis | 골다공증\|골감소 |

**Supplementary Table S2.** The herbs composition of herbal medicines used in RCTs

| Study | Name (Type of formula,  N. of herbs) | Pharmaceutical producer | Extraction  process | Composition of herbal medicine (g) | Prescription pattern identification  (main effect,  additional effect) | Quality control report | Chemical analysis report |
| --- | --- | --- | --- | --- | --- | --- | --- |
| ***Postmenopausal osteoporosis (15 RCTs)*** | | | | | | | |
| Chen  2019 | Gushukang capsule  (Capsule, 5)  *: 8 capsules (2.56g)/d* | Liaoning Kangchen Pharmaceutical Co., Ltd. | Partially  reported^b^ | *Epimedium koreanum Nakai* [Berberidaceae; Epimedii H.] (uk)*, Rehmannia glutinosa (Gaertn.) DC.* [Orobanchaceae; Rehmanniae R. Recens] (uk)*, Drynaria fortunei (Kunze) J. Sm.* [Polypodiaceae; Drynariae Rh.] (uk)*, Astragalus membranaceus (Fisch.) Bunge* [Fabaceae; Astragali R.] (uk)*, Salvia miltiorrhiza Bunge* [Lamiaceae; Salviae Miltiorrhizae R. et Rh.] (uk) | Kidney yang deficiency | NR | NR |
| Chen  2021 | Modified Erxian decoction  (Decoction, 10)  *: 400ml/d* | NR | Partially  reported^a^ | *Cornus officinalis Siebold & Zuccarini* [Cornaceae; Corni F.] (10)*, Gynochthodes officinalis (F.C.How) Razafim. & B.Bremer* [Rubiaceae; Morindae Officinalis R.] (15)*, Epimedium koreanum Nakai* [Berberidaceae; Epimedii H.] (15)*, Rehmannia glutinosa (Gaertn.) DC.* [Orobanchaceae; Rehmanniae R. Recens] (30)*, Curculigo orchioides Gaertn.* [Hypoxidaceae; Curculiginis Rh.] (10)*, Phellodendron chinense C.K.Schneid.* [Rutaceae; Phellodendri Chinensis C.] (10)*, Glycyrrhiza glabra L.* [Fabaceae; Glycyrrhizae R. et Rh. Praeparata cum Melle] (6), *Ziziphus jujuba Mill.* [Rhamnaceae; Jujubae F.] (30), *Anemarrhena asphodeloides Bunge* [Liliaceae; Anemarrhenae Rh.] (15), *Angelica sinensis (Oliv.) Diels* [Apiaceae; Angelicae Sinensis R.] (5) | Kidney yang deficiency  (Liver-kidney yin deficiency, Blood deficiency, Fire of deficiency type) | NR | NR |
| Ju  2020 | Erxian decoction  (Decoction, 6)  *: uk/d* | Liaoning University of Traditional Chinese Medicine Hospital | Partially  reported^c^ | *Curculigo orchioides Gaertn.* [Hypoxidaceae; Curculiginis Rh.] (15), *Epimedium koreanum Nakai* [Berberidaceae; Epimedii H.] (15), *Gynochthodes officinalis (F.C.How) Razafim. & B.Bremer* [Rubiaceae; Morindae Officinalis R.] (10), *Angelica sinensis (Oliv.) Diels* [Apiaceae; Angelicae Sinensis R.] (10), *Phellodendron chinense C.K.Schneid.* [Rutaceae; Phellodendri Chinensis C.] (6), *Anemarrhena asphodeloides Bunge* [Liliaceae; Anemarrhenae Rh.] (6) | Kidney yang deficiency  (Fire of deficiency type, Syndrome of qi stagnation and blood stasis) | NR | NR |
| Liu  2016 | Erxian Bushen decoction  (Decoction, 12)  *: 400ml/d* | NR | Partially  reported^c^ | *Curculigo orchioides Gaertn.* [Hypoxidaceae; Curculiginis Rh.] (15), *Epimedium koreanum Nakai* [Berberidaceae; Epimedii H.] (15), *Rehmannia glutinosa (Gaertn.) DC.* [Orobanchaceae; Rehmanniae R. Recens] (12), *Dioscorea japonica Thunberg* [Dioscoreaceae; Dioscoreae Rh.] (12), *Cornus officinalis Siebold & Zuccarini* [Cornaceae; Corni F.] (10), *Lycium chinense Mill.* [Solanaceae; Lycii F.] (10), *Cervus nippon Temminck* [Cervidae; Cervi Cornus Colla] (10), *Cuscuta chinensis Lam.* [Convolvulaceae; Cuscutae S.] (10), *Eucommia ulmoides Oliv.* [Eucommiaceae; Eucommiae C.] (12), *Angelica sinensis (Oliv.) Diels* [Apiaceae; Angelicae Sinensis R.] (9), *Cinnamomum cassia Blume* [Lauraceae; Cinnamomi C.] (6), *Aconitum carmichaelii Debeaux* [Ranunculaceae; Aconiti Lateralis R. Praeparata] (6) | Kidney yang deficiency  (Liver-kidney yin deficiency) | NR | NR |
| Liu  2019 | Bushen Juanbi decoction  (Decoction, 11+α)  *: 400ml/d* | NR | Partially  reported^a^ | *Gynochthodes officinalis (F.C.How) Razafim. & B.Bremer* [Rubiaceae; Morindae Officinalis R.] (20), *Astragalus membranaceus (Fisch.) Bunge* [Fabaceae; Astragali R.] (30), *Epimedium koreanum Nakai* [Berberidaceae; Epimedii H.] (15), *Cuscuta chinensis Lam.* [Convolvulaceae; Cuscutae S.] (15), *Cullen corylifolium (L.) Medik.* [Fabaceae; Psoraleae F.] (15), *Lycium chinense Mill.* [Solanaceae; Lycii F.] (10), *Angelica sinensis (Oliv.) Diels* [Apiaceae; Angelicae Sinensis R.] (10), *Prunus persica (L.) Batsch* [Rosaceae; Persicae S.] (10), *Carthamus tinctorius Linné* [Asteraceae; Carthami Flos] (10), *Pteropus Stool* [Sciuridae; Trogopterorum Faeces] (10), *Glycyrrhiza uralensis Fisch. ex DC.* [Fabaceae; Glycyrrhizae R. et Rh.] (6) ; <Low back pain and lack of strength: Add *Angelica pubescens Maxim. f. biserrata Shan et Yuan* [Araliaceae; Angelicae Pubescentis R.] (15), *Taxillus chinensis (DC.) Danser* [Loranthaceae; Taxilli H.] (15)>; <Heat-damp: Add *Rehmannia glutinosa (Gaertn.) DC.* [Orobanchaceae; Rehmanniae R.] (15), *Eclipta prostrata Linné* [Asteraceae; Ecliptae H.] (15), *Ligustrum lucidum W.T.Aiton* [Oleaceae; Ligustri Lucidi F.] (15)>; <Limb fatigue: Add *Gentiana macrophylla Pall.* [Gentianaceae; Gentianae Macrophyllae R.] (10), *Angelica pubescens Maxim. f. biserrata Shan et Yuan* [Araliaceae; Angelicae Pubescentis R.] (10)>; <Chest-to-low back pain: Add *Bupleurum falcatum Linné* [Apiaceae; Bupleuri R.] (10), *Curcuma longa Linné* [Zingiberaceae; Curcumae R.] (10)>; <Blood stasis and night stabbing pain: Add *Buthus martensii Karsch* [Buthidae; Scorpio] (10), *Oldenlandia diffusa(Willd) Roxb.* [Rubiaceae; Hedyotidis Diffusae H.] (10), *Scolopendromorpha* [Scolopendridae; Scolopendra] (2)>; <Kidney yang deficiency: Add *Eucommia ulmoides Oliv.* [Eucommiaceae; Eucommiae C.] (15)>; <Kidney yin deficiency: Add *Rehmannia glutinosa (Gaertn.) DC.* [Orobanchaceae; Rehmanniae R. Recens] (10), *Anemarrhena asphodeloides Bunge* [Liliaceae; Anemarrhenae Rh.] (10)> | uk  (Kidney yang deficiency, Qi deficiency, Blood deficiency) | NR | NR |
| Lu  2016 | Kanggu zengsheng jiaonang  (Capsule, 9)  *: 15 capsules (uk)/d* | Jiangsu Kangyan Pharmaceutical Co., Ltd. | NR | *Rehmannia glutinosa (Gaertn.) DC.* [Orobanchaceae; Rehmanniae R. Recens] (uk), *Ligustrum lucidum W.T.Aiton* [Oleaceae; Ligustri Lucidi F.] (uk), *Cistanche deserticola Y. C. Ma* [Orobanchaceae; Cistanches H.] (uk), *Cibotium barometz (L.) J. Sm.* [Dicksoniaceae; Cibotii Rh.] (uk), *Drynaria fortunei (Kunze) J. Sm.* [Polypodiaceae; Drynariae Rh.] (uk), *Epimedium koreanum Nakai* [Berberidaceae; Epimedii H.] (uk), *Spatholobus suberectus Dunn* [Fabaceae; Spatholobi Caulis] (uk), *Achyranthes bidentata Blume* [Amaranthaceae; Achyranthis Bidentatae R.] (uk), *Raphanus raphanistrum subsp. sativus (L.) Domin* [Brassicaceae; Raphani S.] (uk) | Syndrome of qi stagnation and blood stasis  (Kidney yang deficiency, Liver-kidney yin deficiency) | NR | NR |
| Xiang  2013 | Duhuo Jisheng decoction  (Decoction, 14)  *: uk/d* | NR | NR | *Angelica pubescens Maxim. f. biserrata Shan et Yuan* [Araliaceae; Angelicae Pubescentis R.] (9), *Taxillus chinensis (DC.) Danser* [Loranthaceae; Taxilli H.] (6), *Eucommia ulmoides Oliv.* [Eucommiaceae; Eucommiae C.] (6), *Achyranthes bidentata Blume* [Amaranthaceae; Achyranthis Bidentatae R.] (6), *Asarum sieboldii Miq.* [Aristolochiaceae; Asari R. et Rh.] (6), *Gentiana macrophylla Pall.* [Gentianaceae; Gentianae Macrophyllae R.] (6), *Wolfiporia cocos (F.A. Wolf) Ryvarden & Gilb* [Polyporaceae; Poria cocos] (6), *Cinnamomum cassia Blume* [Lauraceae; Cinnamomi C.] (6), *Saposhnikovia divaricata (Turcz. ex Ledeb.) Schischk.* [Apiaceae; Saposhnikoviae R.] (6), *Ligusticum chuanxiong Hort.* [Apiaceae; Chuanxiong Rh.] (6), *Panax ginseng C.A.Mey.* [Araliaceae; Ginseng R. et Rh.] (6), Glycyrrhiza uralensis Fisch. ex DC. [Fabaceae; Glycyrrhizae R. et Rh.] (6), *Angelica sinensis (Oliv.) Diels* [Apiaceae; Angelicae Sinensis R.] (6), *Paeonia lactiflora Pall.* [Paeoniaceae; Paeoniae R.] (6), *Rehmannia glutinosa (Gaertn.) DC.* [Orobanchaceae; Rehmanniae R. Recens] (6) | uk  (Wind-dampness, Kidney yang deficiency, Blood deficiency, Syndrome of qi stagnation and blood stasis) | NR | NR |
| Xu  2009 | Xianlinggubao capsule  (Capsule, uk)  *: 9 capsules (uk)/d* | Sinopharm Group Tongjitang (Guizhou) Pharmaceuticals Co., Ltd. | NR | uk | Kidney yang deficiency | NR | NR |
| Xu  2010 | Qianggu capsule  (Capsule, uk)  *: 3 capsules (uk)/d* | Beijing Qihuang Pharmaceutical Co., Ltd. | NR | uk | Kidney yang deficiency | NR | NR |
| Xu  2019 | Wenshen Gushu recipe  (Decoction, 9)  *: 500ml/d* | Hainan Provincial Hospital of Traditional Chinese Medicine | Partially  reported^a^ | *Epimedium koreanum Nakai* [Berberidaceae; Epimedii H.] (20), *Rehmannia glutinosa (Gaertn.) DC.* [Orobanchaceae; Rehmanniae R. Recens] (20), *Codonopsis pilosula (Franch.) Nannf.* [Campanulaceae; Codonopsis R.] (10), *Drynaria fortunei (Kunze) J. Sm.* [Polypodiaceae; Drynariae Rh.] (10), *Cullen corylifolium (L.) Medik.* [Fabaceae; Psoraleae F.] (5), *Salvia miltiorrhiza Bunge* [Lamiaceae; Salviae Miltiorrhizae R. et Rh.] (10), *Wolfiporia cocos (F.A. Wolf) Ryvarden & Gilb* [Polyporaceae; Poria cocos] (10), *Lycium chinense Mill.* [Solanaceae; Lycii F.] (10), *Atractylodes macrocephala Koidz.* [Asteraceae; Atractylodis Macrocephalae Rh.] (10) | Kidney yang deficiency  (Liver-kidney yin deficiency) | NR | NR |
| Xue  2018 | Bushen Zhuanggu decoction  (Decoction, 3+α)  *: uk/d* | NR | Partially  reported^c^ | *Dipsacus asper Wall. ex Henry* [Dipsacaceae; Dipsaci R.] (20), *Drynaria fortunei (Kunze) J. Sm.* [Polypodiaceae; Drynariae Rh.] (20), *Eucommia ulmoides Oliv.* [Eucommiaceae; Eucommiae C.] (20); <Yang deficiency: Add *Aconitum carmichaelii Debeaux* [Ranunculaceae; Aconiti Lateralis R. Praeparata] (10), *Cinnamomum cassia Blume* [Lauraceae; Cinnamomi C.] (10)>; <Yin deficiency: Add *Paeonia lactiflora Pall.* [Paeoniaceae; Paeoniae R. Alba] (10), *Rehmannia glutinosa (Gaertn.) DC.* [Orobanchaceae; Rehmanniae R.] (10), *Anemarrhena asphodeloides Bunge* [Liliaceae; Anemarrhenae Rh.] (10)>; <Severe pain: Add *Ligusticum chuanxiong Hort.* [Apiaceae; Chuanxiong Rh.] (10), *Corydalis yanhusuo W. T. Wang* [Papaveraceae; Corydalis Rh.] (10), *Boswellia serrata Roxb.* [Burseraceae; Boswellia serrata] (5)] | Kidney yang deficiency | NR | NR |
| Yang 2017 | Duhuo Jisheng decoction  (Decoction, 15)  *: 300ml/d* | NR | Partially  reported^a^ | *Taxillus chinensis (DC.) Danser* [Loranthaceae; Taxilli H.] (20), *Lycium chinense Mill.* [Solanaceae; Lycii F.] (20), *Angelica pubescens Maxim. f. biserrata Shan et Yuan* [Araliaceae; Angelicae Pubescentis R.] (10), *Rehmannia glutinosa (Gaertn.) DC.* [Orobanchaceae; Rehmanniae R. Preparata] (10), *Cornus officinalis Siebold & Zuccarini* [Cornaceae; Corni F.] (10), *Chinemys reevesii Gray* [Geoemydidae; Colla Carapacis et Plastri] (10), *Cervus nippon Temminck* [Cervidae; Cervi Cornus Colla] (10), *Saposhnikovia divaricata (Turcz. ex Ledeb.) Schischk.* [Apiaceae; Saposhnikoviae R.] (10), *Eucommia ulmoides Oliv.* [Eucommiaceae; Eucommiae C.] (10), *Dipsacus asperoides C.Y.Cheng et T.M.Ai* [Dipsacaceae; Dipsaci Asperoidis R.] (10), *Achyranthes bidentata Blume* [Amaranthaceae; Achyranthis Bidentatae R.] (10), *Cuscuta chinensis Lam.* [Convolvulaceae; Cuscutae S.] (10), *Carthamus tinctorius Linné* [Asteraceae; Carthami Flos] (10), *Ligusticum chuanxiong Hort.* [Apiaceae; Chuanxiong Rh.] (10), *Glycyrrhiza uralensis Fisch. ex DC.* [Fabaceae; Glycyrrhizae R. et Rh.] (5) | Liver-kidney yin deficiency | NR | NR |
| Yang  2021 | Kuntai capsule  (Capsule, uk)  *: uk (6g)/d* | Guiyang Xintian Pharmaceutical Co., Ltd. | NR | uk | uk | NR | NR |
| Zhang  2015 | Liuwei Dihuang pill  (Pill, 12)  *: 2 pills (12g)/d* | Sichuan Provincial People's Hospital of Traditional Chinese Medicine | Partially  reported^a^ | *Rehmannia glutinosa (Gaertn.) DC.* [Orobanchaceae; Rehmanniae R. Recens] (160), *Cornus officinalis Siebold & Zuccarini* [Cornaceae; Corni F.] (80), *Paeonia × suffruticosa Andrews* [Paeoniaceae; Moutan C.] (60), *Dioscorea japonica Thunberg* [Dioscoreaceae; Dioscoreae Rh.] (80), *Wolfiporia cocos (F.A. Wolf) Ryvarden & Gilb* [Polyporaceae; Poria cocos] (60), *Alisma orientale Juzepczuk* [Alismataceae; Alismatis Rh.] (60), *Cervus nippon Temminck* [Cervidae; Cervi Cornus Colla] (60), *Chinemys reevesii Gray* [Geoemydidae; Colla Carapacis et Plastri] (60), *Lycium chinense Mill.* [Solanaceae; Lycii F.] (60), *Paeonia lactiflora Pall.* [Paeoniaceae; Paeoniae R. Alba] (30), *Cullen corylifolium (L.) Medik.* [Fabaceae; Psoraleae F.] (60), *Drynaria fortunei (Kunze) J. Sm.* [Polypodiaceae; Drynariae Rh.] (60) | Liver-kidney yin deficiency | NR | NR |
| Zhu  2020 | Yangxue Gushen decoction  (Decoction, 10)  *: 300ml/d* | Sinopharm Group Tongjitang (Guizhou) Pharmaceuticals Co., Ltd. | Partially  reported^a^ | *Taxillus chinensis (DC.) Danser* [Loranthaceae; Taxilli H.] (30), *Cibotium barometz (L.) J. Sm.* [Dicksoniaceae; Cibotii Rh.] (30), *Angelica sinensis (Oliv.) Diels* [Apiaceae; Angelicae Sinensis R.] (10), *Rehmannia glutinosa (Gaertn.) DC.* [Orobanchaceae; Rehmanniae R. Recens] (15), *Cornus officinalis Siebold & Zuccarini* [Cornaceae; Corni F.] (10), *Epimedium koreanum Nakai* [Berberidaceae; Epimedii H.] (15), *Gynochthodes officinalis (F.C.How) Razafim. & B.Bremer* [Rubiaceae; Morindae Officinalis R.] (15), *Cuscuta chinensis Lam.* [Convolvulaceae; Cuscutae S.] (15), *Eucommia ulmoides Oliv.* [Eucommiaceae; Eucommiae C.] (15), *Achyranthes bidentata Blume* [Amaranthaceae; Achyranthis Bidentatae R.] (10) | Kidney yang deficiency  (Liver-kidney yin deficiency, Blood deficiency, Syndrome of qi stagnation and blood stasis) | NR | NR |
| ***Senile osteoporosis (28 RCTs)*** | | | | | |  |  |
| Bao  2015 | Xianlinggubao capsule  (Capsule, 3+α)  *: 6 capsules (3g)/d* | Sinopharm Group Tongjitang (Guizhou) Pharmaceuticals Co., Ltd. | Partially  reported^b^ | *Cullen corylifolium (L.) Medik.* [Fabaceae; Psoraleae F.] (uk), *Epimedium koreanum Nakai* [Berberidaceae; Epimedii H.] (uk), *Dipsacus asper Wall. ex Henry* [Dipsacaceae; Dipsaci R.] (uk), etc. | Kidney yang deficiency | NR | NR |
| Bu  2021 | Bushen Huoxue decoction  (Decoction, 19+α)  *: 1 dose (uk)/d* | NR | Partially  reported^c^ | *Astragalus membranaceus (Fisch.) Bunge* [Fabaceae; Astragali R.] (30), *Corydalis yanhusuo W. T. Wang* [Papaveraceae; Corydalis Rh.] (20), *Rehmannia glutinosa (Gaertn.) DC.* [Orobanchaceae; Rehmanniae R. Recens] (15), *Lycium chinense Mill.* [Solanaceae; Lycii F.] (15), *Eucommia ulmoides Oliv.* [Eucommiaceae; Eucommiae C.] (15), *Cistanche deserticola Y. C. Ma* [Orobanchaceae; Cistanches H.] (15), *Angelica sinensis (Oliv.) Diels* [Apiaceae; Angelicae Sinensis R.] (15), *Carthamus tinctorius Linné* [Asteraceae; Carthami Flos] (15), *Drynaria fortunei (Kunze) J. Sm.* [Polypodiaceae; Drynariae Rh.] (15), *Salvia miltiorrhiza Bunge* [Lamiaceae; Salviae Miltiorrhizae R. et Rh.] (15), *Lycopus lucidus Turcz. ex Benth.* [Lamiaceae; Lycopi H.] (15), *Angelica pubescens Maxim. f. biserrata Shan et Yuan* [Araliaceae; Angelicae Pubescentis R.] (12), *Cornus officinalis Siebold & Zuccarini* [Cornaceae; Corni F.] (10), *Cuscuta chinensis Lam.* [Convolvulaceae; Cuscutae S.] (10), *Cullen corylifolium (L.) Medik.* [Fabaceae; Psoraleae F.] (10), *Biancaea sappan (L.) Tod.* [Fabaceae; Sappan Lignum] (9), *Citrus reticulata Blanco* [Rutaceae; Citri Reticulatae Pericarpium] (8), *Glycyrrhiza uralensis Fisch. ex DC.* [Fabaceae; Glycyrrhizae R. et Rh.] (6), *Commiphora myrrha (T.Nees) Engl.* [Burseraceae; Myrrh] (6); <Blood stasis: Add *Prunus persica (L.) Batsch* [Rosaceae; Persicae S.] (15)>; <Swelling: Add *Plantago asiatica L.* [Aginaceae; Plantaginis S.] (15), *Acanthopanax gracilistylus W. W. Smith* [Araliaceae; Acanthopanacis C.] (15), *Lycopus lucidus Turcz. ex Benth.* [Lamiaceae; Lycopi H.] (10)>; <Spleen deficiency: Add *Atractylodes macrocephala Koidz.* [Asteraceae; Atractylodis Macrocephalae Rh.] (15), *Codonopsis pilosula (Franch.) Nannf.* [Campanulaceae; Codonopsis R.] (20)>; <Constipation: Add *Trichosanthes rosthornii Harms* [Cucurbitaceae; Trichosanthis F.] (15)>; <Dry mouth: Add *Trichosanthes rosthornii Harms* [Cucurbitaceae; Trichosanthis R.] (15)>; <Vexation and irritability: Add *Curcuma longa Linné* [Zingiberaceae; Curcumae R.] (20), *Bupleurum falcatum Linné* [Apiaceae; Bupleuri R.]  (15)>; <Soreness of the waist and knees: Add *Achyranthes bidentata Blume* [Amaranthaceae; Achyranthis Bidentatae R.] (15), *Taxillus chinensis (DC.) Danser* [Loranthaceae; Taxilli H.] (15), *Dipsacus asperoides C.Y.Cheng et T.M.Ai* [Dipsacaceae; Dipsaci Asperoidis R.] (10)> | uk  (Syndrome of qi stagnation and blood stasis, Liver-kidney yin deficiency) | NR | NR |
| Chen  2015 | Xianlinggubao capsule  (Capsule, 3+α)  *: 6 capsules (uk)/d* | Sinopharm Group Tongjitang (Guizhou) Pharmaceuticals Co., Ltd. | NR | *Cullen corylifolium (L.) Medik.* [Fabaceae; Psoraleae F.] (uk), *Salvia miltiorrhiza Bunge* [Lamiaceae; Salviae Miltiorrhizae R. et Rh.] (uk), *Epimedium koreanum Nakai* [Berberidaceae; Epimedii H.] (uk), etc. | Kidney yang deficiency | NR | NR |
| Chu  2021 | Astragalus L&K Tonifying decoction  (Decoction, 11)  *: 1 dose (uk)/d* | Shunde Hospital of Guangzhou University of Chinese Medicine | NR | *Astragalus membranaceus (Fisch.) Bunge* [Fabaceae; Astragali R.] (30), *Angelica pubescens Maxim. f. biserrata Shan et Yuan* [Araliaceae; Angelicae Pubescentis R.] (20), *Taxillus chinensis (DC.) Danser* [Loranthaceae; Taxilli H.] (20), *Achyranthes bidentata Blume* [Amaranthaceae; Achyranthis Bidentatae R.] (10), *Ligusticum chuanxiong Hort.* [Apiaceae; Chuanxiong Rh.] (10), *Eucommia ulmoides Oliv.* [Eucommiaceae; Eucommiae C.] (10), *Gentiana macrophylla Pall.* [Gentianaceae; Gentianae Macrophyllae R.] (10), *Saposhnikovia divaricata (Turcz. ex Ledeb.) Schischk.* [Apiaceae; Saposhnikoviae R.] (10), *Angelica sinensis (Oliv.) Diels* [Apiaceae; Angelicae Sinensis R.] (10), *Paeonia lactiflora Pall.* [Paeoniaceae; Paeoniae R. Rubra] (10), *Rehmannia glutinosa (Gaertn.) DC.* [Orobanchaceae; Rehmanniae R. Recens] (10) | uk  (Qi deficiency, Syndrome of qi stagnation and blood stasis, Wind-dampness) | NR | NR |
| Cui  2020 | Gukangfang granules  (Granule, 10+α)  *: 40g/d* | NR | Partially  reported^c^ | *Astragalus membranaceus (Fisch.) Bunge* [Fabaceae; Astragali R.] (20), *Angelica sinensis (Oliv.) Diels* [Apiaceae; Angelicae Sinensis R.] (10), *Salvia miltiorrhiza Bunge* [Lamiaceae; Salviae Miltiorrhizae R. et Rh.] (10), *Atractylodes macrocephala Koidz.* [Asteraceae; Atractylodis Macrocephalae Rh.] (15), *Rehmannia glutinosa (Gaertn.) DC.* [Orobanchaceae; Rehmanniae R. Recens] (15), *Cullen corylifolium (L.) Medik.* [Fabaceae; Psoraleae F.] (30), *Epimedium koreanum Nakai* [Berberidaceae; Epimedii H.] (15), *Reynoutria multiflora (Thunb.) Moldenke* [Polygonaceae; Polygoni Multiflori R.] (15), *Paeonia lactiflora Pall.* [Paeoniaceae; Paeoniae R.] (15), *Ziziphus jujuba Mill.* [Rhamnaceae; Jujubae F.] (10), etc. | uk  (Kidney yang deficiency, Qi deficiency, Liver-kidney yin deficiency, Syndrome of qi stagnation and blood stasis) | NR | NR |
| Du  2018 | Xianlinggubao capsule  (Capsule, 6+α)  *: 6 capsules (uk)/d* | Sinopharm Group Tongjitang (Guizhou) Pharmaceuticals Co., Ltd. | NR | *Salvia miltiorrhiza Bunge* [Lamiaceae; Salviae Miltiorrhizae R. et Rh.] (uk), *Rehmannia glutinosa (Gaertn.) DC.* [Orobanchaceae; Rehmanniae R.] (uk), *Epimedium koreanum Nakai* [Berberidaceae; Epimedii H.] (uk), *Cullen corylifolium (L.) Medik.* [Fabaceae; Psoraleae F.] (uk), *Anemarrhena asphodeloides Bunge* [Liliaceae; Anemarrhenae Rh.] (uk), *Dipsacus asper Wall. ex Henry* [Dipsacaceae; Dipsaci R.] (uk), etc. | Kidney yang deficiency | NR | NR |
| Feng  2018 | Tenghuang Jiangu capsules  (Capsule, 7)  *: 8 capsules (2g)/d* | Gansu Xifeng Pharmaceutical Co., Ltd. | NR | *Rehmannia glutinosa (Gaertn.) DC.* [Orobanchaceae; Rehmanniae R. Recens] (uk), *Pyrola calliantha Andres* [Ericaceae; Pyrolae H.] (uk), *Drynaria fortunei (Kunze) J. Sm.* [Polypodiaceae; Drynariae Rh.] (uk), *Cistanche deserticola Y. C. Ma* [Orobanchaceae; Cistanches H.] (uk), *Epimedium koreanum Nakai* [Berberidaceae; Epimedii H.] (uk), *Spatholobus suberectus Dunn* [Fabaceae; Spatholobi Caulis] (uk), *Raphanus raphanistrum subsp. sativus (L.) Domin* [Brassicaceae; Raphani S.] (uk) | Kidney yang deficiency | NR | NR |
| Fu  2016 | Xianlinggubao capsule  (Capsule, 6)  *: 6 capsules (3g)/d* | Sinopharm Group Tongjitang (Guizhou) Pharmaceuticals Co., Ltd. | NR | *Epimedium koreanum Nakai* [Berberidaceae; Epimedii H.] (uk), *Dipsacus asper Wall. ex Henry* [Dipsacaceae; Dipsaci R.] (uk), *Salvia miltiorrhiza Bunge* [Lamiaceae; Salviae Miltiorrhizae R. et Rh.] (uk), *Anemarrhena asphodeloides Bunge* [Liliaceae; Anemarrhenae Rh.] (uk), *Cullen corylifolium (L.) Medik.* [Fabaceae; Psoraleae F.] (uk), *Rehmannia glutinosa (Gaertn.) DC.* [Orobanchaceae; Rehmanniae R.] (uk) | Kidney yang deficiency | NR | NR |
| Gui  2017 | Bushen Yiqi Huayu decoction  (Decoction, 20)  *: 1 dose (uk)/d* | NR | Partially  reported^c^ | *Flemingia philippinensis Merr. & Rolfe* [Fabaceae; Flemingia philippinensis] (uk), *Rehmannia glutinosa (Gaertn.) DC.* [Orobanchaceae; Rehmanniae R. Recens] (uk), *Lycium chinense Mill.* [Solanaceae; Lycii F.] (uk), *Aconitum carmichaelii Debeaux* [Ranunculaceae; Aconiti Lateralis R. Praeparata] (uk), *Taxillus chinensis (DC.) Danser* [Loranthaceae; Taxilli H.] (uk), *Epimedium koreanum Nakai* [Berberidaceae; Epimedii H.] (uk), *Codonopsis pilosula (Franch.) Nannf.* [Campanulaceae; Codonopsis R.] (uk), *Eucommia ulmoides Oliv.* [Eucommiaceae; Eucommiae C.] (uk), *Drynaria fortunei (Kunze) J. Sm.* [Polypodiaceae; Drynariae Rh.] (uk), *Angelica sinensis (Oliv.) Diels* [Apiaceae; Angelicae Sinensis R.] (uk), *Atractylodes macrocephala Koidz.* [Asteraceae; Atractylodis Macrocephalae Rh.] (uk), *Astragalus membranaceus (Fisch.) Bunge* [Fabaceae; Astragali R.] (uk), *Achyranthes bidentata Blume* [Amaranthaceae; Achyranthis Bidentatae R.] (uk) etc. | Kidney yang deficiency  (Liver-kidney yin deficiency, Qi deficiency, Syndrome of qi stagnation and blood stasis) | NR | NR |
| Guo  2020 | Xianlinggubao capsule  (Capsule, uk)  *: uk (3g)/d* | Sinopharm Group Tongjitang (Guizhou) Pharmaceuticals Co., Ltd. | NR | uk | Kidney yang deficiency | NR | NR |
| Hu  2020 | Modified Bushen Huoxue decoction  (Decoction, 19+α)  *: 1 dose (uk)/d* | Jinan city hospital of Traditional Chinese Medicine | Partially  reported^c^ | *Rehmannia glutinosa (Gaertn.) DC.* [Orobanchaceae; Rehmanniae R. Recens] (15), *Cornus officinalis Siebold & Zuccarini* [Cornaceae; Corni F.] (10), *Lycium chinense Mill.* [Solanaceae; Lycii F.] (15), *Cuscuta chinensis Lam.* [Convolvulaceae; Cuscutae S.] (10), *Cullen corylifolium (L.) Medik.* [Fabaceae; Psoraleae F.] (10), *Eucommia ulmoides Oliv.* [Eucommiaceae; Eucommiae C.] (15), *Angelica pubescens Maxim. f. biserrata Shan et Yuan* [Araliaceae; Angelicae Pubescentis R.] (12), *Cistanche deserticola Y. C. Ma* [Orobanchaceae; Cistanches H.] (15), *Angelica sinensis (Oliv.) Diels* [Apiaceae; Angelicae Sinensis R.] (15), *Commiphora myrrha (T.Nees) Engl.* [Burseraceae; Myrrh] (6), *Carthamus tinctorius Linné* [Asteraceae; Carthami Flos] (15), *Drynaria fortunei (Kunze) J. Sm.* [Polypodiaceae; Drynariae Rh.] (15), *Biancaea sappan (L.) Tod.* [Fabaceae; Sappan Lignum] (9), *Salvia miltiorrhiza Bunge* [Lamiaceae; Salviae Miltiorrhizae R. et Rh.] (15), *Lycopus lucidus Turcz. ex Benth.* [Lamiaceae; Lycopi H.] (15), *Corydalis yanhusuo W. T. Wang* [Papaveraceae; Corydalis Rh.] (20), *Astragalus membranaceus (Fisch.) Bunge* [Fabaceae; Astragali R.] (30), *Citrus reticulata Blanco* [Rutaceae; Citri Reticulatae Pericarpium] (8), *Glycyrrhiza uralensis Fisch. ex DC.* [Fabaceae; Glycyrrhizae R. et Rh.] (6); <Swelling: Add *Acanthopanax gracilistylus W. W. Smith* [Araliaceae; Acanthopanacis C.] (15), *Plantago asiatica L.* [Aginaceae; Plantaginis S.] (15), *Lycopus lucidus Turcz. ex Benth.* [Lamiaceae; Lycopi H.] (10)>; <Blood stasis: Add *Prunus persica (L.) Batsch* [Rosaceae; Persicae S.] (15)>; <Spleen deficiency: Add *Codonopsis pilosula (Franch.) Nannf.* [Campanulaceae; Codonopsis R.] (20), *Atractylodes macrocephala Koidz.* [Asteraceae; Atractylodis Macrocephalae Rh.] (15)>; <Constipation: Add *Trichosanthes rosthornii Harms* [Cucurbitaceae; Trichosanthis F.] (15)>; <Dry mouth: Add *Trichosanthes rosthornii Harms* [Cucurbitaceae; Trichosanthis R.] (15)>; <Vexation and irritability: Add *Bupleurum falcatum Linné* [Apiaceae; Bupleuri R.] (15), *Curcuma longa Linné* [Zingiberaceae; Curcumae R.] (20)>; <Soreness of the waist and knees: Add *Dipsacus asperoides C.Y.Cheng et T.M.Ai* [Dipsacaceae; Dipsaci Asperoidis R.] (10), *Taxillus chinensis (DC.) Danser* [Loranthaceae; Taxilli H.] (15), *Achyranthes bidentata Blume* [Amaranthaceae; Achyranthis Bidentatae R.] (15)> | Syndrome of qi stagnation and blood stasis  (Kidney yang deficiency, Qi deficiency, Liver-kidney yin deficiency) | NR | NR |
| Huang 2018 | Bushentang  (Decoction, 6)  *: 1 dose (200ml)/d* | NR | Partially  reported^a^ | *Cistanche deserticola Y. C. Ma* [Orobanchaceae; Cistanches H.] (20), *Astragalus complanatus R. Br.* [Leguminosae; Astragali Complanati S.] (15), *Cullen corylifolium (L.) Medik.* [Fabaceae; Psoraleae F.] (15), *Polygonatum sibiricum Redouté* [Asparagaceae; Polygonati Rh.] (10), *Lilium lancifolium Thunb.* [Liliaceae; Lilii Bulbus] (10), *Ilex cornuta Lindl. & Paxton* [Aquifoliaceae; Ilicis Cornutae Folium] (10) | Kidney yang deficiency | NR | NR |
| Jiang  2020 | Lujiao Zhuanggu capsule  (Capsule, 4)  *: uk (4.05g)/d* | NR | NR | *Cervus nippon Temminck* [Cervidae; Cervi Cornu Degelatinatum] (uk), *Gynochthodes officinalis (F.C.How) Razafim. & B.Bremer* [Rubiaceae; Morindae Officinalis R.] (uk), *Drynaria fortunei (Kunze) J. Sm.* [Polypodiaceae; Drynariae Rh.] (uk), *Cullen corylifolium (L.) Medik.* [Fabaceae; Psoraleae F.] (uk) | Kidney yang deficiency | NR | NR |
| Kang  2020 | Xianlinggubao capsule  (Capsule, uk)  *: 6 capsules (3g)/d* | Sinopharm Group Tongjitang (Guizhou) Pharmaceuticals Co., Ltd. | NR | uk | Kidney yang deficiency | NR | NR |
| Li  2019 | Xianlinggubao capsule  (Capsule, 6)  *: 6 capsules (uk)/d* | Sinopharm Group Tongjitang (Guizhou) Pharmaceuticals Co., Ltd. | NR | *Epimedium koreanum Nakai* [Berberidaceae; Epimedii H.] (uk), *Dipsacus asper Wall. ex Henry* [Dipsacaceae; Dipsaci R.] (uk), *Salvia miltiorrhiza Bunge* [Lamiaceae; Salviae Miltiorrhizae R. et Rh.] (uk), *Anemarrhena asphodeloides Bunge* [Liliaceae; Anemarrhenae Rh.] (uk), *Cullen corylifolium (L.) Medik.* [Fabaceae; Psoraleae F.] (uk), *Rehmannia glutinosa (Gaertn.) DC.* [Orobanchaceae; Rehmanniae R.] (uk) | Kidney yang deficiency | NR | NR |
| Li  2021 | Biqi capsule  (Capsule, 5+α)  *: 8 capsules (2.4g)/d* | Tianjin Darentang Jingwanhong Pharmaceutical Co., Ltd. | NR | *Strychnos nux-vomica L.* [Loganiaceae; Strychni Semen] (uk), *Earthworm* [Megascolecidae; Pheretima aspergillum] (uk), *Salvia miltiorrhiza Bunge* [Lamiaceae; Salviae Miltiorrhizae R. et Rh.] (uk), *Wolfiporia cocos (F.A. Wolf) Ryvarden & Gilb* [Polyporaceae; Poria cocos] (uk), *Achyranthes bidentata Blume* [Amaranthaceae; Achyranthis Bidentatae R.] (uk), etc. | Syndrome of qi stagnation and blood stasis | NR | NR |
| Shen  2017 | Bushen Huoxue decoction  (Decoction, 16+α)  *: 1 dose (800ml)/d* | NR | NR | *Prunus persica (L.) Batsch* [Rosaceae; Persicae S.] (12), *Carthamus tinctorius Linné* [Asteraceae; Carthami Flos] (12), *Rehmannia glutinosa (Gaertn.) DC.* [Orobanchaceae; Rehmanniae R. Recens] (30), *Angelica sinensis (Oliv.) Diels* [Apiaceae; Angelicae Sinensis R.] (12), *Ligusticum chuanxiong Hort.* [Apiaceae; Chuanxiong Rh.] (15), *Salvia miltiorrhiza Bunge* [Lamiaceae; Salviae Miltiorrhizae R. et Rh.] (12), *Epimedium koreanum Nakai* [Berberidaceae; Epimedii H.] (15), *Eucommia ulmoides Oliv.* [Eucommiaceae; Eucommiae C.] (15), *Dipsacus asper Wall. ex Henry* [Dipsacaceae; Dipsaci R.] (15), *Cervus nippon Temminck* [Cervidae; Cervi Cornus Colla] (15), *Cornus officinalis Siebold & Zuccarini* [Cornaceae; Corni F.] (20), *Cuscuta chinensis Lam.* [Convolvulaceae; Cuscutae S.] (20), *Achyranthes bidentata Blume* [Amaranthaceae; Achyranthis Bidentatae R.] (15), *Astragalus membranaceus (Fisch.) Bunge* [Fabaceae; Astragali R.] (30), *Dioscorea japonica Thunberg* [Dioscoreaceae; Dioscoreae Rh.] (30), *Corydalis yanhusuo W. T. Wang* [Papaveraceae; Corydalis Rh.] (12), etc. | Syndrome of qi stagnation and blood stasis  (Blood deficiency, Kidney yang deficiency) | NR | NR |
| Tu  2017 | Compound Epimedium  oral liquid  (Decoction, 8)  *: 1 dose (30ml)/d* | Shandong FuPai Pharmaceutical Co., Ltd. | NR | *Epimedium koreanum Nakai* [Berberidaceae; Epimedii H.] (uk), *Rehmannia glutinosa (Gaertn.) DC.* [Orobanchaceae; Rehmanniae R. Recens] (uk), *Dioscorea japonica Thunberg* [Dioscoreaceae; Dioscoreae Rh.] (uk), *Lycium chinense Mill.* [Solanaceae; Lycii F.] (uk), *Cornus officinalis Siebold & Zuccarini* [Cornaceae; Corni F.] (uk), *Eucommia ulmoides Oliv.* [Eucommiaceae; Eucommiae C.] (uk), *Cinnamomum cassia Blume* [Lauraceae; Cinnamomi C.] (uk), *Glycyrrhiza uralensis Fisch. ex DC.* [Fabaceae; Glycyrrhizae R. et Rh.] (uk) | Kidney yang deficiency  (Liver-kidney yin deficiency) | NR | NR |
| Wang  2018 | Guli Jiaonang capsule  (Capsule, 6+α)  *: 9 capsules (2.7g)/d* | Guizhou Bailing Group Pharmaceutical Co., Ltd. | NR | *Epimedium koreanum Nakai* [Berberidaceae; Epimedii H.] (uk), *Pueraria montana var. lobata (Willd.) Maesen & S.M.Almeida ex Sanjappa & Predeep* [Fabaceae; Puerariae Lobatae R.] (uk), *Clematis chinensis Osbeck* [Ranunculaceae; Clematidis R. et Rh.] (uk), *Chaenomeles speciosa (Sweet) Nakai* [Rosaceae; Chaenomelis F.] (uk), *Achyranthes bidentata Blume* [Amaranthaceae; Achyranthis Bidentatae R.] (uk), *Cullen corylifolium (L.) Medik.* [Fabaceae; Psoraleae F.] (uk), etc. | Kidney yang deficiency  (Wind-dampness) | NR | NR |
| Wang  2020 | Tonifying Q&K decoction  (Decoction, 12)  *: 1 dose (uk)/d* | NR | NR | *Rehmannia glutinosa (Gaertn.) DC.* [Orobanchaceae; Rehmanniae R. Recens] (15), *Hemerocallis citrina Baroni* [Asphodelaceae; Hemerocallis citrina] (15), *Cibotium barometz (L.) J. Sm.* [Dicksoniaceae; Cibotii Rh.] (15), *Epimedium koreanum Nakai* [Berberidaceae; Epimedii H.] (15), *Atractylodes macrocephala Koidz.* [Asteraceae; Atractylodis Macrocephalae Rh.] (15), *Drynaria fortunei (Kunze) J. Sm.* [Polypodiaceae; Drynariae Rh.] (15), *Achyranthes bidentata Blume* [Amaranthaceae; Achyranthis Bidentatae R.] (10), *Angelica sinensis (Oliv.) Diels* [Apiaceae; Angelicae Sinensis R.] (10), *Panax notoginseng (Burkill) F.H.Chen* [Araliaceae; NotoGinseng Radix et Rhizoma] (10), *Cistanche deserticola Y. C. Ma* [Orobanchaceae; Cistanches H.] (10), *Codonopsis pilosula (Franch.) Nannf.* [Campanulaceae; Codonopsis R.] (10), *Glycyrrhiza uralensis Fisch. ex DC.* [Fabaceae; Glycyrrhizae R. et Rh.] (6) | Syndrome of qi stagnation and blood stasis  (Kidney yang deficiency, Liver-kidney yin deficiency) | NR | NR |
| Xie  2015 | Duhuo Jisheng decoction  (Decoction, 12)  *: 1 dose (uk)/d* | NR | NR | *Angelica pubescens Maxim. f. biserrata Shan et Yuan* [Araliaceae; Angelicae Pubescentis R.] (15), *Taxillus chinensis (DC.) Danser* [Loranthaceae; Taxilli H.] (20), *Epimedium koreanum Nakai* [Berberidaceae; Epimedii H.] (10), *Achyranthes bidentata Blume* [Amaranthaceae; Achyranthis Bidentatae R.] (15), *Angelica sinensis (Oliv.) Diels* [Apiaceae; Angelicae Sinensis R.] (10), *Rehmannia glutinosa (Gaertn.) DC.* [Orobanchaceae; Rehmanniae R. Recens] (20), *Astragalus membranaceus (Fisch.) Bunge* [Fabaceae; Astragali R.] (30), *Ligusticum chuanxiong Hort.* [Apiaceae; Chuanxiong Rh.] (10), *Eucommia ulmoides Oliv.* [Eucommiaceae; Eucommiae C.] (15), *Gentiana macrophylla Pall.* [Gentianaceae; Gentianae Macrophyllae R.] (15), *Panax ginseng C.A.Mey.* [Araliaceae; Ginseng R. et Rh.] (10), *Glycyrrhiza uralensis Fisch. ex DC.* [Fabaceae; Glycyrrhizae R. et Rh.] (6) | uk  (Wind-dampness, Qi deficiency, Kidney yang deficiency, Blood deficiency, Syndrome of qi stagnation and blood stasis) | NR | NR |
| Yan  2017 | Increased densities decoction  (Decoction, 12)  *: 1 dose (400ml)/d* | NR | Partially reported^b^ | *Epimedium koreanum Nakai* [Berberidaceae; Epimedii H.] (30), *Rehmannia glutinosa (Gaertn.) DC.* [Orobanchaceae; Rehmanniae R. Recens] (20), *Drynaria fortunei (Kunze) J. Sm.* [Polypodiaceae; Drynariae Rh.] (30), *Lycium chinense Mill.* [Solanaceae; Lycii F.] (15), *Chaenomeles speciosa (Sweet) Nakai* [Rosaceae; Chaenomelis F.] (15), *Dipsacus asper Wall. ex Henry* [Dipsacaceae; Dipsaci R.] (10), *Angelica sinensis (Oliv.) Diels* [Apiaceae; Angelicae Sinensis R.] (15), *Clematis chinensis Osbeck* [Ranunculaceae; Clematidis R. et Rh.] (15), *Atractylodes macrocephala Koidz.* [Asteraceae; Atractylodis Macrocephalae Rh.] (10), *Wolfiporia cocos (F.A. Wolf) Ryvarden & Gilb* [Polyporaceae; Poria cocos] (10), *Glycyrrhiza uralensis Fisch. ex DC.* [Fabaceae; Glycyrrhizae R. et Rh.] (6), *Acanthopanax gracilistylus W. W. Smith* [Araliaceae; Acanthopanacis C.] (6) | Kidney yang deficiency  (Liver-kidney yin deficiency, Wind-dampness) | NR | NR |
| Zeng  2015 | Xianlinggubao capsule  (Capsule, 6)  *: 6 capsules (3g)/d* | Sinopharm Group Tongjitang (Guizhou) Pharmaceuticals Co., Ltd. | NR | *Rehmannia glutinosa (Gaertn.) DC.* [Orobanchaceae; Rehmanniae R.] (uk), *Cullen corylifolium (L.) Medik.* [Fabaceae; Psoraleae F.] (uk), *Anemarrhena asphodeloides Bunge* [Liliaceae; Anemarrhenae Rh.] (uk), *Salvia miltiorrhiza Bunge* [Lamiaceae; Salviae Miltiorrhizae R. et Rh.] (uk), *Dipsacus asper Wall. ex Henry* [Dipsacaceae; Dipsaci R.] (uk), *Epimedium koreanum Nakai* [Berberidaceae; Epimedii H.] (uk) | Kidney yang deficiency | NR | NR |
| Zhang  2014 | Xianlinggubao capsule  (Capsule, 5)  *: 6 capsules (uk)/d* | Sinopharm Group Tongjitang (Guizhou) Pharmaceuticals Co., Ltd. | NR | *Salvia miltiorrhiza Bunge* [Lamiaceae; Salviae Miltiorrhizae R. et Rh.] (uk), *Dipsacus asper Wall. ex Henry* [Dipsacaceae; Dipsaci R.] (uk), *Anemarrhena asphodeloides Bunge* [Liliaceae; Anemarrhenae Rh.] (uk), *Epimedium koreanum Nakai* [Berberidaceae; Epimedii H.] (uk), *Cullen corylifolium (L.) Medik.* [Fabaceae; Psoraleae F.] (uk) | Kidney yang deficiency | NR | NR |
| Zhang  2019 | Yishen Zhuanggu decoction  (Decoction, 14+α)  *: 1 dose (350ml)/d* | NR | NR | *Drynaria fortunei (Kunze) J. Sm.* [Polypodiaceae; Drynariae Rh.] (20), *Cornus officinalis Siebold & Zuccarini* [Cornaceae; Corni F.] (15), *Eucommia ulmoides Oliv.* [Eucommiaceae; Eucommiae C.] (15), *Spatholobus suberectus Dunn* [Fabaceae; Spatholobi Caulis] (30), *Cibotium barometz (L.) J. Sm.* [Dicksoniaceae; Cibotii Rh.] (8), *Angelica sinensis (Oliv.) Diels* [Apiaceae; Angelicae Sinensis R.] (12), *Cuscuta chinensis Lam.* [Convolvulaceae; Cuscutae S.] (10), *Dioscorea japonica Thunberg* [Dioscoreaceae; Dioscoreae Rh.] (12), *Saposhnikovia divaricata (Turcz. ex Ledeb.) Schischk.* [Apiaceae; Saposhnikoviae R.] (10), *Ligusticum chuanxiong Hort.* [Apiaceae; Chuanxiong Rh.] (12), *Reynoutria multiflora (Thunb.) Moldenke* [Polygonaceae; Polygoni Multiflori R.] (15), *Citrus reticulata Blanco* [Rutaceae; Citri Reticulatae Pericarpium] (10), *Juglans regia L.* [Juglandaceae; Juglandis S.] (10), *Glycyrrhiza uralensis Fisch. ex DC. [Fabaceae; Glycyrrhizae R. et Rh.]* (6); <Blood stasis: Add *Prunus persica (L.) Batsch* [Rosaceae; Persicae S.] (12), *Salvia miltiorrhiza Bunge* [Lamiaceae; Salviae Miltiorrhizae R. et Rh.] (12)>; <Yang deficiency: Add *Cervus nippon Temminck*[Cervidae; Cervi Cornus Colla] (15), *Panax ginseng C.A.Mey.* [Araliaceae; Ginseng R. et Rh. Rubra (12)>; <Lack of strength: Add *Astragalus membranaceus (Fisch.) Bunge* [Fabaceae; Astragali R. Praeparata] (15) *Codonopsis pilosula (Franch.) Nannf.* [Campanulaceae; Codonopsis R.] (15)> | uk  (Kidney yang deficiency, Liver-kidney yin deficiency, Syndrome of qi stagnation and blood stasis) | NR | NR |
| Zhang  2021 | uk  (Decoction,14+α)  *: 1 dose (600ml)/d* | NR | Partially reported^b^ | *Rehmannia glutinosa (Gaertn.) DC.* [Orobanchaceae; Rehmanniae R. Recens] (20), *Eucommia ulmoides Oliv.* [Eucommiaceae; Eucommiae C.] (15), *Epimedium koreanum Nakai* [Berberidaceae; Epimedii H.] (20), *Reynoutria multiflora (Thunb.) Moldenke* [Polygonaceae; Polygoni Multiflori R.] (10), *Cuscuta chinensis Lam.* [Convolvulaceae; Cuscutae S.] (12), *Drynaria fortunei (Kunze) J. Sm.* [Polypodiaceae; Drynariae Rh.] (12), *Cullen corylifolium (L.) Medik.* [Fabaceae; Psoraleae F.] (12), *Curculigo orchioides Gaertn.* [Hypoxidaceae; Curculiginis Rh.] (9), *Gynochthodes officinalis (F.C.How) Razafim. & B.Bremer* [Rubiaceae; Morindae Officinalis R.] (9), *Astragalus membranaceus (Fisch.) Bunge* [Fabaceae; Astragali R.] (15), *Lycium chinense Mill.* [Solanaceae; Lycii F.] (12), *Angelica sinensis (Oliv.) Diels* [Apiaceae; Angelicae Sinensis R.] (10), *Paeonia lactiflora Pall.* [Paeoniaceae; Paeoniae R. Alba] (10), *Glycyrrhiza uralensis Fisch. ex DC.* [Fabaceae; Glycyrrhizae R. et Rh.] (9); <Spleen-kidney yang deficiency: Add *Codonopsis pilosula (Franch.) Nannf.* [Campanulaceae; Codonopsis R.] (9), *Dioscorea japonica Thunberg* [Dioscoreaceae; Dioscoreae Rh.] (9)>; <Liver-kidney yin deficiency: Add *Polygonatum sibiricum Redouté* [Asparagaceae; Polygonati Rh.] (12), *Morus alba L.* [Moraceae; Mori F.] (9), *Cervus nippon Temminck* [Cervidae; Cervi Cornu Pantotrichum] (6)>; <Syndrome of qi stagnation and blood stasis: Add *Prunus persica (L.) Batsch* [Rosaceae; Persicae S.] (9), *Corydalis yanhusuo W. T. Wang* [Papaveraceae; Corydalis Rh.] (6)> | uk  (Liver-kidney yin deficiency, Kidney yang deficiency) | NR | NR |
| Zhao  2020 | Xianlinggubao capsule  (Capsule, 6)  *: 6 capsules (3g)/d* | Sinopharm Group Tongjitang (Guizhou) Pharmaceuticals Co., Ltd. | NR | *Rehmannia glutinosa (Gaertn.) DC.* [Orobanchaceae; Rehmanniae R.] (uk), *Salvia miltiorrhiza Bunge* [Lamiaceae; Salviae Miltiorrhizae R. et Rh.] (uk), *Epimedium koreanum Nakai* [Berberidaceae; Epimedii H.] (uk), *Cullen corylifolium (L.) Medik.* [Fabaceae; Psoraleae F.] (uk), *Anemarrhena asphodeloides Bunge* [Liliaceae; Anemarrhenae Rh.] (uk), *Dipsacus asper Wall. ex Henry* [Dipsacaceae; Dipsaci R.] (uk) | Kidney yang deficiency | NR | NR |
| Zhen  2014 | Gubi decoction  (Decoction, 13)  *: 1 dose (300ml)/d* | NR | Partially reported^a^ | *Rehmannia glutinosa (Gaertn.) DC.* [Orobanchaceae; Rehmanniae R. Recens] (15), *Eucommia ulmoides Oliv.* [Eucommiaceae; Eucommiae C.] (15), *Acanthopanax gracilistylus W. W. Smith* [Araliaceae; Acanthopanacis C.] (15), *Salvia miltiorrhiza Bunge* [Lamiaceae; Salviae Miltiorrhizae R. et Rh.] (12), *Epimedium koreanum Nakai* [Berberidaceae; Epimedii H.] (10), *Drynaria fortunei (Kunze) J. Sm.* [Polypodiaceae; Drynariae Rh.] (10), *Atractylodes macrocephala Koidz.* [Asteraceae; Atractylodis Macrocephalae Rh.] (10), *Dioscorea japonica Thunberg* [Dioscoreaceae; Dioscoreae Rh.] (10), *Cyperus rotundus Linné* [Cyperaceae; Cyperi Rh.] (10), *Paeonia lactiflora Pall.* [Paeoniaceae; Paeoniae R. Alba] (10), Glycyrrhiza glabra L. [Fabaceae; Glycyrrhizae R. et Rh. Praeparata cum Melle] (10), *Eupolyphaga sinensis* [Corydiidae; Eupolyphaga Steleophaga] (8), *Earthworm* [Megascolecidae; Pheretima aspergillum] (8) | Syndrome of qi stagnation and blood stasis  (Kidney yang deficiency, Liver-kidney yin deficiency) | NR | NR |
| N., number; uk, unknown; d, day; NR, not reported; L&K, liver and kidney; Q&K, qi and kidney; R., radix; Rh., Rhizoma; C., Cortex; S., Semen; H., Herba; F., Fructus  ^a^: The amount of the provoked extract and the extraction process were reported.  ^b^: Only the amount of the provoked extract was reported.  ^c^: Only the extraction process was reported. | | | | | | | |

**Supplementary Table S3.** Herbal medicine and its active compounds

| Herbal Medicine | Active compounds  (Effective in bone metabolism) | Effect of bone cells | |
| --- | --- | --- | --- |
|  |  | Osteoblast | Osteoclast |
| Xianlinggubao capsule | Quercetin^[1]^  Luteolin^[2][3]^  Kaempferol^[4]^  Anhydroicaritin^[5]^  Diosgenin^[6][7]^ | Promote  Promote  Promote  Promote | Inhibit  Inhibit  Inhibit  Inhibit  Inhibit |
| Biqi capsule | Brucine^[8]^  Liquiritin^[9]^  Salvianolic acid B^[10]^  Glycyrrhizic acid^[11]^  Cryptotanshinone^[12][13]^  Tanshinone IIA^[14]^ | Promote  Promote | Inhibit  Inhibit  Inhibit  Inhibit  Inhibit  Inhibit |
| Gushukang capsule | Tanshinol^[15][16]^  Icariin^[17][18]^  Naringin^[19][20]^  Isoquercitrin^[21][22]^  Tanshinone IIA^[23]^ | Promote  Promote  Promote  Promote  Promote | Inhibit  Inhibit  Inhibit  Inhibit  Inhibit |
| Kuntai capsule | Baicalein^[24][25]^  Wogonin^[26]^  Berberine^[27][28]^  Magnoflorine^[29][30]^ | Promote  Promote  Promote | Inhibit  Inhibit  Inhibit  Inhibit |
| Liuwei Dihuang pill | Gallic acid^[31][32]^  5-hydroxymethyl furfural^[33]^  Morroniside^[34]^  Paeoniflorin^[35]^  Sweroside^[36]^ | Promote  Promote  Promote  Promote | Inhibit  Inhibit  Inhibit  Inhibit |
| Qianggu capsule | Naringenin^[37][38]^ | Promote | Inhibit |
| Tenghuang Jiangu capsules | Quercetin^[39]^  Luteolin^[40][41]^  Kaempferol^[42]^ | Promote  Promote  Ptomote | Inhibit  Inhibit  Inhibit |
| [1] Satué, M., et al. (2013). "Quercitrin and taxifolin stimulate osteoblast differentiation in MC3T3-E1 cells and inhibit osteoclastogenesis in RAW 264.7 cells." Biochemical Pharmacology 86(10): 1476-1486.  [2] Kim, T.-H., et al. (2011). "The effects of luteolin on osteoclast differentiation, function in vitro and ovariectomy-induced bone loss." The Journal of nutritional biochemistry 22(1): 8-15.  [3] Nash, L. A., et al. (2015). "Rooibos flavonoids, orientin and luteolin, stimulate mineralization in human osteoblasts through the Wnt pathway." Molecular nutrition & food research 59(3): 443-453.  [4] Wong, S. K., et al. (2019). "The osteoprotective effects of kaempferol: the evidence from in vivo and in vitro studies." Drug design, development and therapy: 3497-3514.  [5] Zheng, Z.-G., et al. (2017). "Anhydroicaritin, a SREBPs inhibitor, inhibits RANKL-induced osteoclastic differentiation and improves diabetic osteoporosis in STZ-induced mice." European journal of pharmacology 809: 156-162.  [6] Alcantara, E. H., et al. (2011). "Diosgenin stimulates osteogenic activity by increasing bone matrix protein synthesis and bone-specific transcription factor Runx2 in osteoblastic MC3T3-E1 cells." The Journal of nutritional biochemistry 22(11): 1055-1063.  [7] Zhang, Z., et al. (2018). "Diosgenin protects against alveolar bone loss in ovariectomized rats via regulating long non‑coding RNAs." Experimental and therapeutic medicine 16(5): 3939-3950.  [8] Hu, K.-f., et al. (2017). "Brucine inhibits bone metastasis of breast cancer cells by suppressing Jagged1/Notch1 signaling pathways." Chinese journal of integrative medicine 23: 110-116.  [9] Hong, G., et al. (2023). "A novel Glycyrrhiza glabra extract liquiritin targeting NFATc1 activity and ROS levels to counteract ovariectomy-induced osteoporosis and bone loss in murine model." Frontiers in Pharmacology 14: 1287827.  [10] Zhang, X., et al. (2017). "Salvianolic acid B and danshensu induce osteogenic differentiation of rat bone marrow stromal stem cells by upregulating the nitric oxide pathway." Experimental and therapeutic medicine 14(4): 2779-2788.  [11] Yin, Z., et al. (2019). "Glycyrrhizic acid suppresses osteoclast differentiation and postmenopausal osteoporosis by modulating the NF-κB, ERK, and JNK signaling pathways." European journal of pharmacology 859: 172550.  [12] Wang, W., et al. (2019). "Cryptotanshinone inhibits RANKL‐induced osteoclastogenesis by regulating ERK and NF‐κB signaling pathways." Journal of cellular biochemistry 120(5): 7333-7340.  [13] Yang, W., et al. (2022). "Cryptotanshinone Suppressed Postmenopausal Osteoporosis by Preventing RANKL‐Mediated Osteoclastogenesis against Kidney Injury." Evidence‐Based Complementary and Alternative Medicine 2022(1): 2821984.  [14] Zhu, S., et al. (2018). "Tanshinone‑IIA attenuates the deleterious effects of oxidative stress in osteoporosis through the NF‑κB signaling pathway." Molecular Medicine Reports 17(5): 6969-6976.  [15] Han, J. and W. Wang (2017). "Effects of tanshinol on markers of bone turnover in ovariectomized rats and osteoblast cultures." PLoS ONE 12(7): e0181175.  [16] Lai, W., et al. (2021). "Tanshinol alleviates microcirculation disturbance and impaired bone formation by attenuating TXNIP signaling in GIO rats." Frontiers in Pharmacology 12: 722175.  [17] Hsieh, T.-P., et al. (2011). "Icariin inhibits osteoclast differentiation and bone resorption by suppression of MAPKs/NF-κB regulated HIF-1α and PGE2 synthesis." Phytomedicine 18(2-3): 176-185.  [18] Song, L., et al. (2013). "Icariin induces osteoblast proliferation, differentiation and mineralization through estrogen receptor-mediated ERK and JNK signal activation." European journal of pharmacology 714(1-3): 15-22.  [19] Hirata, M., et al. (2009). "Naringin suppresses osteoclast formation and enhances bone mass in mice." Journal of Health Science 55(3): 463-467.  [20] Wu, G.-J., et al. (2021). "Naringin improves osteoblast mineralization and bone healing and strength through regulating estrogen receptor alpha-dependent alkaline phosphatase gene expression." Journal of Agricultural and Food Chemistry 69(44): 13020-13033.  [21] Li, M., et al. (2019). "Isoquercitrin promotes the osteogenic differentiation of osteoblasts and BMSCs via the RUNX2 or BMP pathway." Connective Tissue Research 60(2): 189-199.  [22] Liu, Y., et al. (2024). "Isoquercitrin attenuates the osteoclast-mediated bone loss in rheumatoid arthritis via the Nrf2/ROS/NF-κB pathway." Biochimica et Biophysica Acta (BBA)-Molecular Basis of Disease 1870(2): 166977.  [23] Zhu, S., et al. (2018). "Tanshinone‑IIA attenuates the deleterious effects of oxidative stress in osteoporosis through the NF‑κB signaling pathway." Molecular Medicine Reports 17(5): 6969-6976.  [24] Kim, J. M., et al. (2008). "Baicalein stimulates osteoblast differentiation via coordinating activation of MAP kinases and transcription factors." Journal of cellular biochemistry 104(5): 1906-1917.  [25] Kim, M. H., et al. (2008). "Baicalein inhibits osteoclast differentiation and induces mature osteoclast apoptosis." Food and Chemical Toxicology 46(11): 3375-3382.  [26] Geng, X., et al. (2015). "Wogonin inhibits osteoclast differentiation by inhibiting NFATc1 translocation into the nucleus." Experimental and therapeutic medicine 10(3): 1066-1070.  [27] Hu, J.-P., et al. (2008). "Berberine inhibits RANKL-induced osteoclast formation and survival through suppressing the NF-κB and Akt pathways." European journal of pharmacology 580(1-2): 70-79.  [28] Nam, S. W., et al. (2020). "Discovery and development of berberine derivatives as stimulants of osteoblast differentiation." Biochemical and Biophysical Research Communications 527(1): 110-116.  [29] Cai, Z., et al. (2018). "Magnoflorine with hyaluronic acid gel promotes subchondral bone regeneration and attenuates cartilage degeneration in early osteoarthritis." Bone 116: 266-278.  [30] Sun, Z., et al. (2020). "Magnoflorine suppresses MAPK and NF-κB signaling to prevent inflammatory osteolysis induced by titanium particles in vivo and osteoclastogenesis via RANKL in vitro." Frontiers in Pharmacology 11: 389.  [31] Zhang, P., et al. (2022). "Gallic acid inhibits osteoclastogenesis and prevents ovariectomy-induced bone loss." Frontiers in Endocrinology 13: 963237.  [32] Pham, T. H., et al. (2024). "Gallic acid induces osteoblast differentiation and alleviates inflammatory response through GPR35/GSK3β/β‐catenin signaling pathway in human periodontal ligament cells." Journal of periodontal research 59(1): 204-219.  [33] Tan, X.-l., et al. (2014). "5-(Hydroxymethyl)-2-furaldehyde inhibits adipogenic and enhances osteogenic differentiation of rat bone mesenchymal stem cells." Natural Product Communications 9(4): 1934578X1400900427.  [34] Lee, C. G., et al. (2021). "Anti-osteoporotic effect of morroniside on osteoblast and osteoclast differentiation in vitro and ovariectomized mice in vivo." International Journal of Molecular Sciences 22(19): 10642.  [35] Wang, Y., et al. (2018). "Paeoniflorin regulates osteoclastogenesis and osteoblastogenesis via manipulating NF-κB signaling pathway both in vitro and in vivo." Oncotarget 9(7): 7372.  [36] Wu, Q.-C., et al. (2020). "Sweroside promotes osteoblastic differentiation and mineralization via interaction of membrane estrogen receptor-α and GPR30 mediated p38 signalling pathway on MC3T3-E1 cells." Phytomedicine 68: 153146.  [37] Hirata, M., et al. (2009). "Naringin suppresses osteoclast formation and enhances bone mass in mice." Journal of Health Science 55(3): 463-467.  [38] Wu, G.-J., et al. (2021). "Naringin improves osteoblast mineralization and bone healing and strength through regulating estrogen receptor alpha-dependent alkaline phosphatase gene expression." Journal of Agricultural and Food Chemistry 69(44): 13020-13033.  [39] Satué, M., et al. (2013). "Quercitrin and taxifolin stimulate osteoblast differentiation in MC3T3-E1 cells and inhibit osteoclastogenesis in RAW 264.7 cells." Biochemical Pharmacology 86(10): 1476-1486.  [40] Kim, T.-H., et al. (2011). "The effects of luteolin on osteoclast differentiation, function in vitro and ovariectomy-induced bone loss." The Journal of nutritional biochemistry 22(1): 8-15.  [41] Nash, L. A., et al. (2015). "Rooibos flavonoids, orientin and luteolin, stimulate mineralization in human osteoblasts through the Wnt pathway." Molecular nutrition & food research 59(3): 443-453.  [42] Wong, S. K., et al. (2019). "The osteoprotective effects of kaempferol: the evidence from in vivo and in vitro studies." Drug design, development and therapy: 3497-3514. | | | |

**Supplementary Table S4.** Sensitivity analysis of effects of HM plus BPs on BMD improvement

| BMD site  (OP type) | Study omitted | Pooled estimate | 95% CI | *p* value | I^2^(%) |
| --- | --- | --- | --- | --- | --- |
| Lumbar  spine  (PMOP) | **< Total >** | 0.10 | 0.05 - 0.15 | 0.0002 | 97 |
|  | **Chen 2019** | 0.11 | 0.05 - 0.16 | 0.0003 | 97 |
|  | **Ju 2020** | 0.10 | 0.05 - 0.16 | 0.0003 | 97 |
|  | **Liu 2016** | 0.11 | 0.05 - 0.16 | 0.0002 | 97 |
|  | **Liu 2019** | 0.09 | 0.04 - 0.15 | 0.0007 | 97 |
|  | **Lu 2016** | 0.11 | 0.05 - 0.16 | 0.0002 | 97 |
|  | **Xiang 2013** | 0.08 | 0.05 - 0.11 | <0.00001 | 86 |
|  | **Xu 2009** | 0.10 | 0.05 - 0.16 | 0.0003 | 97 |
|  | **Xu 2010** | 0.10 | 0.05 - 0.16 | 0.0003 | 97 |
|  | **Xue 2018** | 0.10 | 0.04 - 0.16 | 0.001 | 97 |
|  | **Yang 2017** | 0.10 | 0.05 - 0.15 | 0.0003 | 97 |
|  | **Zhang 2015** | 0.11 | 0.05 - 0.16 | 0.0001 | 97 |
|  | **Zhu 2020** | 0.10 | 0.04 - 0.15 | 0.0007 | 97 |
|  | **<Sub group 1> HMs *plus* ALE** | 0.16 | -0.09 - 0.41 | 0.22 | 100 |
|  | **<Sub group 2> HMs *plus* ZOL** | 0.09 | 0.06 - 0.11 | <0.00001 | 85 |
| Lumbar  spine  (Senile OP) | **< Total >** | 0.09 | 0.08 - 0.11 | <0.00001 | 81 |
|  | **Bao 2015** | 0.10 | 0.08 - 0.11 | <0.00001 | 78 |
|  | **Bu 2021** | 0.09 | 0.07 - 0.10 | <0.00001 | 73 |
|  | **Chu 2021** | 0.09 | 0.08 - 0.11 | <0.00001 | 82 |
|  | **Cui 2020** | 0.10 | 0.08 - 0.11 | <0.00001 | 81 |
|  | **Du 2018** | 0.09 | 0.07 - 0.11 | <0.00001 | 82 |
|  | **Feng 2018** | 0.09 | 0.08 - 0.11 | <0.00001 | 81 |
|  | **Fu 2016** | 0.09 | 0.08 - 0.11 | <0.00001 | 82 |
|  | **Gui 2017** | 0.09 | 0.08 - 0.11 | <0.00001 | 82 |
|  | **Hu 2020** | 0.09 | 0.07 - 0.11 | <0.00001 | 81 |
|  | **Huang 2018** | 0.10 | 0.08 - 0.11 | <0.00001 | 80 |
|  | **Jiang 2020** | 0.09 | 0.07 - 0.11 | <0.00001 | 81 |
|  | **Li 2019** | 0.09 | 0.07 - 0.11 | <0.00001 | 81 |
|  | **Tu 2017** | 0.09 | 0.07 - 0.11 | <0.00001 | 82 |
|  | **Wang 2018** | 0.09 | 0.07 - 0.11 | <0.00001 | 82 |
|  | **Wang 2020** | 0.09 | 0.07 - 0.11 | <0.00001 | 81 |
|  | **Yan 2017** | 0.09 | 0.07 - 0.11 | <0.00001 | 82 |
|  | **Zhang 2014** | 0.09 | 0.08 - 0.11 | <0.00001 | 81 |
|  | **Zhang 2019** | 0.09 | 0.07 - 0.11 | <0.00001 | 80 |
|  | **Zhang 2021** | 0.09 | 0.07 - 0.11 | <0.00001 | 81 |
|  | **Zhao 2020** | 0.09 | 0.07 - 0.11 | <0.00001 | 82 |
|  | **Zhen 2014** | 0.10 | 0.08 - 0.11 | <0.00001 | 80 |
|  | **<Sub group 1> HMs *plus* ALE** | 0.13 | 0.05 - 0.20 | 0.001 | 92 |
|  | **<Sub group 2> HMs *plus* ZOL** | 0.09 | 0.07 - 0.10 | <0.00001 | 72 |
| Femoral neck  (PMOP) | **<Total>** | 0.06 | 0.04 - 0.08 | <0.00001 | 62 |
|  | **Ju 2020** | 0.06 | 0.03 - 0.08 | <0.00001 | 65 |
|  | **Liu 2016** | 0.06 | 0.04 - 0.08 | <0.00001 | 50 |
|  | **Liu 2019** | 0.05 | 0.03 - 0.07 | <0.00001 | 48 |
|  | **Lu 2016** | 0.06 | 0.04 - 0.09 | <0.00001 | 67 |
|  | **Xu 2010** | 0.06 | 0.04 - 0.09 | <0.00001 | 66 |
|  | **Xu 2019** | 0.06 | 0.04 - 0.09 | <0.00001 | 67 |
|  | **Xue 2018** | 0.05 | 0.03 - 0.08 | <0.00001 | 59 |
|  | **Yang 2017** | 0.06 | 0.03 - 0.08 | <0.00001 | 64 |
| Femoral neck  (Senile OP) | **<Total>** | 0.09 | 0.05 - 0.14 | <0.0001 | 95 |
|  | **Bu 2021** | 0.08 | 0.06 - 0.10 | <0.00001 | 72 |
|  | **Du 2018** | 0.09 | 0.04 - 0.14 | 0.0002 | 96 |
|  | **Feng 2018** | 0.10 | 0.05 - 0.15 | <0.0001 | 95 |
|  | **Hu 2020** | 0.09 | 0.04 - 0.14 | 0.0003 | 96 |
|  | **Huang 2018** | 0.10 | 0.06 - 0.15 | <0.0001 | 94 |
|  | **Jiang 2020** | 0.10 | 0.05 - 0.14 | 0.0001 | 96 |
|  | **Li 2021** | 0.10 | 0.05 - 0.15 | <0.0001 | 96 |
|  | **Tu 2017** | 0.10 | 0.05 - 0.14 | <0.0001 | 96 |
|  | **Yan 2017** | 0.10 | 0.05 - 0.14 | <0.0001 | 96 |
|  | **Zhang 2019** | 0.09 | 0.04 - 0.14 | 0.0002 | 96 |
|  | **Zhao 2020** | 0.10 | 0.05 - 0.14 | 0.0001 | 96 |
|  | **Zhen 2014** | 0.10 | 0.05 - 0.15 | <0.0001 | 95 |
|  | **<Sub group 1> HMs *plus* ALE** | 0.14 | 0.04 - 0.24 | 0.006 | 98 |
|  | **<Sub group 2> HMs *plus* ZOL** | 0.07 | 0.05 - 0.10 | <0.00001 | 65 |

**Supplementary Table S5.** Sensitivity analysis of effects of HM plus ALE on BMD improvement

| BMD site | Study omitted | Pooled estimate | 95% CI | *p* value | I^2^(%) |
| --- | --- | --- | --- | --- | --- |
| Lumbar  spine | <Total> | 0.09 | 0.07 - 0.10 | <0.00001 | 78 |
|  | **Bao 2015** | 0.09 | 0.07 - 0.10 | <0.00001 | 76 |
|  | **Chen 2019** | 0.09 | 0.07 - 0.10 | <0.00001 | 77 |
|  | **Chu 2021** | 0.09 | 0.07 - 0.10 | <0.00001 | 79 |
|  | **Cui 2020** | 0.09 | 0.07 - 0.10 | <0.00001 | 78 |
|  | **Du 2018** | 0.09 | 0.07 - 0.10 | <0.00001 | 79 |
|  | **Fu 2016** | 0.09 | 0.07 - 0.10 | <0.00001 | 79 |
|  | **Gui 2017** | 0.09 | 0.07 - 0.10 | <0.00001 | 79 |
|  | **Huang 2018** | 0.09 | 0.07 - 0.10 | <0.00001 | 78 |
|  | **Jiang 2020** | 0.09 | 0.07 - 0.10 | <0.00001 | 79 |
|  | **Ju 2020** | 0.09 | 0.07 - 0.10 | <0.00001 | 79 |
|  | **Li 2019** | 0.08 | 0.07 - 0.10 | <0.00001 | 77 |
|  | **Liu 2016** | 0.09 | 0.07 - 0.10 | <0.00001 | 77 |
|  | **Liu 2019** | 0.08 | 0.07 - 0.10 | <0.00001 | 78 |
|  | **Lu 2016** | 0.09 | 0.07 - 0.10 | <0.00001 | 78 |
|  | **Tu 2017** | 0.08 | 0.07 - 0.10 | <0.00001 | 79 |
|  | **Wang 2018** | 0.08 | 0.07 - 0.10 | <0.00001 | 79 |
|  | **Wang 2020** | 0.09 | 0.07 - 0.10 | <0.00001 | 78 |
|  | **Xu 2009** | 0.09 | 0.07 - 0.10 | <0.00001 | 79 |
|  | **Xu 2010** | 0.09 | 0.07 - 0.10 | <0.00001 | 79 |
|  | **Xue 2018** | 0.08 | 0.07 - 0.10 | <0.00001 | 74 |
|  | **Yan 2017** | 0.09 | 0.07 - 0.10 | <0.00001 | 79 |
|  | **Yang 2017** | 0.09 | 0.07 - 0.10 | <0.00001 | 79 |
|  | **Zhang 2014** | 0.09 | 0.07 - 0.10 | <0.00001 | 79 |
|  | **Zhang 2019** | 0.08 | 0.07 - 0.10 | <0.00001 | 77 |
|  | **Zhang 2021** | 0.08 | 0.07 - 0.10 | <0.00001 | 78 |
|  | **Zhao 2020** | 0.09 | 0.07 - 0.10 | <0.00001 | 79 |
|  | **Zhen 2014** | 0.09 | 0.07 - 0.10 | <0.00001 | 78 |
|  | **Zhu 2020** | 0.08 | 0.07 - 0.10 | <0.00001 | 77 |
| FFemoral neck | **<Total>** | 0.07 | 0.05 - 0.08 | <0.00001 | 64 |
|  | **Du 2018** | 0.06 | 0.05 - 0.08 | <0.00001 | 63 |
|  | **Huang 2018** | 0.07 | 0.05 - 0.09 | <0.00001 | 62 |
|  | **Jiang 2020** | 0.06 | 0.05 - 0.08 | <0.00001 | 64 |
|  | **Ju 2020** | 0.07 | 0.05 - 0.08 | <0.00001 | 66 |
|  | **Li 2021** | 0.07 | 0.05 - 0.08 | <0.00001 | 66 |
|  | **Liu 2016** | 0.07 | 0.05 - 0.09 | <0.00001 | 58 |
|  | **Liu 2019** | 0.06 | 0.05 - 0.08 | <0.00001 | 61 |
|  | **Lu 2016** | 0.07 | 0.05 - 0.09 | <0.00001 | 66 |
|  | **Tu 2017** | 0.07 | 0.05 - 0.08 | <0.00001 | 66 |
|  | **Xu 2010** | 0.07 | 0.05 - 0.09 | <0.00001 | 65 |
|  | **Xu 2019** | 0.07 | 0.05 - 0.09 | <0.00001 | 66 |
|  | **Xue 2018** | 0.07 | 0.05 - 0.08 | <0.00001 | 65 |
|  | **Yan 2017** | 0.07 | 0.05 - 0.08 | <0.00001 | 66 |
|  | **Yang 2017** | 0.07 | 0.05 - 0.08 | <0.00001 | 66 |
|  | **Zhang 2019** | 0.06 | 0.05 - 0.08 | <0.00001 | 57 |
|  | **Zhao 2020** | 0.06 | 0.05 - 0.08 | <0.00001 | 64 |
|  | **Zhen 2014** | 0.07 | 0.05 - 0.09 | <0.00001 | 65 |

**Supplementary Table S6.** Sensitivity analysis of effects of HM plus ALE on BMD improvement in PMOP

| Sub-group  (BMD site) | Study omitted | Pooled estimate | 95% CI | *p* value | I^2^(%) |
| --- | --- | --- | --- | --- | --- |
| Total (Lumbar spine) | **Chen 2019** | 0.09 | 0.06 - 0.12 | <0.00001 | 84 |
|  | **Ju 2020** | 0.09 | 0.06 - 0.12 | <0.00001 | 87 |
|  | **Liu 2016** | 0.09 | 0.06 - 0.12 | <0.00001 | 84 |
|  | **Liu 2019** | 0.08 | 0.05 - 0.11 | <0.00001 | 85 |
|  | **Lu 2016** | 0.09 | 0.06 - 0.12 | <0.00001 | 86 |
|  | **Xu 2009** | 0.09 | 0.06 - 0.12 | <0.00001 | 87 |
|  | **Xu 2010** | 0.09 | 0.06 - 0.12 | <0.00001 | 87 |
|  | **Xue 2018** | 0.08 | 0.05 - 0.10 | <0.00001 | 76 |
|  | **Yang 2017** | 0.08 | 0.05 - 0.11 | <0.00001 | 87 |
|  | **Zhu 2020** | 0.08 | 0.05 - 0.11 | <0.00001 | 84 |
| Treatment  for 3 months  (Lumbar spine) | **Liu 2019** | 0.13 | 0.11 - 0.15 | <0.00001 | 0 |
|  | **Xue 2018** | 0.14 | 0.11 - 0.17 | <0.00001 | 0 |
|  | **Yang 2017** | 0.13 | 0.12 - 0.15 | <0.00001 | 0 |
|  | **Zhu 2020** | 0.13 | 0.11 - 0.15 | <0.00001 | 0 |
| Treatment  for 6 months  (Lumbar spine) | **Chen 2019** | 0.06 | 0.04 - 0.07 | <0.00001 | 0 |
|  | **Ju 2020** | 0.05 | 0.04 - 0.06 | <0.00001 | 0 |
|  | **Liu 2016** | 0.06 | 0.04 - 0.07 | <0.00001 | 0 |
|  | **Lu 2016** | 0.05 | 0.04 - 0.07 | <0.00001 | 0 |
|  | **Xu 2009** | 0.05 | 0.04 - 0.06 | <0.00001 | 0 |
|  | **Xu 2010** | 0.05 | 0.04 - 0.06 | <0.00001 | 0 |
| Total (Femoral neck) | **Ju 2020** | 0.06 | 0.03 - 0.08 | <0.00001 | 65 |
|  | **Liu 2016** | 0.06 | 0.04 - 0.08 | <0.00001 | 50 |
|  | **Liu 2019** | 0.05 | 0.03 - 0.07 | <0.00001 | 48 |
|  | **Lu 2016** | 0.06 | 0.04 - 0.09 | <0.00001 | 67 |
|  | **Xu 2010** | 0.06 | 0.04 - 0.09 | <0.00001 | 66 |
|  | **Xu 2019** | 0.06 | 0.04 - 0.09 | <0.00001 | 67 |
|  | **Xue 2018** | 0.05 | 0.03 - 0.08 | <0.00001 | 59 |
|  | **Yang 2017** | 0.06 | 0.03 - 0.08 | <0.00001 | 64 |
| Treatment  for < 3 months  (Femoral neck) | **Liu 2019** | 0.07 | 0.04 - 0.10 | <0.00001 | 28 |
|  | **Xu 2019** | 0.10 | 0.07 - 0.13 | <0.00001 | 0 |
|  | **Xue 2018** | 0.09 | 0.03 - 0.15 | 0.003 | 68 |
|  | **Yang 2017** | 0.09 | 0.04 - 0.14 | 0.0007 | 69 |
| Treatment  for 6 months  (Femoral neck) | **Ju 2020** | 0.04 | 0.02 - 0.05 | <0.0001 | 18 |
|  | **Liu 2016** | 0.05 | 0.03 - 0.07 | <0.00001 | 0 |
|  | **Lu 2016** | 0.04 | 0.01 - 0.07 | 0.003 | 61 |
|  | **Xu 2010** | 0.04 | 0.01 - 0.07 | 0.004 | 62 |

**Supplementary Table S7.** Sensitivity analysis of effects of HM plus ALE on BMD improvement in Senile OP

| Sub-group  (BMD site) | Study omitted | Pooled estimate | 95% CI | *p* value | I^2^(%) |
| --- | --- | --- | --- | --- | --- |
| Total  (Lumbar spine) | **Bao 2015** | 0.09 | 0.07 - 0.10 | <0.00001 | 67 |
|  | **Chu 2021** | 0.09 | 0.07 - 0.10 | <0.00001 | 74 |
|  | **Cui 2020** | 0.09 | 0.07 - 0.10 | <0.00001 | 72 |
|  | **Du 2018** | 0.09 | 0.07 - 0.10 | <0.00001 | 74 |
|  | **Fu 2016** | 0.09 | 0.07 - 0.10 | <0.00001 | 74 |
|  | **Gui 2017** | 0.09 | 0.07 - 0.10 | <0.00001 | 74 |
|  | **Huang 2018** | 0.09 | 0.07 - 0.11 | <0.00001 | 70 |
|  | **Jiang 2020** | 0.09 | 0.07 - 0.11 | <0.00001 | 74 |
|  | **Li 2019** | 0.08 | 0.07 - 0.10 | <0.00001 | 70 |
|  | **Tu 2017** | 0.08 | 0.07 - 0.10 | <0.00001 | 73 |
|  | **Wang 2018** | 0.08 | 0.07 - 0.10 | <0.00001 | 74 |
|  | **Wang 2020** | 0.09 | 0.07 - 0.10 | <0.00001 | 72 |
|  | **Yan 2017** | 0.09 | 0.07 - 0.10 | <0.00001 | 74 |
|  | **Zhang 2014** | 0.09 | 0.07 - 0.10 | <0.00001 | 73 |
|  | **Zhang 2019** | 0.08 | 0.07 - 0.10 | <0.00001 | 69 |
|  | **Zhang 2021** | 0.08 | 0.07 - 0.10 | <0.00001 | 73 |
|  | **Zhao 2020** | 0.09 | 0.07 - 0.10 | <0.00001 | 74 |
|  | **Zhen 2014** | 0.09 | 0.07 - 0.10 | <0.00001 | 71 |
| Treatment  for < 3 months  (Lumbar spine) | **Chu 2021** | 0.09 | 0.05 - 0.13 | <0.00001 | 64 |
|  | **Cui 2020** | 0.11 | 0.08 - 0.14 | <0.00001 | 0 |
|  | **Yan 2017** | 0.09 | 0.05 - 0.14 | <0.0001 | 62 |
|  | **Zhang 2021** | 0.08 | 0.05 - 0.12 | <0.00001 | 46 |
|  | **Zhao 2020** | 0.09 | 0.05 - 0.13 | <0.0001 | 60 |
| Treatment  for 6 months  (Lumbar spine) | **Bao 2015** | 0.09 | 0.07 - 0.11 | <0.00001 | 74 |
|  | **Du 2018** | 0.08 | 0.06 - 0.10 | <0.00001 | 80 |
|  | **Fu 2016** | 0.08 | 0.06 - 0.10 | <0.00001 | 80 |
|  | **Gui 2017** | 0.08 | 0.06 - 0.10 | <0.00001 | 81 |
|  | **Huang 2018** | 0.09 | 0.07 - 0.11 | <0.00001 | 77 |
|  | **Jiang 2020** | 0.08 | 0.06 - 0.11 | <0.00001 | 80 |
|  | **Li 2019** | 0.08 | 0.06 - 0.10 | <0.00001 | 77 |
|  | **Wang 2018** | 0.08 | 0.06 - 0.10 | <0.00001 | 80 |
|  | **Wang 2020** | 0.08 | 0.06 - 0.10 | <0.00001 | 79 |
|  | **Zhang 2014** | 0.08 | 0.06 - 0.10 | <0.00001 | 80 |
|  | **Zhang 2019** | 0.08 | 0.06 - 0.10 | <0.00001 | 75 |
|  | **Zhen 2014** | 0.09 | 0.07 - 0.11 | <0.00001 | 78 |
| Treatment  for 12 months  (Lumbar spine) | **Tu 2017** | NA | NA | NA | NA |
| Total  (Femoral neck) | **Du 2018** | 0.07 | 0.05 - 0.09 | <0.00001 | 64 |
|  | **Huang 2018** | 0.08 | 0.06 - 0.10 | <0.00001 | 47 |
|  | **Jiang 2020** | 0.07 | 0.05 - 0.10 | <0.00001 | 67 |
|  | **Li 2021** | 0.08 | 0.05 - 0.10 | <0.00001 | 69 |
|  | **Tu 2017** | 0.07 | 0.05 - 0.10 | <0.00001 | 69 |
|  | **Yan 2017** | 0.07 | 0.05 - 0.10 | <0.00001 | 70 |
|  | **Zhang 2019** | 0.07 | 0.04 - 0.09 | <0.00001 | 55 |
|  | **Zhao 2020** | 0.07 | 0.05 - 0.10 | <0.00001 | 67 |
|  | **Zhen 2014** | 0.08 | 0.05 - 0.11 | <0.00001 | 65 |
| Treatment  for < 3 months  (femoral neck) | **Yan 2017** | 0.09 | 0.05 - 0.13 | <0.00001 | NA |
|  | **Zhao 2020** | 0.07 | 0.01 - 0.13 | 0.02 | NA |
| Treatment  for 4-6 months  (femoral neck) | **Du 2018** | 0.07 | 0.03 - 0.10 | 0.0001 | 77 |
|  | **Huang 2018** | 0.08 | 0.05 - 0.12 | <0.00001 | 68 |
|  | **Jiang 2020** | 0.07 | 0.03 - 0.11 | 0.0007 | 78 |
|  | **Li 2021** | 0.08 | 0.04 - 0.12 | 0.0002 | 81 |
|  | **Zhang 2019** | 0.06 | 0.03 - 0.09 | 0.0002 | 65 |
|  | **Zhen 2014** | 0.08 | 0.04 - 0.13 | 0.0001 | 79 |
| Treatment  for 12 months  (femoral neck) | **Tu 2017** | NA | NA | NA | NA |

**Supplementary Table S8.** Sensitivity analysis of effects of XLGB plus ALE on BMD improvement

| BMD site | Study omitted | Pooled estimate | 95% CI | *p* value | I^2^(%) |
| --- | --- | --- | --- | --- | --- |
| Lumbar | <Total> | 0.08 | 0.05 - 0.11 | <0.00001 | 73 |
|  | **Bao 2015** | 0.09 | 0.07 - 0.10 | <0.00001 | 63 |
|  | **Du 2018** | 0.07 | 0.06 - 0.08 | <0.00001 | 76 |
|  | **Fu 2016** | 0.07 | 0.05 - 0.08 | <0.00001 | 76 |
|  | **Li 2019** | 0.06 | 0.05 - 0.08 | <0.00001 | 53 |
|  | **Xu 2009** | 0.07 | 0.06 - 0.09 | <0.00001 | 77 |
|  | **Zhang 2014** | 0.07 | 0.06 - 0.09 | <0.00001 | 77 |
|  | **Zhao 2020** | 0.07 | 0.05 - 0.08 | <0.00001 | 75 |
|  | **Zhao 2020, Li 2019** | 0.06 | 0.04 - 0.09 | <0.00001 | 47 |
| Femoral neck | **<Total>** | 0.11 | 0.05 - 0.17 | 0.0004 | 37 |
|  | **Zhao 2020** | 0.16 | 0.06 - 0.26 | 0.002 | NA |
|  | **Du 2018** | 0.09 | 0.05 - 0.13 | <0.00001 | NA |

**Supplementary Table S9a.** Results of Egger’s test and assessment of heterogenicity

|  | BMD site (OP type) | Subgroup (N. for egger’s test/total data) | p-value (Egger’s test) | Heterogeneity using I^2^ |
| --- | --- | --- | --- | --- |
| **HM+BPs vs BPs** | Lumbar spine | Total (33/33) | 0.959 | 93% |
|  |  | PMOP (12/12) | 0.668 | 97% |
|  |  | Senile OP (21/21) | 0.539 | 81% |
|  | Femoral neck | Total (20/20) | 0.084 | 94% |
|  |  | PMOP (8/8) | 0.008 | 62% |
|  |  | Senile OP (12/12) | 0.061 | 95% |
| **HM+ALE vs ALE** | Lumbar spine | Total (28/28) | 0.516 | 78% |
|  | Lumbar spine (PMOP) | Total (10/10)  3M (4/4) | 0.666  0.652 | 85%  0% |
|  |  | 6M (6/6) | 0.283 | 0% |
|  | Lumbar spine (Senile OP) | Total (18/18) | 0.629 | 72% |
|  |  | Less than 3M (5/5) | 0.458 | 52% |
|  |  | 6M (12/12) | 0.922 | 79% |
|  |  | 12M (1/1) | NA | NA |
|  | Femoral neck | Total (17/17) | 0.001 | 64% |
|  | Femoral neck (PMOP) | Total (8/8) | 0.008 | 62% |
|  |  | Less than 3M (4/4) | 0.102 | 56% |
|  |  | 6M (4/4) | 0.470 | 44% |
|  | Femoral neck (Senile OP) | Total (9/9) | 0.098 | 65% |
|  |  | Less than 3M (2/2) | NA | 0% |
|  |  | 4~6M (6/6) | 0.160 | 76% |
|  |  | 12M (1/1) | NA | NA |
| **XLGB+ALE vs ALE** | Lumbar | Total (7/7) | 0.085 | 73% |
|  |  | Less than 3M (1/1) | NA | NA |
|  |  | 6M (6/6) | 0.160 | 75% |
|  | Femoral enck | Total (2/2) | NA | 37% |
|  |  | Less than 3M (1/1) | NA | NA |
|  |  | 6M (1/1) | NA | NA |
| NA: It is not applicable for Egger’s test due to less than three available data or for heterogeneity using I^2^. | | | |  |

**Supplementary Table S9b**. Correction of the effect sizes by trim-and-fill method

|  | BMD site  (OP type) | Correction | Method | Pooled estimate | 95% CI | | Asymptotic | | Number of studies |
| --- | --- | --- | --- | --- | --- | --- | --- | --- | --- |
|  |  |  |  |  | lower | upper | z value | p value |  |
| HM+BPs *vs* BPs | Femoral neck  (PMOP) | Before | Random | 0.060 | 0.040 | 0.080 | 5.41 | <0.0001 | 8 |
|  |  | After | Random | 0.043 | 0.016 | 0.071 | 3.09 | 0.002 | 11 |
| HM+ALE vs ALE | Femoral neck  (Total) | Before | Random | 0.070 | 0.050 | 0.080 | 8.21 | <0.0001 | 17 |
|  |  | After | Random | 0.049 | 0.030 | 0.068 | 4.94 | <0.0001 | 23 |
